# A new ICA-based fingerprint method for the automatic removal of physiological artifacts from EEG recordings

**DOI:** 10.7717/peerj.4380

**Published:** 2018-02-23

**Authors:** Gabriella Tamburro, Patrique Fiedler, David Stone, Jens Haueisen, Silvia Comani

**Affiliations:** 1 BIND—Behavioral Imaging and Neural Dynamics Center, University “G. d’Annunzio” of Chieti-Pescara, Chieti, Italy; 2 Department of Neurology, Casa di Cura Privata Villa Serena, Città Sant’Angelo, Italy; 3 Institute of Biomedical Engineering and Informatics, Technische Universität Ilmenau, Ilmenau, Germany; 4 Department of Neuroscience, Imaging and Clinical Sciences, University “G. d’Annunzio” of Chieti-Pescara, Chieti, Italy

**Keywords:** EEG, Artifact removal, ICA, IC fingerprint

## Abstract

**Background:**

EEG may be affected by artefacts hindering the analysis of brain signals. Data-driven methods like independent component analysis (ICA) are successful approaches to remove artefacts from the EEG. However, the ICA-based methods developed so far are often affected by limitations, such as: the need for visual inspection of the separated independent components (subjectivity problem) and, in some cases, for the independent and simultaneous recording of the inspected artefacts to identify the artefactual independent components; a potentially heavy manipulation of the EEG signals; the use of linear classification methods; the use of simulated artefacts to validate the methods; no testing in dry electrode or high-density EEG datasets; applications limited to specific conditions and electrode layouts.

**Methods:**

Our fingerprint method automatically identifies EEG ICs containing eyeblinks, eye movements, myogenic artefacts and cardiac interference by evaluating 14 temporal, spatial, spectral, and statistical features composing the IC fingerprint. Sixty-two real EEG datasets containing cued artefacts are recorded with wet and dry electrodes (128 wet and 97 dry channels). For each artefact, 10 nonlinear SVM classifiers are trained on fingerprints of expert-classified ICs. Training groups include randomly chosen wet and dry datasets decomposed in 80 ICs. The classifiers are tested on the IC-fingerprints of different datasets decomposed into 20, 50, or 80 ICs. The SVM performance is assessed in terms of accuracy, False Omission Rate (FOR), Hit Rate (HR), False Alarm Rate (FAR), and sensitivity (*p*). For each artefact, the quality of the artefact-free EEG reconstructed using the classification of the best SVM is assessed by visual inspection and SNR.

**Results:**

The best SVM classifier for each artefact type achieved average accuracy of 1 (eyeblink), 0.98 (cardiac interference), and 0.97 (eye movement and myogenic artefact). Average classification sensitivity (p) was 1 (eyeblink), 0.997 (myogenic artefact), 0.98 (eye movement), and 0.48 (cardiac interference). Average artefact reduction ranged from a maximum of 82% for eyeblinks to a minimum of 33% for cardiac interference, depending on the effectiveness of the proposed method and the amplitude of the removed artefact. The performance of the SVM classifiers did not depend on the electrode type, whereas it was better for lower decomposition levels (50 and 20 ICs).

**Discussion:**

Apart from cardiac interference, SVM performance and average artefact reduction indicate that the fingerprint method has an excellent overall performance in the automatic detection of eyeblinks, eye movements and myogenic artefacts, which is comparable to that of existing methods. Being also independent from simultaneous artefact recording, electrode number, type and layout, and decomposition level, the proposed fingerprint method can have useful applications in clinical and experimental EEG settings.

## Introduction

Electroencephalography (EEG) is a widely used technique to investigate human brain function due to its excellent temporal resolution (Niedermeyer & Da Silva, 2005). Recent advances in compact amplification electronics and preparation-free electrode systems have considerably reduced experimental effort and costs (Liao et al., 2014; Lopez-Gordo, Sanchez-Morillo & Pelayo Valle, 2014; Fiedler et al., 2015) enabling continuous, out-of-the-lab, and mobile EEG acquisition. These advances will enable and simplify new fields of application for EEG acquisition and analysis including brain-computer interfaces (BCI), EEG in sports training and performance monitoring, continuous home side monitoring, and EEG in neuro-motor rehabilitation (Jordan, 2004; Thompson et al., 2008; Askamp & Van Putten, 2014; De Vos et al., 2014; Michel et al., 2015; Lance et al., 2012; Comani et al., 2015; Filho et al., 2016; Di Fronso et al., 2016; Del Percio et al., 2011). Dry-electrode acquisition systems, in particular, have spurred the development of a new generation of mobile EEG applications for monitoring cognition and behavior in real-world environments (Mullen et al., 2015). However, these latest developments have the major potential drawback of higher signal noise due to sensitivity to electrical interference, variable electrode contact quality (Lopez-Gordo, Sanchez-Morillo & Pelayo Valle, 2014), and movement effects. In general, artefacts of biological (e.g., eyeblinks, eye movements, myogenic activity) and non-biological origin (e.g., electrode displacements, electronic interference) can hinder the analysis of EEG signals in most studies (Croft & Barry, 2000; Croft & Barry, 2002; Urigüen & Garcia-Zapirain, 2015). Therefore, there is a crucial need for effective artefact rejection before regular EEG analysis.

Artefact amplitude generally has high variability and can be several orders of magnitude greater than the neural activity of interest. Additionally, the frequency bands of common artefacts often occur within the frequency range of genuine brain signals under investigation which prevents effective pre-processing of EEG recordings based solely on signal filtering (Goncharova et al., 2003). One successful approach to artefact rejection is the application of blind source separation (BSS) methods like independent component analysis (ICA; Delorme, Sejnowski & Makeig, 2007; Jung et al., 2000). By means of this data-driven method, the artefact-affected EEG data are decomposed into a set of source signals whose statistical independence is maximized based on several assumptions, one of which is that most non-cerebral contributions to the mixed signals are independent of neuronal activity. However, the removal of stereotyped and non-stereotyped artefacts generally requires time-consuming visual inspection of the separated independent components (ICs) by a skilled operator. Only after this human intervention, affected by subjectivity-related problems, is it possible to retain ICs related to genuine brain activity for further analysis (Onton et al., 2006).

In order to extend the use of ICA for EEG data pre-processing to non-experts, an automatic parameter-based identification of artefacts is necessary. So far, several approaches have been proposed to address this model-based artefact identification, but none combines all the desirable properties for a fully automatic artefact rejection method, i.e., good performance (accuracy, sensitivity and specificity) on major classes of biological artefacts, good generalization (across sessions, subjects, recording equipment, and sensor layouts), efficiency (low computational cost, potential for online artefact rejection), transparency (use of explicit and physiologically meaningful EEG features), and diffusion (free software with straightforward setup and user interaction).

The first parameter-based artefact detection method was developed for magnetoencephalography (MEG) by Barbati et al. (2004), who used statistical and spectral properties of the recorded signals to detect artefactual components. LeVan, Urrestarazu & Gotman (2006) used statistical, spectral, and spatial features combined with a Bayesian classifier to identify EEG signals and artefacts decomposed by ICA. Halder et al. (2007) first applied a Support Vector Machine (SVM) for the classification of artefactual components in electro-oculogram (EOG) and electromyogram (EMG) recordings, and for the assessment of the performance of different ICA algorithms. In the same year, the parameter-based approach was adapted to the lower sampling frequency and higher spatial resolution of fMRI data by De Martino et al. (2007). These authors first introduced the polar diagram—IC “fingerprints”—for the detection of characteristic artefactual ICs, which were automatically classified using an SVM applying linear discriminant analysis (LDA). This approach was re-introduced in the MEG community by Mantini et al. (2008), who used a single chaos-theory parameter to characterize the artefactual components. Viola et al. (2009) introduced a similar method (CORRMAP) in EEG data pre-processing to detect eye-related artefactual ICs based on the spatial correlation with a user-defined template. Then, Nolan, Whelan & Reilly (2010) applied a mixed approach with features derived from both the EEG time courses and the separated ICs to analyze visual evoked potentials (ERPs). One year later, Mognon et al. (2011) implemented the ADJUST EEGLAB toolbox, which relies on the spatial and temporal features of the EEG data to detect eye-related artefacts without requiring any user intervention. In the same year, Winkler, Haufe & Tangermann (2011) proposed a method, implemented in the EEGLAB plugin SASICA, to automatically select the ICs related to EOG and EMG based on features in the frequency, spatial, and temporal domains.

In 2015 several new methods were introduced. Frølich, Andersen & Mørup (2015) developed a multiclass artefact detection system that uses a multinomial regression classifier based on automatically selected spatial, spectral, and temporal features of the separated ICs. They validated the method across subjects and studies. Although the authors obtained high classification performance, their approach suffered from poor generalizability. Chaumon, Bishop & Busch (2015) proposed an integrative EEGLAB toolbox (SASICA) that encapsulates various methods (ADJUST, MARA and FASTER) in a user-friendly interface to guide experimenter decisions about the rejection of artefactual ICs. Chaumon and colleagues concluded that ICA can fail in separating artefactual from genuine EEG components, although they did not comment on the role played by the number of separated ICs. Radüntz et al. (2015) developed an LDA-based automated artefact removal method that uses spatial information about the ICs and is independent from the direct recording of artefacts. Daly et al. (2015) introduced an online artefact removal method (FORCe) for use in BCI applications which is based on a combination of wavelet decomposition, ICA, and thresholding.

More recently, Chang, Lim & Im (2016) proposed a new method for the real-time detection of eyeblink artefacts that relies solely on an automatic thresholding algorithm, while Kilicarslan, Grossman & Contreras-Vidal, 2016 extended the use of an adaptive noise cancelling scheme for the detection and removal of ocular artefacts and signal drifts, which are the main sources of EEG contamination in brain–machine interface (BMI) applications. Zou, Nathan & Jafari (2016) proposed a method based on the hierarchical clustering of IC features to detect and remove both physiological and non-physiological artefacts from low spatial resolution EEG recordings for BCI applications, and Hou et al. (2016) introduced a modified ICA approach for high density EEG recordings to automatically identify eyeblink components using an artefact relevance index calculated by template matching of each IC. Most recently, Radüntz et al. (2017) extended their earlier method to classify artefactual and non-artefactual ICs by using pre-selected features of the IC topoplot patterns and power spectra as input to several previously trained machine learning algorithms.

Although very promising, the above listed methods are affected by limitations that prevent their general application to EEG datasets recorded with different types of electrodes (wet or dry) and layouts. Indeed, some methods considered only a reduced number of IC features (e.g., Barbati et al., 2004; Halder et al., 2007; Viola et al., 2009; Mognon et al., 2011; Zou, Nathan & Jafari, 2016), heavily manipulated the EEG data to extract the input features to the classifier (Radüntz et al., 2017), or focused on the identification of well-defined artefacts only, such as EMG, EOG and ECG artefacts, often using simultaneously recorded artefactual signals (e.g., Halder et al., 2007; Viola et al., 2009; Nolan, Whelan & Reilly, 2010; Mognon et al., 2011; Winkler, Haufe & Tangermann, 2011; Chang, Lim & Im, 2016; Kilicarslan, Grossman & Contreras-Vidal, 2016; Hou et al., 2016). Other methods were developed for highly specific applications, such as ictal scalp EEG (LeVan, Urrestarazu & Gotman, 2006), visual evoked potentials (Nolan, Whelan & Reilly, 2010), BCI (Daly et al., 2015; Zou, Nathan & Jafari, 2016) and BMI applications (Kilicarslan, Grossman & Contreras-Vidal, 2016). Another common limitation is the prevalent use of simulated artefacts for the validation of the classification system; however, simulated artefacts cannot represent the entire variety of real artefacts possibly affecting brain activity recordings (e.g., Barbati et al., 2004; Halder et al., 2007; Nolan, Whelan & Reilly, 2010; Hou et al., 2016). Finally, some methods adopted a simple linear discriminant approach for the classification of the ICs (e.g., Halder et al., 2007; Radüntz et al., 2015) while others were developed for low-density EEG datasets only (e.g., Zou, Nathan & Jafari, 2016) or were tailored to brain monitoring techniques with low temporal resolution, such as fMRI (e.g., De Martino et al., 2007; Mantini et al., 2008). Some of these authors did not check the quality of the reconstructed artefact-free EEG signals, and none of them tested the effectiveness of their approaches on dry EEG recordings.

We propose a new automatic parameter-based method to identify four physiological artefacts (eyeblinks, eye movements, myogenic activity and cardiac interference) which typically affect EEG recordings. This method consists of the automatic classification of ICs in which EEG recordings have been separated. IC classification uses features of the four main types of physiological artefacts. Our method is based on four assumptions: (1) non-brain contributions to EEG signals are independent from neuronal activity; therefore, they can be separated using a blind source separation approach such as ICA; (2) for a large number of physiological artefacts, artefact-related ICs are characterized by stereotyped features in the parameter hyperspace; (3) while a single feature may not be sufficient to discriminate artefactual from other ICs, the combination of multiple features can efficiently achieve this goal; (4) a nonlinear classification method can be more powerful than linear methods in separating artefactual from other ICs.

As suggested by Urigüen & Garcia-Zapirain (2015), we developed a method that employs multiple processing stages: First, the EEG recordings are decomposed into multiple source signals (ICs) using ICA. Next, several temporal, spatial, spectral and statistical features of the ICs are calculated to form the IC-fingerprints. Then the IC-fingerprints are used as input to a set of nonlinear binary SVMs which automatically classify artefactual ICs (i.e., ICs containing artefacts). A specific SVM classifier is implemented for each type of physiological artefact. Finally, those ICs classified as “other” are used to reconstruct artefact-free EEG signals.

In this paper we introduce the theoretical and methodological aspects of our approach and test our method on real EEG datasets containing cued artefacts which were recorded using two types of electrodes: conventional wet electrodes and novel dry electrodes. We test the performance of our method with respect to (1) different types of electrodes (wet and dry), (2) different number of electrodes in the recording array, and (3) different IC decomposition levels (i.e., the number of ICs generated from the EEG datasets). As an indirect measure of the effectiveness of our denoising method, we evaluate the quality of the reconstructed EEG signal after artefact removal.

## Materials and Methods

### EEG data

A set of EEG recordings which include externally cued artefacts was employed to train and test the performance of our fingerprint method for artefact classification. All EEG acquisitions were performed using a commercially available unipolar biosignal amplifier (RefaExt; Advanced Neuro Technologies B.V., Enschede, Netherlands) with a common average reference and sampled at a frequency of 1,024 samples per second. To assess differences in the performance of our method due to the use of different electrode types (wet or dry) and number, we used EEG datasets which were recorded separately using either a conventional wet electrode cap or a novel dry electrode cap (Fiedler et al., 2015). The commercial wet cap (Waveguard; Advanced Neuro Technologies B.V., Enschede, Netherlands) included 128 Ag/AgCl electrodes arranged in an equidistant layout which were applied in combination with a commercial electrolyte gel (ECI-Electrogel, Electrocap International Inc., USA). The novel dry electrode cap included 97 dry Multipin Polyurethane electrodes with an Ag/AgCl coating arranged in an equidistant layout. The layout of both caps is provided in supplemental Fig. S1. For both cap types, conventional ring-shaped Ag/AgCl electrodes were applied at the right mastoid position in combination with electrolyte gel for amplifier ground.

EEG datasets including cued artefacts (eyeblinks, eye movements, muscle activity—i.e., jaw muscle contractions) were recorded using both wet and dry caps. Obviously, no EEG datasets including cued cardiac interference could be recorded, so datasets recorded for the other physiological artefacts were used for the detection of cardiac interference as described in ‘Supervised SVM testing (automatic classification of testing sets)’. No simultaneous direct recording of artefactual signals (i.e., with EMG, EOG or ECG) was performed. The Ethics Committee of the University “G. d’Annunzio” of Chieti-Pescara (Italy) approved the study (Ethical Application Ref. n. 10-21/05/2015). All participants gave their written informed consent prior to participation in the study.

*Eyeblinks:* EEG acquisitions included 50 eyeblinks cued with a beep tone at intervals of 5 s for a total duration of 250 s. Twelve subjects (male only, age: 28.5 ± 2 years) participated in these acquisitions for a total of 24 datasets (12 with wet electrodes and 12 with dry electrodes).

*Eye movements:* Horizontal eye movements were acquired in volunteers seated facing a 16:9 monitor with 30 inch diameter (Myrica V30–1, Fujitsu Siemens, Japan). A fixed chin rest was used to ensure a screen-eye distance of 50 cm. The corresponding visual field of the screen was approximately 64 degrees in the horizontal direction and 42 degrees in the vertical direction. Volunteers were asked to follow the position of a red cross on the screen, which initially appeared center screen and was subsequently moved transiently in a repeated deterministic sequence of left side of screen movement, right side of screen movement, bottom side of screen movement, and top side of screen movement, returning to center screen after each movement. Each movement corresponded to a position change of approximately 16° within the visual field of the participant. Individual movements were cued with beep tones at 2 s intervals for a total of seven complete sequences, with inter-sequence intervals of 8 s (total duration = 112 s). Nineteen volunteers (male only, age: 28.8 ± 1.9 years) participated in these acquisitions for a total of 19 datasets (10 with wet electrodes and 9 with dry electrodes).

*Myogenic artefact:* Myogenic contractions of the *musculus masseter* (jaw muscle) were recorded. Volunteers were asked to maximally contract these muscles at the first tone and to keep the contraction until the next tone. They relaxed the muscles until the subsequent tone. Tones were delivered using either 2 s inter-tone intervals for 11 contractions (average total duration: 44 s) or 3 s inter-tone intervals for 7 contractions (average total duration: 42 s). Nineteen volunteers (male only, age: 26.7 ± 1.2 years) participated in these acquisitions for a total of 19 datasets (10 with wet electrodes and 9 with dry electrodes).

The individual EEG recordings may have slightly different total durations because baseline EEG may have been recorded before and after the cued artefacts. The raw EEG data are available as supplemental files, and the links to the folders are given in the supplemental file “Raw data—Information”.

### Data pre-processing and overview of the method

**Figure 1:**
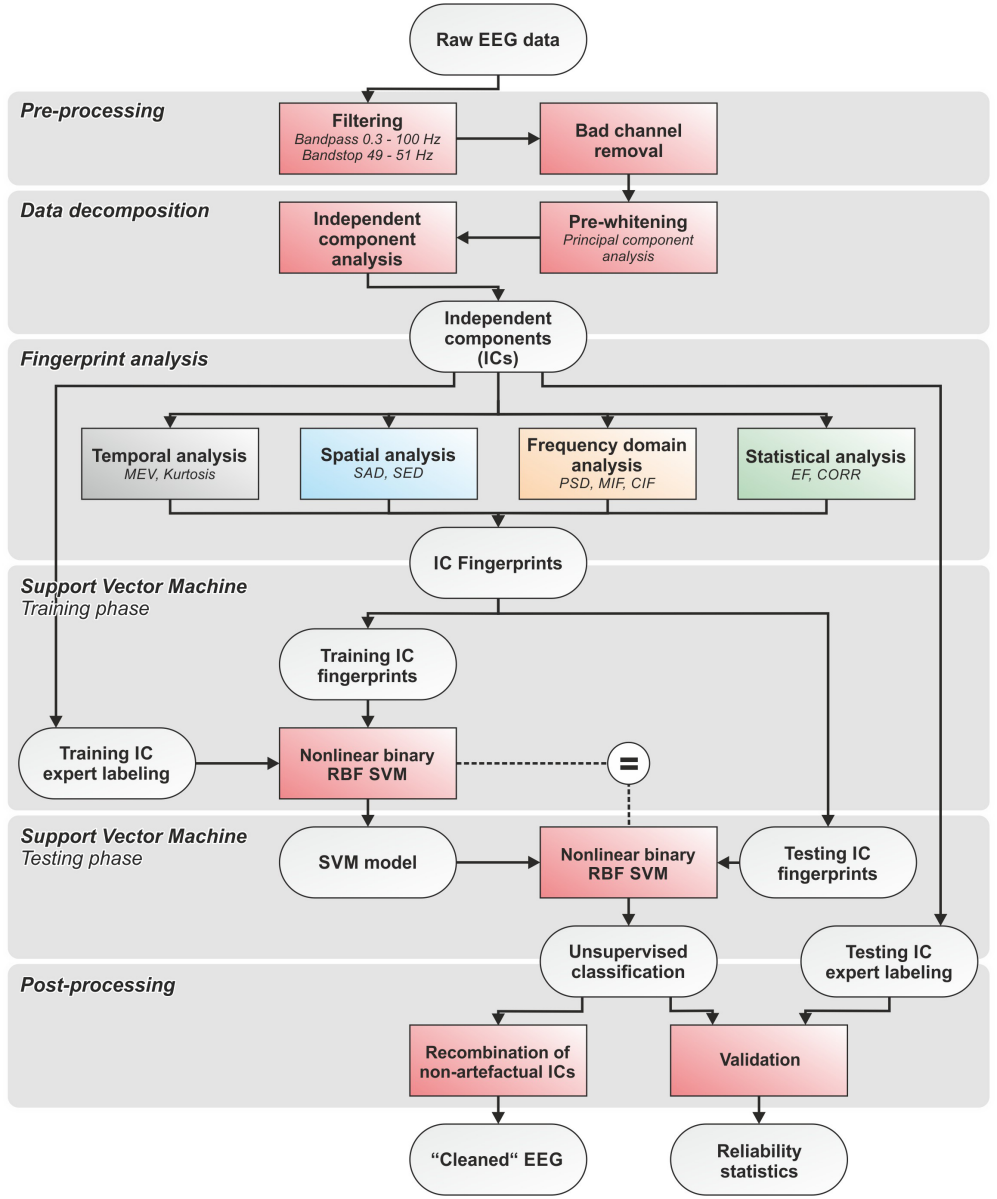
Flowchart of the complete data processing. Flowchart of the complete data processing pipeline including six sequential phases of data processing and their respective modular processing steps, input and output.

First, all EEG recordings were bandpass filtered with cut-off frequencies at 0.3 and 100 Hz. A bandstop filter at 50 Hz was applied to eliminate power line interferences. We used zero-phase Hamming-windowed sinc FIR filters applied using the firfilt EEGLAB plugin (Widmann, Schröger & Maess, 2015). Bad channels (i.e., channels exhibiting isoelectric saturation or contaminated with excessive artefacts and noise during >50% of the recording time) were identified by an expert operator and excluded from further analysis (McMenamin et al., 2010). Second, filtered EEG data were pre-whitened using Principal Component Analysis (PCA), then decomposed using ICA (see ‘EEG data decomposition using independent component analysis (ICA)’). Third, the IC-fingerprint was calculated for each IC using our set of features (see ‘Definition of the IC-fingerprint’). Fourth, separate nonlinear radial basis function binary SVM classifiers were trained for each artefact type. We adopted an approach that permits the detection and rejection of the four physiological artefacts independently and in series (see ‘Automatic artefact classification’). Fifth, the SVM classifiers were tested on separate IC-fingerprint sets in a fully automatic manner. Sixth, cross-validation was performed using different combinations of randomly chosen training and testing datasets. The performance of the SVM classifiers was statistically validated by comparing automatic SVM classification with expert labeling (see ‘Statistical assessment of the performance of the SVM classifiers’). The quality of the reconstructed artefact-free EEG signals was evaluated by visual inspection and comparison of the signal-to-noise ratio (SNR) before and after artefact removal (see ‘Assessment of signal quality in artefact-free EEG’). The code was developed using Matlab (release MatlabR20014a; MathWorks, Natick, MA, USA) and EEGLAB (release 13.3.2b; Delorme & Makeig, 2004). An overall flow chart of the complete data processing pipeline is shown in Fig. 1. The pipeline and the fingerprint classification system were designed to ensure modularity in terms of analyzed artefacts and number and type of features used to calculate the IC-fingerprint.

### EEG data decomposition using independent component analysis (ICA)

Due to volume conduction, EEG signals measured on the scalp derive from multiple brain and non-brain sources of electrical activity. Given that electrical signals sum up at each electrode site with no time delay, this process can be modeled by a linear instantaneous mixture. ICA is a blind source separation technique widely used in the processing of mixtures of multivariate signals (Bell & Sejnowski, 1995; Comon, 1994). ICA assesses the statistical independence of the sources of signals linearly mixed in multiple spatially distributed recordings and reconstructs their time courses. It is widely accepted that any given artefact is independent from genuine brain signals and other artefacts affecting the data mixtures. Therefore, ICA is an adequate method for extracting the time course of artefactual sources in electroencephalographic data (Vorobyov & Cichocki, 2002; Jung et al., 2000; Karhunen et al., 1997; Lee, Girolami & Sejnowski, 1999; Vigário et al., 2000). Among the most common ICA algorithms used for EEG data decomposition, we used Infomax ICA (Bell & Sejnowski, 1995; Lee et al., 2000) which shows good performance in decomposing both physiologically plausible projections of brain EEG sources (Delorme et al., 2012) and artefacts in EEG data (Delorme, Sejnowski & Makeig, 2007).

A condition for the correct application of ICA is that the data mixture, modeled as a matrix, must be considered invariable during the recording. This condition is related to the assumption that the mixed signals are stationary (Bell & Sejnowski, 1995; Comon, 1994). However, physiological and artefactual signals often produce mixtures of multivariate signals that are non-stationary, hence their proportions in the mixture can change over time. Nevertheless, the assumption of signal stationarity is satisfied when short signal segments (e.g., recordings of short duration) are analyzed (Comani et al., 2004a; Comani et al., 2004b; Murta Jr et al., 2015). On the other hand, according to Delorme & Makeig (2004) the analysis of short signal segments can prevent the separation of a high number of ICs. To overcome this problem, Principal Components Analysis (PCA) was performed to reduce data dimensionality (Delorme, Sejnowski & Makeig, 2007) before applying ICA. Therefore, we could perform the ICA on EEG segments sufficiently short to be considered stationary and also obtain a sufficiently large number of ICs. Given the short duration of EEG recordings, we applied ICA to the entire pre-processed EEG time course and assumed the condition of signal stationarity was satisfied. All datasets were decomposed into 20, 50 and 80 ICs to simulate typical experimental conditions:

• 20 ICs mimic the decomposition level possible with clinical systems (which typically have 20 electrodes with generally good and constant contact) and with caps of 32 electrodes (assuming some electrodes may be excluded for bad contacts);
• 50 ICs mimic a decomposition level possible with caps of 64 electrodes (considering that some electrodes could be excluded for bad contacts);
• 80 ICs mimic a decomposition level possible with caps of 90 or more electrodes (for which some electrodes can have bad contacts).

### Definition of the IC-fingerprint

Based on the concept that each signal (and therefore each artefact) is characterized by a unique ensemble of temporal, spatial, spectral and statistical features, 14 features are calculated for each IC which span signal properties across the four domains. With this approach, each IC is represented as a point in the 14-dimensional feature space where the point coordinates are the values of each of the 14 features. We expect that ICs containing the same type of artefact will have similar values and will therefore have a similar fingerprint. Some features refer to previously introduced measures of specific artefacts such as eyeblinks and eye movements (see the definition of the temporal and spatial features in ‘Temporal features’ and ‘Spatial features’), whereas other features are new and specifically conceived to detect myogenic and cardiac contamination (see the definition of the spectral features in ‘Spectral features’). The statistical features (‘Statistical features’) are used to identify specific waveforms or a structure in the separated ICs.

**Figure 2:**
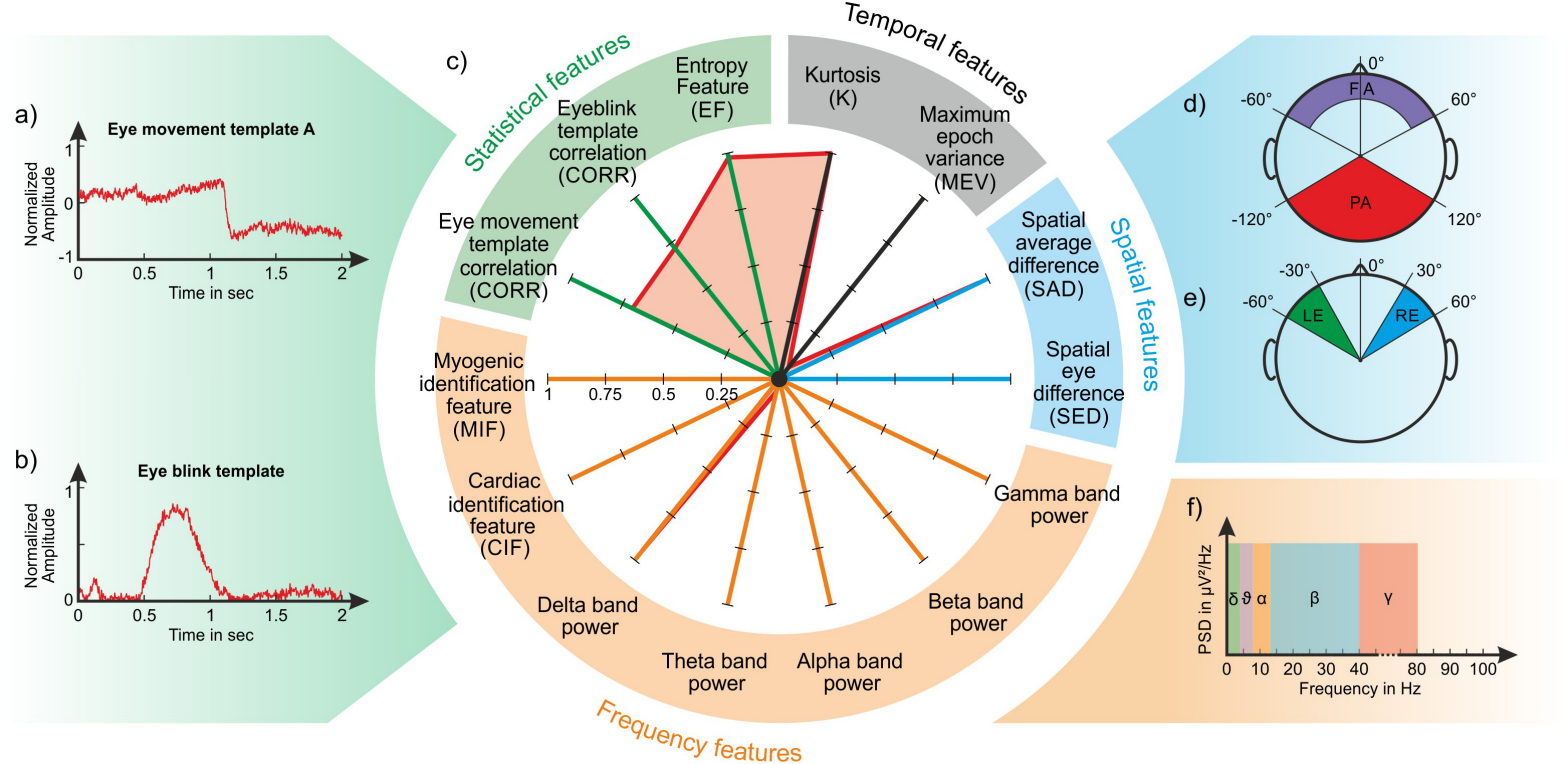
Composition of an IC fingerprint. Artifact templates used to calculate the CORR features (A: horizontal eye movement; B: eyeblink); Exemplary IC fingerprint containing eyeblinks (C); Schematic representation of scalp areas used to calculate the spatial features (D) Spatial Average Difference (SAD); (E) Spatial Eye Difference (SED); the five frequency bands considered to calculate the PSD features (F).

The temporal and spatial features are based on the normalized IC weights to enhance the different contribution of individual ICs to the recorded EEG datasets. All features are represented in a polar plot (Fig. 2C) where each axis corresponds to an individual feature whose value ranges from 0 to 1. Certain features range from 0 to 1 by definition, (e.g., CIF and MIF, see ‘Spectral features’), whereas others are normalized between 0 and 1 using the methods described in detail in the dedicated sections (see ‘Temporal features’ and ‘Spatial features’).

#### Temporal features

Consecutive epochs of 5 s with a one sec overlap are used to segment the time course of individual ICs for the calculation of the two temporal features.

The Temporal Kurtosis, *K* is calculated according to Eq. (1):

(1) \begin{eqnarray*}K= \frac{1}{m} \sum _{e=1}^{m} \left( \frac{ \frac{1}{n} \sum _{i=1}^{n}{ \left( {s}_{e,i}-\overline{{s}_{e}} \right) }^{4}}{{ \left( \frac{1}{n} \sum _{i=1}^{n}{ \left( {s}_{e,i}-\overline{{s}_{e}} \right) }^{2} \right) }^{2}} -3 \right) \end{eqnarray*}

where the parameters *s*_*i*_ denote the *i*th out of *n* data samples in the *e* epoch data vector, and *m* is the number of epochs in the IC time course. To minimize the influence of signal drifts and offset, for each epoch the respective epoch mean is subtracted prior to the calculation of *K* (Mognon et al., 2011). Given that Kurtosis is positive for rapid signal amplitude variations, only positive *K* values are retained (Greco et al., 2006). The *K* values of all ICs are then normalized by the maximum value to a range of 0 to 1. *K* is particularly suitable to identify artefacts like eyeblinks and rapid eye movements that typically generate transient amplitude changes within the EEG signals (Barbati et al., 2004; Delorme, Westerfield & Makeig, 2007).

The Maximum Epoch Variance (MEV) is calculated according to Eq. (2) (Mognon et al., 2011), where *max*()_*e*_ denotes the maximum across all epoch values.

(2) \begin{eqnarray*}MEV= \frac{max{ \left( \frac{1}{n} \sum _{i=1}^{n}{ \left( {s}_{e,i} \right) }^{2}-{ \left( \frac{1}{n} \sum _{i=1}^{n}{s}_{e,i} \right) }^{2} \right) }_{e}}{ \frac{1}{m} \sum _{e=1}^{m} \left( \frac{1}{n} \sum _{i=1}^{n}{ \left( {s}_{e,i} \right) }^{2}-{ \left( \frac{1}{n} \sum _{i=1}^{n}{s}_{e,i} \right) }^{2} \right) } .\end{eqnarray*}

As for *K*, all MEV values of all ICs are normalized with respect to the maximum MEV value. MEV is more suitable to detect eye movements, which often exhibit lower amplitude and less transient changes.

#### Spatial features

Eyeblinks and eye movements are also characterized by large differences in the EEG recorded in the frontal and temporal areas as compared to the posterior areas. Consequently, the weights of ICs related to eyeblinks and eye movements show a characteristic distribution on the scalp that represents the different contribution of these artefactual components to the EEG signals recorded at various electrode positions.

**Figure 3:**
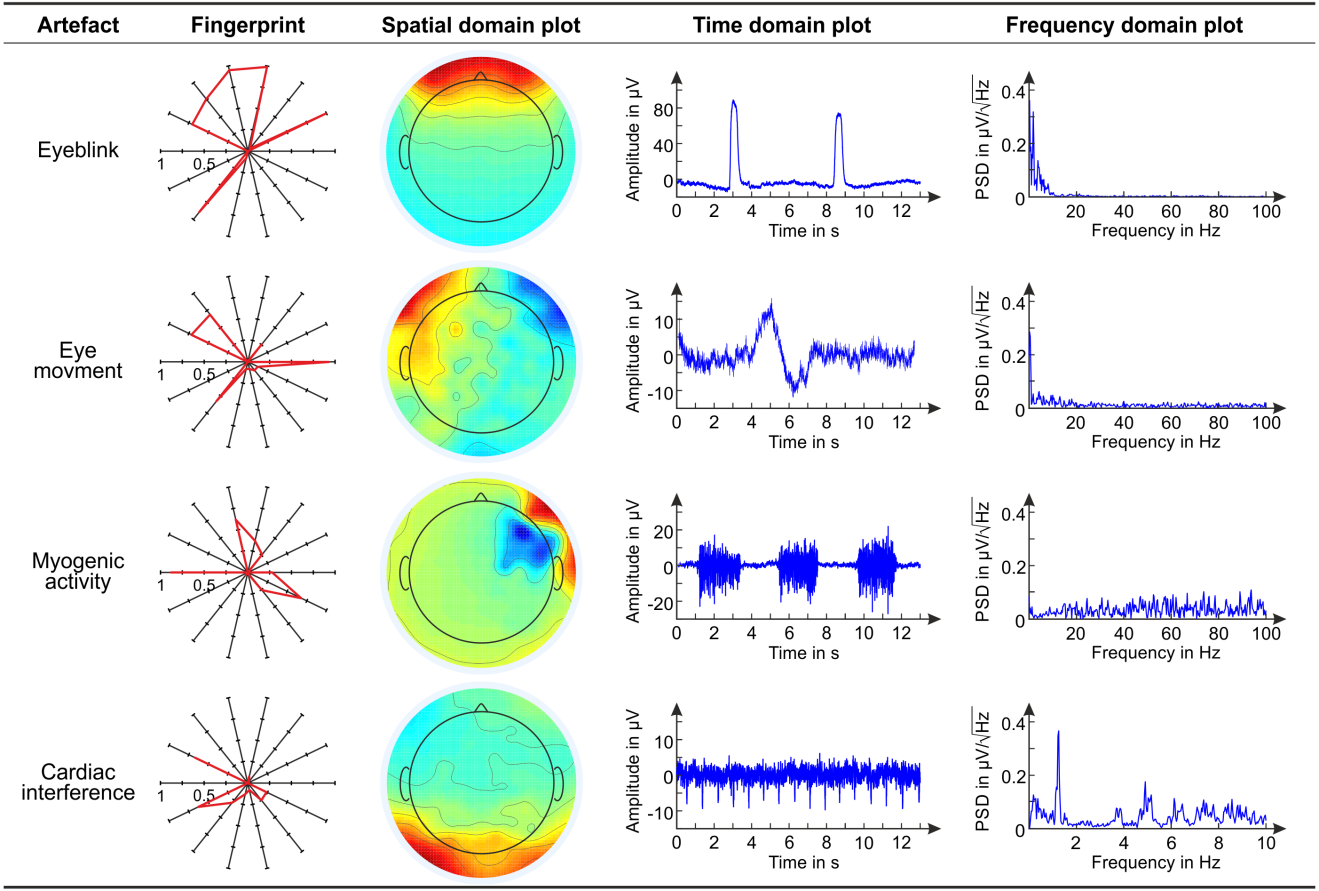
Main characteristics of exemplary artifactual ICs. Overview of the main characteristics of exemplary artifactual ICs. For each artifact, we show the fingerprint, the topoplot, the time course and the power spectrum of an exemplary artifactual IC. In the polar plots representing the fingerprints of individual ICs, the bold line connects the values of the features. These examples refer to EEG datasets recorded with conventional wet electrodes.

**Figure 4:**
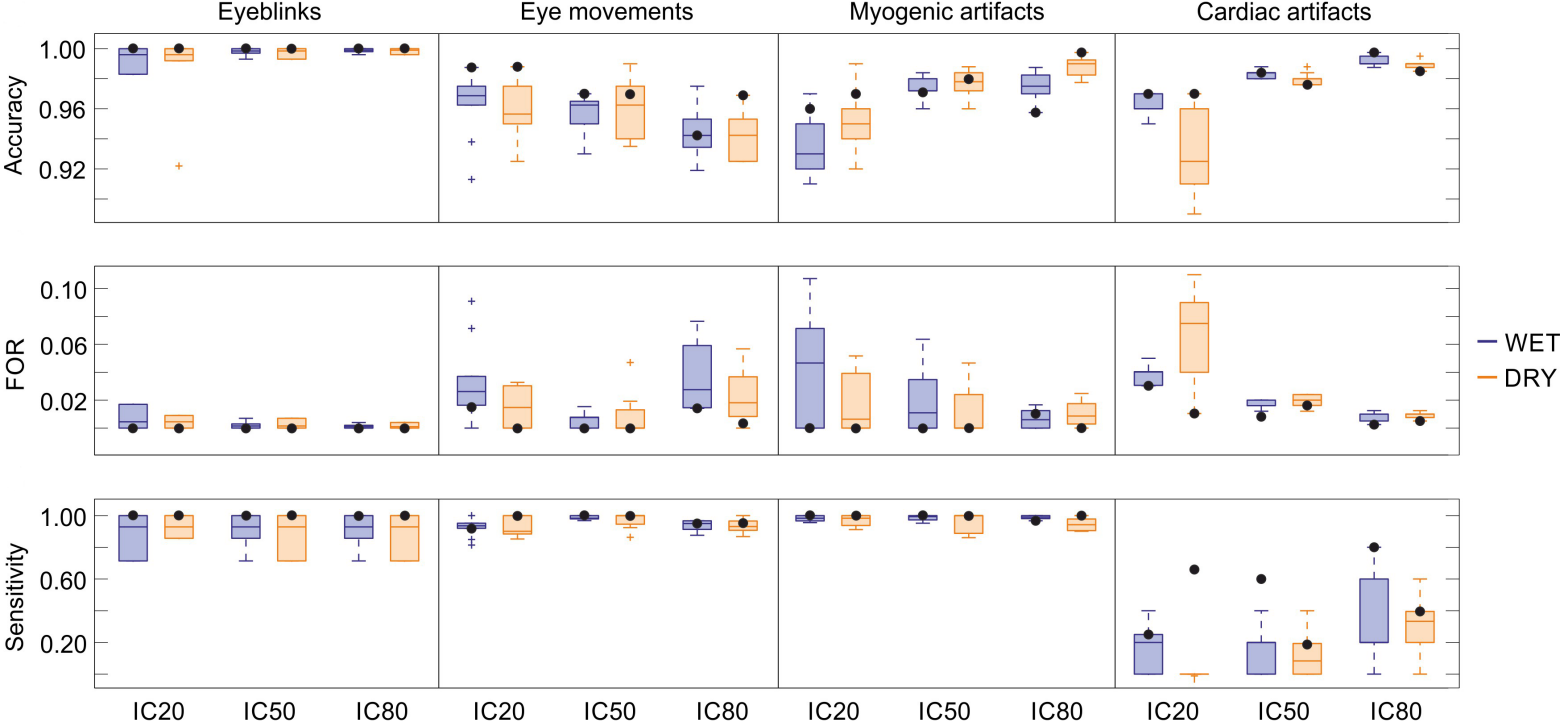
Descriptive statistics of the cross-validation for all SVM classifiers. Descriptive statistics of the main performance measures (accuracy, FOR, sensitivity) of the ten SVM classifiers trained for each artifact type. The values of accuracy, FOR and sensitivity obtained for the SVM classifiers with the best performance are indicated with black dots. Results refer to the testing datasets and are given for each decomposition level (20, 50 and 80 ICs) and separately for wet and dry EEG datasets (color-coded). In the boxplots: the central line indicates the median; the box bottom/top indicate the 25th and 75th percentile respectively; the whiskers extend to the most extreme data points that are not outliers; the crosses indicate outliers. Complete performance measures of the ten SVM classifiers for each artefact type are reported in Tables S2–S5.

The Spatial Average Difference (SAD) and the Spatial Eye Difference (SED) (Mognon et al., 2011) are calculated to assess the spatial distribution of the IC weights over the scalp by grouping weights with respect to the *k* electrode positions. Four areas are defined to calculate these features: (1) the frontal area (FA), including electrodes whose angular positions range from 0° to 60° from the medial line and have a radial range ≥0.4 (cp. Fig. 2D); (2) the posterior area (PA), including electrodes whose angular positions range from 0° to 120° from the medial line and have a radial range of 1 (cp. Fig. 2D); (3) the left area (LE), including electrodes whose angular positions range from −60° to −30° (cp. Fig. 2E); (4) the right area (RE), including electrodes whose angular positions range from 30° to 60° (cp. Fig. 2E).

The Spatial Average Difference (SAD) is tailored to detect eyeblinks, and is calculated according to the following equation:

(3) \begin{eqnarray*}SAD= \left\vert \frac{1}{k} \sum _{e=1}^{k}{a}_{k,FA} \right\vert - \left\vert \frac{1}{k} \sum _{e=1}^{k}{a}_{k,PA} \right\vert \end{eqnarray*}

where *a* is the vector of the IC weights in the *k* electrode positions in FA and PA.

Before calculating SAD, we assess that the weight variations over the scalp are really due to eyeblinks. First, we check the difference between the variance associated with frontal electrode weights and the variance associated with posterior electrode weights. If this difference is ≤0, it means that SAD is due to a posterior source and not to eyeblinks. In these cases, SAD is set to 0. Second, we compare the sign of the average weights in the LE and RE areas. If they have different signs, it means that there is a net horizontal eye movement (a different artefact) and SAD is set to 0. In all other cases, SAD is calculated according to Eq. (3).

The Spatial Eye Difference (SED) assesses the difference between the IC weights in the LE and RE areas to detect horizontal eye movements. It is calculated according to:

(4) \begin{eqnarray*}SED= \left\vert \frac{1}{k} \sum _{e=1}^{k}{a}_{k,LE}- \frac{1}{k} \sum _{e=1}^{k}{a}_{k,RE} \right\vert \end{eqnarray*}

where, again, *a* is the vector of the IC weights in the *k* electrode positions within the LE and RE areas. To verify that the weight variations are really due to eye movements, we compare the sign of the average weights in the LE and RE areas. If the average weights have the same sign, SED is not due to eye movements, and it is set to 0. In all other cases, SED is calculated according to Eq. (4). Finally, the SAD and SED values are normalized, respectively, to the maximum SAD and SED values across all ICs.

#### Spectral features

*PSD in the five main EEG bands:* Given that the Power Spectral Density (PSD) provides a compact representation of the energy distribution of the EEG signal (Welch, 1967; Freeman et al., 2003), for each IC we calculate the mean PSD in the following frequency bands: Delta band [0.3–4] Hz, Theta band (4–8] Hz, Alpha band (8–12] Hz, Beta band (12–40] Hz, Gamma band (40–100] Hz (see Fig. 2F). The five frequency features are calculated as the mean PSD in the defined frequency bands normalized to the maximum PSD across all bands.

*Cardiac identification feature—CIF:* This feature is intended to identify ICs related to the cardiac interference without using co-registered electrocardiograms. The hypothesis is that an IC related to cardiac interference should show a peak corresponding to the cardiac frequency of the subject in the PSD distribution within a frequency band defined according to the recording conditions. We define the frequency band of interest as 0.8–1.7 Hz, because it corresponds to a cardiac frequency range from 48 beats per minute to 102 beats per minute. This interval includes a wide range of cardiac frequencies for an adult population in rest conditions. We calculate the PSD in a frequency band (0.3–8 Hz) that is larger than the frequency band of interest and search for the maximum power peak in this range. If the maximum power peak is not identified in the 0.8–1.7 Hz range, the analyzed IC is considered not to be related to cardiac interference, and *CIF* is set to 0. If the maximum power peak is included in the 0.8–1.7 Hz range, the IC could be related to the cardiac frequency, and its time course is analyzed. First, we identify all the peaks in the IC time course and select those that (a) are greater than half the average peak amplitude of all found peaks and that (b) occur at a distance corresponding to the inter-beat interval (in samples) as expected on the basis of the maximum power peak. The cardiac identification feature (*CIF*) is then calculated as:

(5) \begin{eqnarray*}CIF= \frac{{N}_{fcp}}{{N}_{ecb}} \end{eqnarray*}

where *N*_*fcp*_ is the number of peaks in the IC time course that satisfy conditions a and b, and *N*_*ecb*_ is the number of expected cardiac beats on the basis of the found maximum power peak in the frequency band of interest (0.8–1.7 Hz). If the analyzed IC is a cardiac artefactual component, *CIF* should be close to 1. Given that *CIF* can range from 0 to 1, no normalization is performed.

*Myogenic identification feature—MIF:* Although some researchers have observed EMG contamination in the EEG at frequencies below 20 Hz (Friedman & Thayer, 1991), myogenic interference typically have frequency components above 20 Hz (Whitham et al., 2007; Goncharova et al., 2003). Indeed, during the expert classification of the ICs, those containing myogenic artefacts always showed a negligible frequency content above 20 Hz. Therefore, for each IC we calculate the PSD in two frequency bands: 0–20 Hz (PSD_0−20_) and 21–100 Hz (PSD_21−100_). If PSD_0−20_ is greater than PSD_21−100_, we consider that the analyzed IC does not contain myogenic artefacts and *MIF* is set to 0. Otherwise, the myogenic identification feature (*MIF*) is calculated as:

(6) \begin{eqnarray*}MIF= \frac{\sum _{21\mathrm{Hz}}^{100\mathrm{Hz}}\mathrm{PSD}}{\sum _{0\mathrm{Hz}}^{20\mathrm{Hz}}\mathrm{PSD}+\sum _{21\mathrm{Hz}}^{100\mathrm{Hz}}\mathrm{PSD}} .\end{eqnarray*}

For ICs containing myogenic artefacts, *MIF* should be close to 1. Given that *MIF* can range from 0 to 1, no normalization is performed.

#### Statistical features

Two statistical features are considered: a feature based on the correlation between the IC time course and the template of the expected artefact waveform, and a feature based on the evaluation of the structure of the IC time course using an entropy estimator.

*Correlation feature—CORR:* Some artefacts like eyeblinks and eye movements exhibit a characteristic waveform. We calculated characteristic templates for eyeblinks and horizontal eye movement artefacts using one channel from one dataset for each artefact (the channel and dataset where the artefact was most visible). The artefact templates were calculated using windows of 2 s (Figs. 2A and 2B). For horizontal eye movements, we used only the template of the left-to-right direction because the template for the opposite direction is simply in anti-phase. Given that the CORR feature is based on absolute correlation values, the use of one template is sufficient. The template is compared to the IC time course using a moving window of 2 s that is shifted by 1 ms until the entire IC time course is spanned, obtaining a vector of Pearson product moment correlation coefficients. We then retain only the correlation coefficients with absolute value ≥0.65 and the correlation feature CORR is calculated as the average of the retained absolute correlation values:

(7) \begin{eqnarray*}\text{CORR}= \frac{\sum _{i=1}^{N} \left( {r}_{i} \right) }{N} \end{eqnarray*}

where *r*_*i*_ is the absolute value of the retained correlation coefficient for the *i*th IC window and *N* is the total number of retained correlation values. If no absolute correlation value ≥ 0.65 is found, then CORR is set to 0.

*Entropy feature—EF:* Entropy is a statistical measure of self-similarity in a given signal, and is useful to estimate the irregularity of short and noisy signals of biological systems that include both deterministic and stochastic processes (Pincus, 1991). According to Barbati and colleagues (2004) and Greco and colleagues (2006), artefactual and other ICs can be differentiated by using an approximate measure of entropy of the *jth* 5 s segment of the *ith* IC, defined as follows:

(8) \begin{eqnarray*}{H}^{i}=-\sum _{x\in j}{p}_{j}^{i}(x)\log \nolimits ({p}_{j}^{i} \left( x \right) )\end{eqnarray*}

where ${p}_{j}^{i} \left( x \right) $ is the probability of observing the activity values *x* in the distribution of activity in the *jth* 5 s segment of the *ith* IC. After calculating these entropy measures for all segments and all ICs, we normalize the segment-entropy measures to 0-mean and 1-standard deviation for each segment across all ICs. For each IC we then calculate how many entropy measures are ≥1.64 or ≤ − 1.64 (Barbati et al., 2004). The entropy feature EF is defined as:

(9) \begin{eqnarray*}EF= \frac{{\mathrm{N}}_{\mathrm{sig}}}{{\mathrm{N}}_{\mathrm{tot}}} \end{eqnarray*}

where N_sig_ is the number of entropy measures ≥1.64 or ≤ − 1.64, and *N*_tot_ is the total number of entropy measures for the given IC. When EF is ≤0.2, EF is set to 0.

### Automatic artefact classification

Artefact classification is performed by means of Support Vector Machines (SVMs). Originally, supervised SVMs are binary linear classifiers that, given a set of training examples preliminarily marked as belonging to one or the other of two classes, build a model able to assign new data to one of the two classes. An SVM model is a representation of the examples as points in a feature space, mapped so that the examples of the two classes are separated by a gap that is as wide as possible. New data are mapped into that feature space and assigned to one of the two classes based on which side of the gap they fall. If several hyperplanes exist that achieve the separation of the examples in two classes, generally the maximum-margin hyperplane is chosen because it ensures the largest separation between the classes (Vapnik, 1995; Vapnik, 1998).

When input data are not linearly separable in their original feature space, it is possible to create a nonlinear SVM classifier by applying the kernel trick (Aizerman, Braverman & Rozonoer, 1964) to the maximum-margin hyperplane, which is then fit in a transformed higher dimensional feature space (Jin & Wang, 2012). Working in a higher-dimensional feature space generally increases the generalization error of SVMs. However, if enough training samples are provided, the algorithm still performs well (Jin & Wang, 2012). The most common kernels are linear, polynomial, radial basis function, and sigmoid (Burges, 1998). It is worth mentioning that linear methods may yield poor predictive accuracy for low-dimensional problems with many training instances, whereas the use of nonlinear kernels can lead to a computationally intractable problem and to overfitting (Bishop, 2006; Cawley & Talbot, 2010). However, linearly non-separable features often become linearly separable after they are mapped to a high dimensional feature space.

For this reason and given the characteristics of the EEG data to be analyzed and the number of features calculated for each IC, we chose to employ nonlinear binary SVMs with a radial basis function kernel (RBF or Gaussian kernel), as these allow the projection of Euclidean vectors into an infinite dimensional Euclidean space. To avoid uncontrolled results, we trained and tested 10 individual nonlinear binary RBF SVM classifiers for each of the four physiological artefacts considered.

#### Supervised SVM training

For each physiological artefact to be classified, 10 individual SVMs were trained. The supervised training of the SVMs used sets of fingerprints calculated for ICs previously labeled by two independent experts as “artefactual” if they included the artefact of interest, or as “other” if they did not. In case of discordance, the experts met and achieved an agreement on the IC labeling. Each dataset was always decomposed into 80 ICs to have the highest number of IC-fingerprints for the SVM training.

For each physiological artefact, each of the ten SVMs was trained on a different group of datasets randomly chosen from the cued EEG datasets described in ‘EEG data’.

Out of all the possible combinations of EEG datasets (wet and dry separately), we selected training groups as varied as possible in terms of combinations of wet and dry datasets. The training groups always included both wet and dry EEG recordings, and each SVM was always trained using the IC-fingerprints obtained from both wet and dry datasets. The training groups were composed as follows: for eyeblink SVMs, six wet and six dry EEG datasets with cued eyeblinks; for eye movements SVMs, six wet and five dry EEG datasets with cued eye movements; for myogenic artefact SVMs, five wet and four dry EEG datasets with cued myogenic artefacts. For the training of the cardiac interference SVMs we used ICs that the experts classified as including cardiac interference during the labeling of the ICs separated from the EEG datasets containing cued eyeblinks and eye movements. Overall, 24 datasets contained ICs related to cardiac activity, and each training group for the cardiac interference SVMs always included eight wet and six dry EEG datasets. For each artefact, the datasets used for the training of each SVM are listed in Table S1.

#### Supervised SVM testing (automatic classification of testing sets)

For each artefact type, each of the 10 trained SVM classifiers was tested on the fingerprints of the ICs separated from the wet and dry EEG datasets not included in its training group. Testing of the SVMs consisted in the completely automatic classification of the ICs. To verify whether the performance of the SVM depended on the decomposition level, each SVM was tested separately for testing datasets decomposed into either 20, 50 or 80 ICs. The SVM classification results were then compared with expert classification of the same ICs (see ‘Statistical assessment of the performance of the SVM classifiers’).

The groups of testing datasets always included: for eyeblink SVMs, 6 wet and 6 dry EEG datasets with cued eyeblinks; for eye movements SVMs, 4 wet and 4 dry EEG datasets with cued eye movements; for myogenic artefact SVMs, 5 wet and 5 dry EEG datasets with cued myogenic artefacts; for cardiac interference SVMs, 5 wet and 5 dry EEG datasets with cued eyeblinks and eye movements (those having ICs related to cardiac activity). For each artefact, the datasets used for the testing of each SVM are listed in Table S1.

### Statistical assessment of the performance of the SVM classifiers

For each artefact, the performance of the 10 SVM classifiers was statistically evaluated separately for the two types of EEG recordings (wet or dry) and for the different IC decomposition levels (20, 50 or 80 ICs). We calculated the true positive (TP), false positive (FP), true negative (TN) and false negative (FN) classifications of all testing ICs with respect to the labeling performed by the two expert operators. We calculated the accuracy and false omission rate (FOR) of the classification results (Eqs. (10) and (11)) to estimate the degree of agreement between the classification results and true classification (as determined by expert classification) and the probability that artefactual ICs were misclassified by the SVM.

(10) \begin{eqnarray*}& & \text{Accuracy}= \frac{\text{True Positives}+\text{True Negatives}}{\text{All cases}} \end{eqnarray*}

(11) \begin{eqnarray*}& & \mathrm{FOR}= \frac{\text{False Negatives}}{\text{False Negatives}+\text{True Negatives}} .\end{eqnarray*}

We also calculated the Hit Rate (HR), defined as the number of correctly classified artefactual ICs over the total number of artefactual ICs, and the False Alarm Rate (FAR), defined as the number of ICs misclassified as “other” over the total number of “other” ICs:

(12) \begin{eqnarray*}& & \mathrm{HR}= \frac{\text{True Positives}}{\text{True Positives}+\text{False Negatives}} \end{eqnarray*}

(13) \begin{eqnarray*}& & \mathrm{FAR}= \frac{\text{False Positives}}{\text{False Positives}+\text{True Negatives}} .\end{eqnarray*}

Given that our classification is categorical, the sensitivity of the SVM in separating artefactual from “other” ICs was calculated as the quantity *p*:

(14) \begin{eqnarray*}p= \frac{\mathrm{HR}-\mathrm{FAR}}{1-\mathrm{FAR}} \end{eqnarray*}

where FAR was used as a measure of bias. Within the high threshold model (HTM), *p* is the sensitivity of the sensory process and FAR is the guessing rate of the decision process (National Research Council (US) Committee on Vision, 1985). In the case of perfect classification, accuracy, HR and *p* equal 1, and FOR and FAR equal zero.

### Assessment of signal quality in artefact-free EEG

The effectiveness of our proposed method in reducing physiological interferences in EEG recordings was assessed by estimating the quality of the reconstructed artefact-free EEG signals. For each artefact type, we reconstructed the artefact-free EEG signals for each testing dataset (both wet and dry) and decomposition level. The ICs classified as artefactual by the SVM which had the best performance were disregarded, and the artefact-free EEG signals at each electrode location were reconstructed by re-projecting all the other ICs back into sensor space. The amount of residual contamination in the artefact-free EEG signals was evaluated by visual inspection and quantified as the change of the signal-to-noise ratio (SNR) between the filtered EEG signals (i.e., before artefact removal) and the artefact-free EEG signals. For each artefact type and testing dataset, the SNR was calculated for a representative channel, chosen as the channel that displayed the highest artefactual contamination in the filtered EEG. The position of the representative channel should be compatible with the artefact type. We did not calculate average SNR values across all channels because channels not affected by the artefact would mask the effectiveness of our denoising system. The representative channel differed from one testing dataset to another due to the type of artefact removed and to inter-individual differences. The reduction of cardiac contamination was estimated for a channel showing the highest SNR change between the filtered and the artefact-free EEG signals.

In the calculation of the SNR, by *signal* we mean the artefact and by *noise* we mean the EEG segment preceding the artefact. This definition implies that successful artefact removal from the EEG signal results in a reduction in the SNR (Urigüen & Garcia-Zapirain, 2015). For each dataset, the SNR was calculated on individual epochs defined with reference to the beginning of the artefact: the *noise* segment (n) includes 200 ms before the artefact, and the *signal* segment (s) follows the cue and has a length that varies across artefact types as a function of the inter-cue interval. For each segment (*noise* and *signal* separately), the average amplitude is subtracted from each timepoint in the segment. Then, the amplitude at each timepoint is squared, and the two maximum squared amplitude values (one for the *noise* segment—*max noise*^2^ and one for the *signal* segment—*max signal*^2^) are used to calculate the SNR for each subsequent *noise* and *signal* segments:

(15) \begin{eqnarray*}\mathrm{SNR}=10{\log \nolimits }_{10} \left( \frac{max signa{l}^{2}}{max nois{e}^{2}} \right) .\end{eqnarray*}

Finally, the SNR of the representative channel is calculated by averaging the SNR values obtained for all subsequent *noise* and *signal* segments.

## Results

Figure 3 shows the fingerprint, the topoplot (Space domain plot), the time course (Time domain plot), and the power spectrum (Frequency domain plot) of exemplary artefactual ICs, one for each artefact type. This overview refers to EEG datasets recorded with conventional wet electrodes.

### Performance of the SVM classifiers

The results of the statistical assessment of the SVM performance in classifying the four physiological artefacts in the two types of EEG recordings (wet and dry) and at the three decomposition levels (20, 50 and 80 ICs) are summarized in Fig. 4. For each artefact we report the results obtained for ten SVM classifiers and for the SVM classifier with the best performance. Details on the performance of each classifier are given in Tables S2 (eyeblinks), S3 (eye movements), S4 (myogenic artefacts) and S5 (cardiac interference). To correctly interpret these statistical results, it is important to bear in mind that the SVMs are trained to detect the artefactual ICs and not the individual artefacts present in the IC time course, and that an artefactual ICs generally contains multiple individual artefacts of the same type, such as multiple eyeblinks, multiple eye movements, etc. For these reasons, all artefacts of one type are included in few artefactual ICs, implying that there will generally be more non-artefactual ICs (ICs classified as “other”, TN) than artefactual ICs (TP).

*Eyeblinks:* The performance of the ten SVM classifiers for eyeblinks was very good, with each SVM obtaining an accuracy > 0.983, FOR < 0.017, HR > 0.714, FAR = 0, and *p* > 0.714 (Table S2). No substantial differences were observed in the performance of these classifiers with respect to electrode type or decomposition level. Five SVMs (SVM-3, SVM-4, SVM-5, SVM-6, and SVM-9) perfectly classified all ICs (see Table S2). Out of these classifiers, SVM-6 was considered the best because it was tested using a higher number of ICs with an equal number of wet and dry artefactual ICs, and achieved perfect classification also when 2 artefactual ICs were separated from the same dataset (see Table S2).

*Eye movements*: Good performances were obtained for the ten SVM classifiers trained to detect horizontal eye movements, with average accuracy > 0.94, FOR < 0.04, HR > 0.93, FAR < 0.06, and *p* > 0.93 (Fig. 4 and Table S3). No substantial differences were observed in the performance of these classifiers with respect to electrode type. Small differences in performance across classifiers were observed with respect to decomposition level, and no individual SVM achieved perfect performance across all conditions (see Table S3). SVM-2 is considered the best classifier because it achieved perfect classification for wet and dry EEG datasets decomposed in 50 ICs and for dry EEG datasets decomposed in 20 ICs. SVM-2 also achieved very good performance for both wet and dry EEG datasets decomposed at 80 ICs (*p* = 0.97) and for wet EEG datasets decomposed at 20 ICs (*p* = 0.95).

*Myogenic artefact*: The performance of the ten SVM classifiers for myogenic artefacts was also good, with an average accuracy > 0.93, FOR < 0.04, HR > 0.95, FAR < 0.18 and *p* > 0.94 (Fig. 4 and Table S4). Average accuracy showed no substantial differences with respect to electrode type; however, better values were obtained for EEG datasets decomposed at 50 and 80 ICs. On the other hand, the average sensitivity was equivalent across decomposition levels although slightly better for the wet datasets (see Table S4). No SVM achieved perfect performance for all conditions, although several classifiers had very good performance (see Table S4). SVM-8 is considered the best classifier because it achieved perfect classification for wet and dry EEG datasets decomposed at both 20 and 50 ICs and for dry EEG datasets decomposed at 80 ICs, with very good performance also for wet EEG datasets decomposed at 80 ICs (*p* > 0.98).

*Cardiac interference*: The performance of the ten SVM classifiers for cardiac interference was quite poor (Table S5). The average accuracy, FOR and FAR were quite good (mean accuracy > 0.93 with peak values > 0.99 for 80 ICs, mean FOR < 0.07 with peak values < 0.01 for 80 ICs, and mean FAR < 0.003 with zero values for wet EEG datasets decomposed at 20 and 80 ICs). However, average HR and *p* were very low: the best average HR values were obtained for wet EEG datasets decomposed at 80 ICs (HR = 0.320 ± 0.270), and the highest average values of *p* were achieved for wet EEG datasets decomposed at 80 ICs (*p* = 0.320 ± 0.270). No substantial differences were observed in the performance of these classifiers with respect to the type of electrode used for the EEG recordings or decomposition level, although the average performance across classifiers was better for decompositions at 80 ICs (see Table S5). As illustrated in Fig. 4 and Table S5, SVM-7 is the classifier with the overall best performance, with fairly good classification results on wet EEG datasets decomposed at 80 ICs (accuracy = 0.998, HR = 0.8, FAR = 0 and *p* = 0.8) and moderate classification results on dry EEG datasets decomposed at 20 ICs (accuracy = 0.97, HR = 0.667, FAR = 0.02 and *p* = 0.66) and wet EEG datasets decomposed at 50 ICs (accuracy = 0.984, HR = 0.6, FAR = 0.008 and *p* = 0.597). It is worth noting that, for two wet and three dry EEG datasets (i.e., eyeblink_dataset_wet_5; eye_movement_wet_dataset_2; eyeblink_dataset_dry_12; eye_movement_dry_dataset_2; eye_movement_dry_dataset_5), no IC containing cardiac interference was separated at 20 ICs decomposition level, although artefactual ICs were identified at higher decomposition levels. For this reason, for the SVM classifiers including these datasets in their testing group (i.e., for SVM-2, SVM-4, SVM-5, SVM-7, SVM-10) the number of artefactual ICs at the decomposition in 20 ICs was lower than the number of datasets.

**Figure 5:**
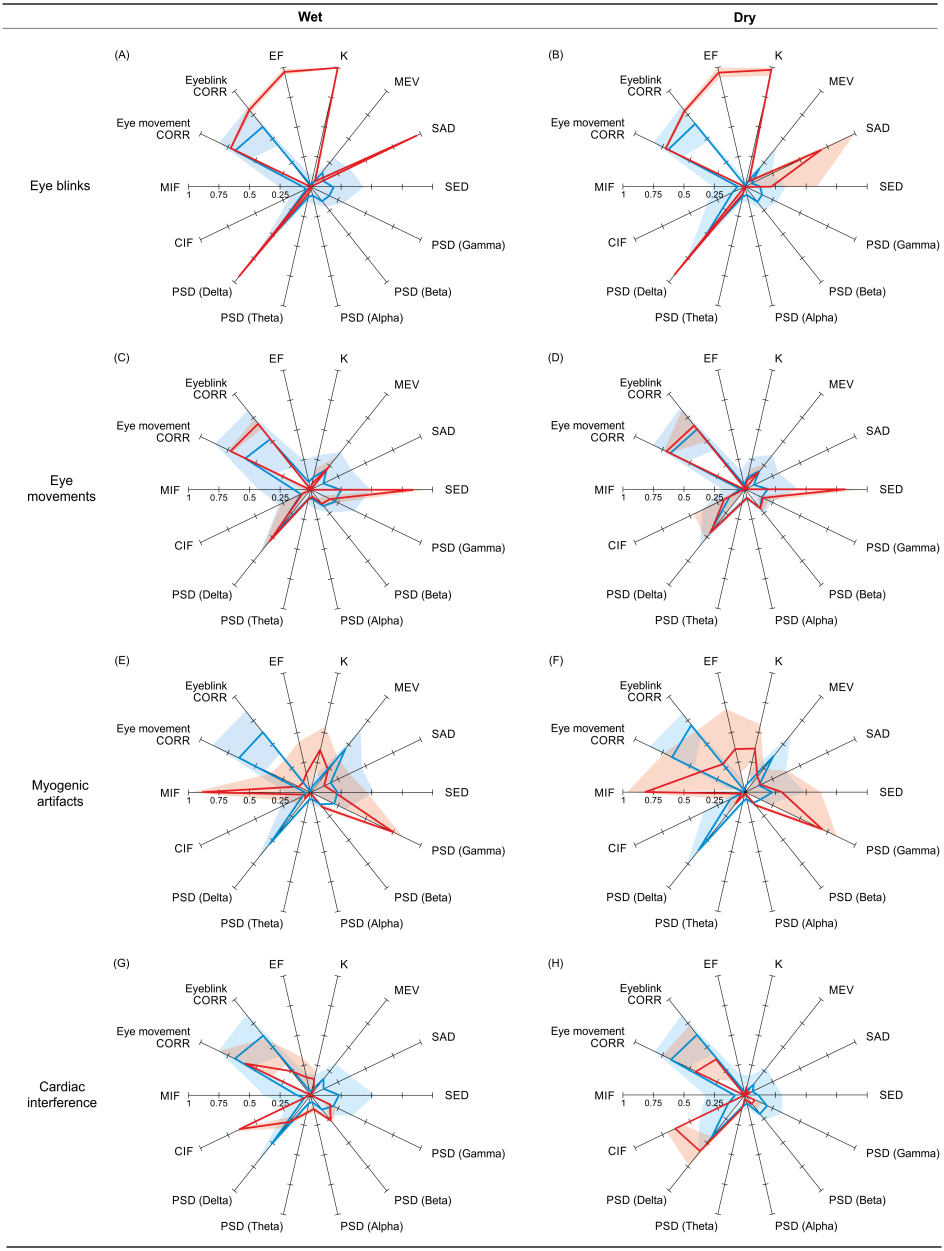
Reference and non-artifactual fingerprints for all artifacts. Overview of the reference and non-artifactual fingerprints obtained from the datasets used to train the best SVM classifier for each artifact type. The reference fingerprint (in red) and the non-artifactual fingerprint (in blue) are overlapped to permit easier comparison. The fingerprints obtained from wet EEG datasets are displayed in the panels (A, C, E, G); the fingerprints obtained from dry EEG datasets are displayed in the panels (B, D, F, H). In each fingerprint, the bold line connects the average values of the features, whereas the shaded areas represent the SD.

*All artefacts:* Figure 5 shows, for each artefact type, the reference and non-artefactual fingerprints obtained by averaging the fingerprints of the ICs (separately for wet and dry EEG datasets) used to train the SVM classifier with the best performance (see ‘Supervised SVM training’). For eyeblinks, eye movements and myogenic artefacts, the two reference fingerprints (wet and dry) are very similar and clearly different from the two non-artefactual fingerprints. Descriptive statistics on the individual features of the reference and non-artefactual fingerprints shown in Fig. 4 are provided in Tables S6 (eyeblinks), S7 (eye movements), S8 (myogenic artefacts) and S9 (cardiac interference).

The two reference fingerprints obtained for the detection of ICs containing cardiac interference (wet and dry) are less similar and only marginally distinguishable from the two non-artefactual fingerprints (wet and dry). CIF is the only feature that substantially differs between the reference and the non-artefactual fingerprints; however, this feature alone was insufficient to achieve good results.

### Quality assessment of the artefact-free EEG signals

**Figure 6:**
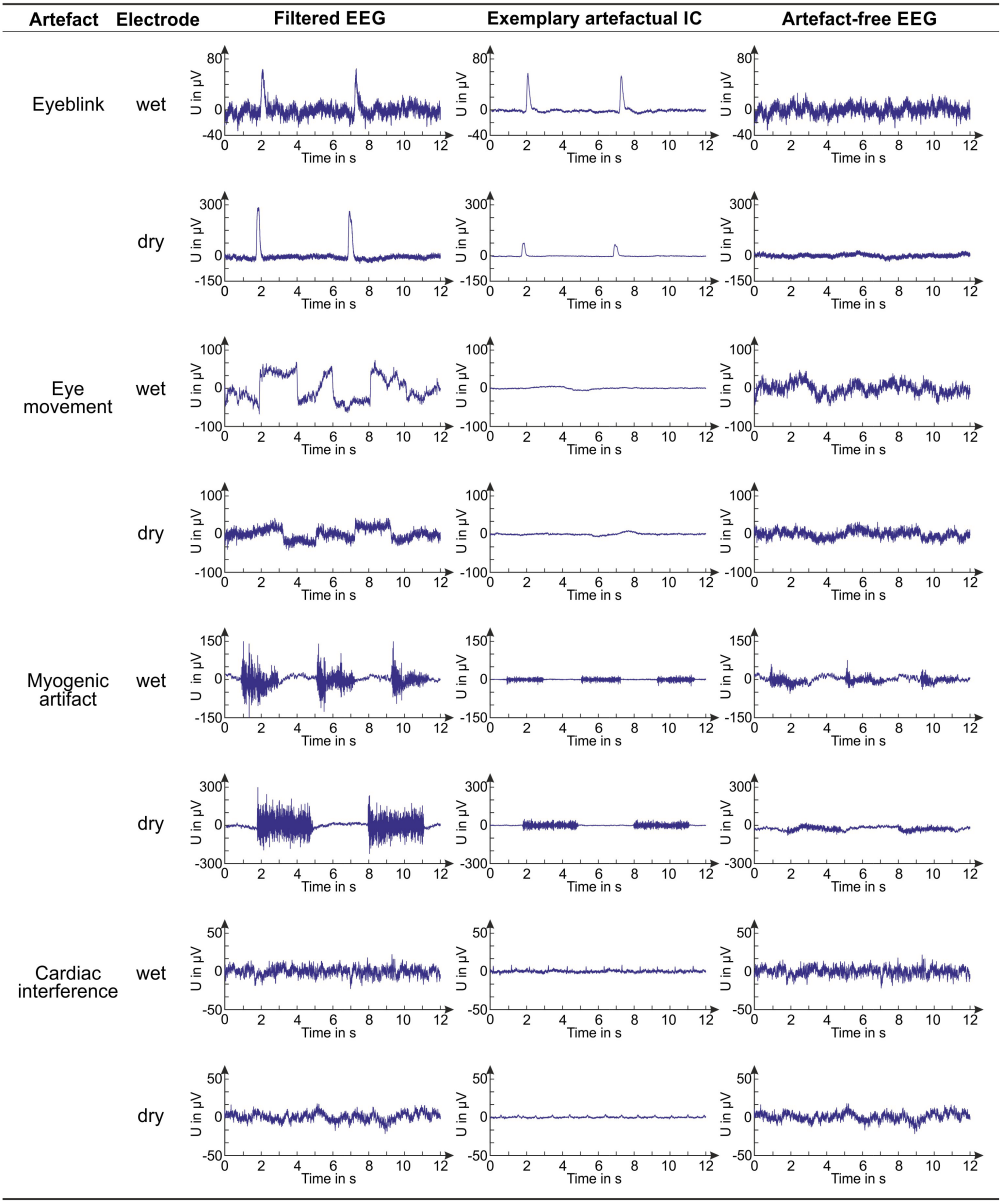
Examples of EEG signals before and after artifact removal. For each artifact and electrode type, we provide examples of a filtered EEG signal in a testing dataset, an artifactual IC (obtained from the same dataset, decomposition level: 80 ICs) and the artifact-free EEG signal corresponding to the shown filtered EEG signal. The artifact-free EEG signal is obtained after IC classification with the best SVM classifier.

The reduction of physiological interferences in the artefact-free EEG signals can be appreciated in Fig. 6, where, for each artefact, an example of a filtered EEG signal in the representative channel (see ‘Assessment of signal quality in artefact-free EEG’), an IC containing the artefact, and the artefact-free EEG signal in the representative channel are shown. Examples for both wet and dry EEG recordings are given. The contamination reduction in the artefact-free EEG signals is clear for all artefact types except cardiac interference which is generally not visible on the EEG recordings due to its small amplitude.

**Figure 7:**
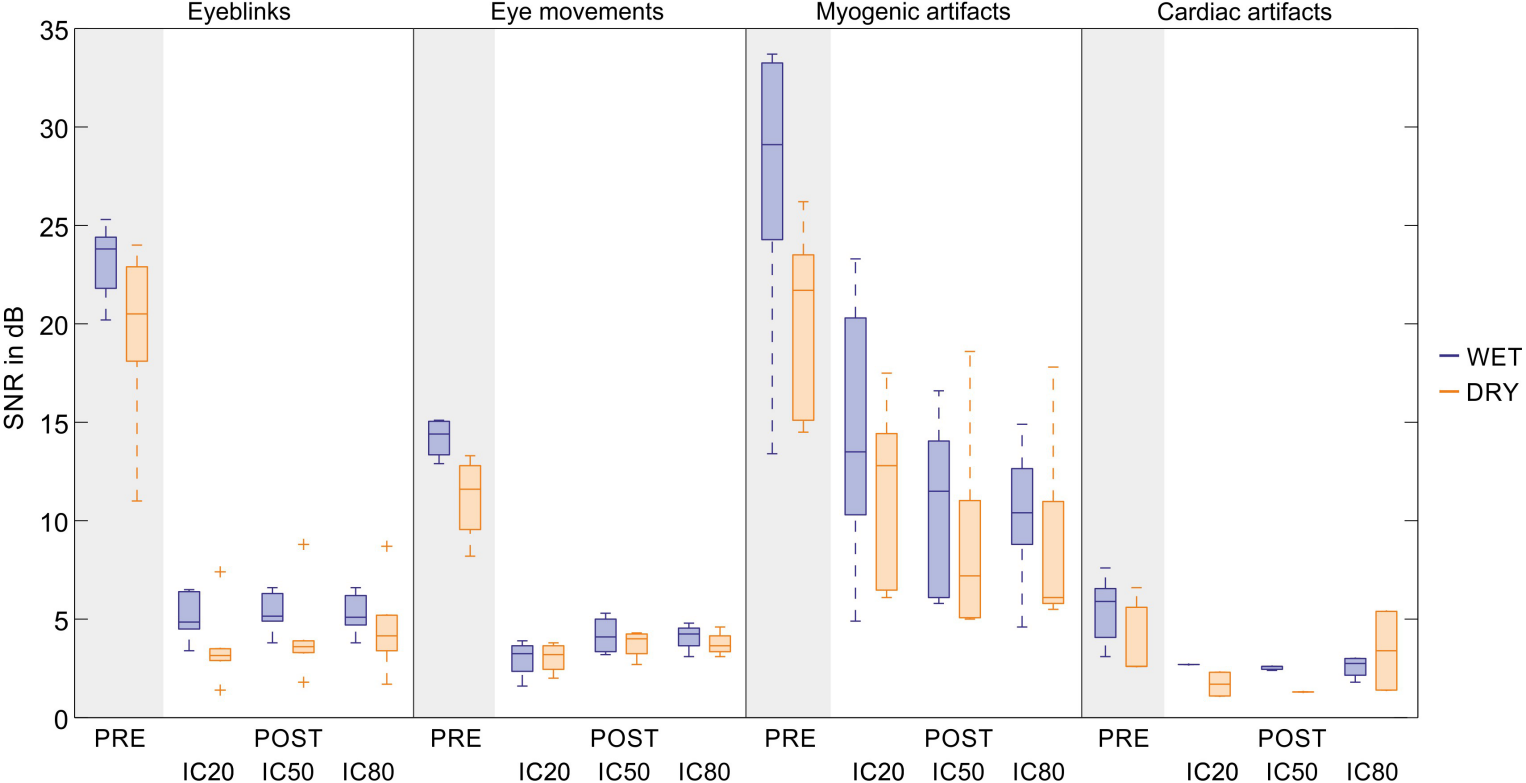
Descriptive statistics of the average SNR of EEG signals before and after artifact removal. For each artifact type, we report the descriptive statistics of the SNR values (in dB) of the filtered EEG signals (PRE) and of the artifact-free EEG signals (POST). The SNR values are calculated for the datasets used to test the SVM with the best performance. For each dataset, the SNR is calculated on the representative channel (details are given in Table S10, Panel 1). Results are given separately for wet and dry EEG datasets (color-coded) and for each decomposition level (20, 50 and 80 ICs). In the boxplots: the central line indicates the median; the box bottom/top indicate the 25th and 75th percentile respectively; the whiskers extend to the most extreme data points not considered outliers; the crosses indicate outliers. The average SNR values (Mean ± SD) and the average relative SNR reduction after artifact removal (in %, Mean ± SD) are provided for each artefact type in Table S10, Panel 2.

The interference reduction after artefact removal was quantified by the variation of the SNR (in dB). Figure 7 shows, for each artefact type, the average SNR reduction from the filtered EEG to the artefact-free EEG signals obtained by denoising the testing datasets with the best SVM. Results are shown separately for wet and dry EEG datasets and for all decomposition levels. Details on the SNR variations obtained for the individual testing datasets are given in Table S10 where the representative channel selected for each dataset is indicated (Panel 1) and the relative artefact reduction is also provided to give an immediate impression of the improvement in signal quality (Panel 2).

According to the SNR variations, signal quality is greatly improved for eyeblinks, eye movements and, to a lesser extent, myogenic artefacts. Very small SNR reductions are obtained for cardiac interference due to its very low amplitude. No substantial differences are observed between wet and dry EEG datasets or across decomposition levels.

The ranges of the average SNR in the artefact-free EEG signals (Table S10) are: 1.4–8.8 dB for eyeblinks, 1.6–5.3 dB for eye movements, 5.0–23.3 dB for myogenic artefacts, 1.1–5.4 dB for cardiac interference. The value of 23.3 dB was obtained for the dataset-9 in the group of wet EEG recordings containing cued myogenic artefacts. This value is an outlier and is due to the fact that, in the decomposition in 20 ICs, only one IC was non-artefactual which could be used to reconstruct the artefact-free EEG signal. Indeed, the SNR of the same channel when the dataset-9 was decomposed in 50 or 80 ICs was much lower (13.2 and 10.4 dB).

On average, the ranges of the relative artefact reduction in the artefact-free EEG signals are: 70%–88% for eyeblinks, 50%–84% for eye movements, 29%–79% for myogenic artefacts, and 6%–72% for cardiac artefacts. The highest and more consistent artefact reduction is attained for eyeblinks, which is expected because eyeblink artefacts have large amplitudes and a well defined waveform. Larger artefact reduction ranges are obtained for eye movements and myogenic artefacts because of greater amplitude variability. A broad range of relative artefact reduction is obtained for cardiac interference due its low amplitude and the failure of the SVM to correctly classify all artefactual ICs. The standard deviations could not be calculated for wet EEG recordings at 20 ICs and for dry EEG recordings at 50 ICs because only one IC was classified as artefactual in these conditions (see Fig. 7 and Table S10). Therefore, the SNR reduction after artefact removal appears quite small. It is also worth noting that the SNR values for the dry EEG recordings are generally smaller than those of the wet EEG recordings, because dry signals have, on average, a smaller signal amplitude.

## Discussion

The fingerprint method presented herein is a first release of an open architecture system based on a data-driven approach and on the use of specific artefact features that are meant to automatically detect the major physiological artefacts affecting EEG recordings.

### Performance of the SVM classifiers and quality of the artefact-free EEG signals

The performance of the SVM classifiers was generally very good for both wet and dry EEG recordings and for all the analyzed physiological artefacts except cardiac interference. Consistency in the performance of all 10 SVM classifiers trained and tested on diverse groups of datasets for each artefact suggests that the method is both reliable and robust.

*Eyeblinks:* The best classification results were obtained for eyeblinks, for which half of the trained SVMs perfectly classified all ICs, and the performances of the remaining SVM classifiers were strong (Table S2). Automatic IC classification was comparable for both wet and dry EEG recordings, and independent from the number of ICs in which the EEG dataset was decomposed. This outcome implies that our method can be effectively used to remove eyeblinks from diverse EEG datasets recorded using different types and number of electrodes, from the typical 21 wet electrode arrays used for clinical purposes to the high density EEG caps (wet and dry) used in research applications. This excellent outcome can be ascribed to the good definition of the reference fingerprints and their clear dissimilarity from the non-artefactual fingerprints for both wet and dry EEG datasets (see Fig. 5). Compared to other methods developed to detect eyeblinks, which have achieved accuracies ranging from 0.97 to 0.99 (e.g., Viola et al., 2009; Mognon et al., 2011; Frølich, Andersen & Mørup, 2015; Chang, Lim & Im, 2016), our fingerprint method shows superior performance and achieved perfect classification in half of the trained SVMs, although a direct comparison of methods might highlight different performances due to the type of datasets used (cued-artefact datasets vs. no-cued real datasets) or to the type of the trained classifier (binary vs. multi-class). However, although the quality of the artefact-free EEG signals is much improved, some residual eyeblink contamination is still present (average SNR of about 4–5 dB, i.e., of the order of 1/5 of its value in the filtered EEG, with differences across datasets). This fact is not due to the SVM performance but rather to the EEG decomposition. Some ICs not classified as artefactual by both the experts and the SVM classifiers may still contain some residual eyeblinks not visible in the IC time course nor automatically detectable. Given that the artefact-free EEG signals are reconstructed by re-projecting the non-artefactual ICs into sensor space, this residual contamination could have been included in the EEG signals after artefact removal.

Preserving brain activity in its entirety must be the final goal of any method for artefact removal. From our results it seems that, although our classification method had perfect performance, ICA failed to correctly separate all artefactual sources from true brain activity. Recently, new ICA-based methods, such as the Enhanced Wavelet ICA (Castellanos & Makarov, 2006; Mammone, La Foresta & Morabito, 2012), have been introduced to minimize information loss in the reconstructed brain signals after removal of artefactual ICs. Improvements of our method might include the use of this new approach to advance separation between artefactual sources and true brain activity and achieve better results in the reconstruction of true brain signals.

*Eye movements:* Very good classification results were also achieved for the detection of horizontal eye movements. Although no SVM achieved perfect classification for all decomposition levels and electrode types (Table S3), the best SVM (SVM-2) was still able to perfectly classify all ICs at the decomposition level of 50 ICs for both wet and dry datasets. Only Frølich and colleagues (2015) have achieved perfect classification of horizontal eye movements. Mognon and colleagues (2011) obtained an accuracy of 0.992, and Viola and colleagues (2009) attained correlations between the automatic classifiers and the human classifiers ranging from 0.85 to 0.91 as a function of the density of the EEG cap. However, in the case of accuracy values < 0.90, estimates of the method sensitivity and consistency based on the Hit Rate and False Alarm Rate could indicate lower performances than accuracy alone. Nevertheless, as for eyeblinks, a direct comparison of methods might highlight different performances due to the type of datasets used, type of trained classifier, or manual labeling of the artefactual ICs.

It is worth mentioning that the classification results obtained for horizontal eye movements were affected by a relatively high number of false positives (FP), especially for decompositions in 50 and 80 ICs. The FP mainly referred to ICs containing vertical or mixed eye movements, which were classified as “other” by our experts because their time course differs from the one of horizontal eye movements. However, the PSD content (and therefore the PSD-based features) and the features SAD and SED, designed to differentiate between eyeblinks and eye movements, are independent from the direction of movement. Also, the highly similar values of the CORR features in the reference and non-artefactual fingerprints (see Fig. 5 and Table S7) may indicate that the non-artefactual fingerprints were built from the fingerprints of ICs classified as “other” but containing vertical or mixed eye movements. These facts could explain the high number of FP in the classification. Therefore, we think that the performance of the SVM classifiers for eye movements could be improved by extending the classification to vertical and mixed eye movements, i.e., by including the ICs containing these movements in the group of the artefactual ICs during the training of the SVM classifiers.

The good classification obtained with SVM-2 is confirmed by the quality improvement in the reconstructed artefact-free EEG signals, for which the SNR was reduced, on average, to 1/4 of its value in the filtered EEG signals, and the residual eye movement contamination (on the order of 3–4 dB) is likely due to an ineffective EEG decomposition, as for eyeblinks.

*Myogenic artefacts:* Very good classification results were also obtained for myogenic artefacts. Although no SVM achieved perfect classification for all decomposition levels and types of electrodes (Table S4), the best SVM (i.e., SVM-8) perfectly classified all ICs when the EEG datasets (both wet and dry) were decomposed in 20 and 50 ICs, and when the dry EEG datasets were decomposed in 80 ICs. SVM-8 also achieved extremely good performance on wet EEG datasets decomposed in 80 ICs, with an accuracy of 0.96 and a sensitivity, *p* of 0.98. This means that our method can effectively remove myogenic artefacts from EEG datasets recorded with electrode arrays of various density and type. Given these good classification results, we might say that our fingerprint method outperformed other methods, such as the one introduced by Frølich and colleagues (2015), which achieved an accuracy of 0.87, or the automated artefact elimination method proposed by Radüntz and colleagues (2015) which achieved an accuracy of 0.88, although differences might arise for a direct comparison of the methods. The fingerprint method is therefore a viable alternative to existing methods to remove myogenic interference from EEG signals recorded in both clinical and research settings.

Notwithstanding the good classification performance of SVM-8, the SNR of the artefact-free EEG signals is still quite high across all decomposition levels (average values ranging from 9 dB to 14 dB), indicating that myogenic artefacts were not completely removed. As for eyeblinks and horizontal eye movements, this outcome might be related to an ineffective signal decomposition with ICA.

*Cardiac interference:* In contrast to the classification of the other artefact types, poor classification results were obtained for cardiac interference (Table S5), and no SVM achieved perfect classification. The accuracy of the SVM with the best performance (i.e., SVM-7) was good (always >0.97), but the sensitivity, *p* was generally low (ranging from 0.193 to 0.800), as a consequence of the low Hit Rate values (ranging from 0.2 to 0.8). These results were due to several misclassifications, and could be ascribed to the inherent difficulty in characterizing cardiac interference due to its low amplitude, which makes it difficult to differentiate from other EEG sources. Indeed, the wet and dry reference fingerprints were dissimilar and not well-defined. This is partly due to a difference in the frequency content of the wet and dry ICs. PSD content in the wet ICs is evenly distributed across all frequency bands, whereas in the dry ICs it is prevalent in the Delta band. Also, the CORR features (especially CORR eye movement) have a wide range of values in both the wet and dry reference fingerprints. Although the cardiac depolarization is a much shorter event than eyeblinks or eye movements, the rapid amplitude changes occurring in the IC time course at the beginning of cardiac interference events could have been detected as the initial phases of eyeblinks or eye movements, leading to high CORR values. Finally, CIF is the only feature that greatly differs between the reference and the non-artefactual fingerprints. However, in a multivariate approach like the fingerprint method this is clearly insufficient to achieve good classification results. These problems might be overcome if simultaneous ECG was recorded. This procedure could help define a cardiac template and/or new features based on the PSD content of the cardiac activity that might improve the definition of the reference fingerprint and therefore the classification results. Better results could also be achieved by combining the improvements in the definition of the cardiac reference fingerprint with more effective methods to decompose the EEG signals.

Other methods have had variable performances in detecting cardiac interference. The method introduced Frølich and colleagues (2015) achieved accuracy equal to 1, but the approach proposed by Viola and colleagues (2009) attained correlations between the automatic classifiers and the human classifiers that ranged from 0.62 to 0.71, but only for high density EEG caps. Therefore, given that no direct information on the cardiac activity could be used for the training of the SVMs, the classification results that we obtained are encouraging. However, the improvement of signal quality in the artefact-free EEG signals was very low, with an extremely variable relative artefact reduction, ranging from 6% to 72% (Fig. 7 and Table S10). This outcome depends on two factors: the low amplitude of the cardiac interference, and the poor classification performance. In fact, for some testing datasets the artefact-free EEG signals could not be reconstructed because no artefactual IC was detected even by the best classifier (SVM-7, see Table S10).

### Decomposition of the EEG recordings

The classification results obtained for the eyeblinks, eye movements and myogenic artefacts suggest that the best performance was achieved for 50 and 20 ICs. In general, the most appropriate number of ICs in which the EEG datasets should be decomposed is related to the nature of the artefactual signal being detected. For instance, very well-defined artefacts like eyeblinks generally do not require a high decomposition level, whereas a higher number of ICs would be preferable for artefacts with less well-defined waveforms and lower amplitudes, such as cardiac interference artefacts. In general, the use of a lower decomposition level (such as 20 or 50 ICs) would ensure the avoidance of problems related to signal over-decomposition.

Another advantage of using low decomposition levels is related to computational load. In our study, we used EEG recordings of limited duration, therefore the computational load for the application of ICA to the entire pre-processed EEG recordings was not very high. However, other potential applications of our method, where the duration of the EEG recordings (and thus of the ICs) may be longer, will demand a greater computational load required to calculate the IC fingerprints. For this reason, a lower number of separated ICs would be preferable. A lower decomposition level would also be preferable in potential online or real-time applications of our method.

A problem that may be encountered when decomposing long EEG recordings is signal stationarity (Comon, 1994; Bell & Sejnowski, 1995; Murta Jr et al., 2015). An improved version of our method might include ICA applied to windowed EEG segments. This approach would overcome potential violations of the stationarity assumption in longer datasets and would lead to greater independence of sources. Given that applying ICA to windowed EEG segments implies longer computation times; the overall efficiency of the method would increase if lower decomposition levels were used.

Finally, it has already been mentioned that a more efficient separation between artefacts and true brain activity signals can be achieved using advanced signal decomposition methods such as the Enhanced Wavelet ICA (Castellanos & Makarov, 2006; Mammone, La Foresta & Morabito, 2012). Future developments of our method might consider the use of this method for EEG signal decomposition.

### Computational load

The computational efficiency of each SVM classifier was estimated in terms of average computational time, calculated on a computer with an Intel Xeon W3520 2.67 GHz processor with 6 GB RAM, while running a 64 bit Windows 7 Professional operating system. All other processes were deactivated. Computational time was on the order of 10 ms, and did not depend on type of artefact, number of separated ICs, or IC length. The preparation of the matrices required by the SVM classification required approximately 130–160 ms.

However, the computational time required to calculate the fingerprint features for all the ICs of one dataset was on the order of several seconds and depended on the type of artefact (shortest times for eyeblinks, longest times for cardiac interferences), and on the number of ICs and dataset length. The computation time differences due to the number of ICs are substantial: for a dataset of 5 min, the average computation time is 1.39 ± 0.07 min for 20 ICs, 3.35 ± 0.30 min for 50 ICs, and 5.41 ± 0.34 min for 80 ICs. These long computation times are due to the calculation of some specific fingerprint features: The correlation features (CORR eyeblink and CORR eye movement) account for the 88% of the total computation time (44% each feature), MEV and CIF account for the 4% each (8% in total), and K accounts for 3%. Therefore, these five features alone account for 99% of the total computation time of a fingerprint. From the perspective of the implementation of an online version of our method, it would be preferable to decompose the original EEG recordings in a low number of ICs.

## Conclusions

The validation of the fingerprint method using real EEG datasets containing cued physiological artefacts shows that the method performed effectively in classifying eyeblink, eye movement and myogenic artefact ICs. Perfect classification was obtained at all IC decomposition levels when detecting eyeblinks and myogenic artefacts, and for EEG datasets decomposed in 20 and 50 ICs when detecting eye movements. The residual physiological contamination in the artefact-free EEG signals indicates that signal decomposition should be performed with advanced ICA methods capable of minimizing information loss in the reconstructed brain signals. However, the consistently decreasing SNR after artefact removal confirms the effectiveness of the fingerprint method which achieved equal or superior performance compared to existing methods for the automatic removal of eyeblinks, eye movements and myogenic artefacts. On the other hand, the poor classification performance obtained for cardiac interference indicates that new features should be investigated to better define the reference fingerprints.

We demonstrated that our fingerprint method does not depend upon the number of electrodes used or the specific electrode layout or type (wet or dry). Our method also has additional advantages because it does not require simultaneous recording of artefactual reference signals (such as EOG and EMG), it classifies artefacts using features that are independent and specific, and it employs a nonlinear SVM approach for improved ICs classification.

Our findings suggest several potential improvements. For example, the decomposition of the training datasets could be restricted to a low number of ICs. Additionally, applying ICA to windowed EEG segments would allow application of our method to long EEG recordings and enable online processing without violating stationarity assumptions. A more effective subset of features, specifically tailored to each type of artefact, may also improve the method and broaden its applicability. Given the open architecture of our system, the independence and specificity of the fingerprint features permits straight-forward training of new SVMs based on different numbers and types of features. These enhancements will contribute to optimized artefact detection and enhanced signal quality which preserves brain activity in the artefact-free EEG signals, leading to improved efficiency and wider applicability of the system. Further developments may also include further validation of our method in a variety of EEG recordings, such as EEG datasets acquired during neurophysiological studies, and an optimization procedure to improve generalizability and cost-effectiveness, with the ultimate aim of implementing a method with straightforward setup and usability.

##  Supplemental Information

10.7717/peerj.4380/supp-1 Figure S1 Layout of the caps used for EEG acquisitions Layout of the caps used for EEG acquisitions: (A) the novel dry electrode cap with 97 dry Multipin Polyurethane electrodes with an Ag/AgCl coating, arranged in an equidistant layout; (B) the commercial wet cap (Waveguard, Advanced Neuro Technologies B.V., Enschede, Netherlands) with 128 Ag/AgCl electrodes in a quasi-equidistant layout.

10.7717/peerj.4380/supp-2 Table S1 Groups of training and testing datasets Composition of the groups of EEG datasets used to train and test each SVM classifier for each artifact. Training and testing groups include wet and dry EEG datasets. Each dataset is identified only by the number. The type of artifact and electrode can be inferred from the position in the table. Datasets used for classifying ICs containing cardiac interference include the acronym of the cued artifact (i.e., EB for eyeblinks and EM for eye movements).

10.7717/peerj.4380/supp-3 Table S2 Statistical performance of the ten SVMs trained to classify eyeblinks The statistical performance of the individual SVM classifiers trained to classify ICs containing eyeblinks is reported separately for the wet and dry testing EEG datasets and for the three decomposition levels (20, 50 and 80 ICs).

10.7717/peerj.4380/supp-4 Table S3 Statistical performance of the ten SVMs trained to classify eye movements The statistical performance of the individual SVM classifiers trained to classify ICs containing eye movements is reported separately for the wet and dry testing EEG datasets and for the three decomposition levels (20, 50 and 80 ICs).

10.7717/peerj.4380/supp-5 Table S4 Statistical performance of the ten SVMs trained to classify myogenic artifacts The statistical performance of the individual SVM classifiers trained to classify ICs containing myogenic artifacts is reported separately for the wet and dry testing EEG datasets and for the three decomposition levels (20, 50 and 80 ICs).

10.7717/peerj.4380/supp-6 Table S5 Statistical performance of the ten SVMs trained to classify cardiac interference The statistical performance of the individual SVM classifiers trained to classify ICs containing cardiac interference is reported separately for the wet and dry testing EEG datasets and for the three decomposition levels (20, 50 and 80 ICs).

10.7717/peerj.4380/supp-7 Table S6 Descriptive statistics of individual features in eyeblink fingerprints Descriptive statistics of the individual features of the reference and non-artifactual fingerprints for eyeblinks are given separately for wet and dry EEG datasets.

10.7717/peerj.4380/supp-8 Table S7 Descriptive statistics of individual features in eye movement fingerprints Descriptive statistics of the individual features of the reference and non-artifactual fingerprints for eye movements are given separately for wet and dry EEG datasets.

10.7717/peerj.4380/supp-9 Table S8 Descriptive statistics of individual features in myogenic artifact fingerprints Descriptive statistics of the individual features of the reference and non-artifactual fingerprints for myogenic artifacts are given separately for wet and dry EEG datasets.

10.7717/peerj.4380/supp-10 Table S9 Descriptive statistics of individual features in cardiac interference fingerprints Descriptive statistics of the individual features of the reference and non-artifactual fingerprints for cardiac interference are given separately for wet and dry EEG datasets.

10.7717/peerj.4380/supp-11 Table S10 SNR values of individual EEG datasets before and after artifact removal **Panel 1:** For each artifact type, we report the average SNR values of the filtered EEG signals and of the artifact-free EEG signals for the individual datasets used to test the SVM classifier with the best performance. For each dataset, the SNR is calculated on the representative channel, that can vary across datasets. Results are given separately for wet and dry EEG datasets and for each decomposition level (20, 50 and 80 ICs). SNR values are given in dB (Mean ± SD). **Panel 2:** For each artifact type, we report the average SNR values of the filtered EEG signals and of the artifact-free EEG signals separately for wet and dry EEG datasets and for each decomposition level (20, 50 and 80 ICs). SNR values are given in dB (Mean ± SD). The average relative SNR reduction after artifact removal is given in % (Mean ± SD).

## Additional Information and Declarations

### Data Availability

The following information was supplied regarding data availability: The raw EEG data with cued artifacts have been uploaded to FigShare: DOI: 10.6084/m9.figshare.5537338
DOI: 10.6084/m9.figshare.5537326
DOI: 10.6084/m9.figshare.5537323
DOI: 10.6084/m9.figshare.5537314
DOI: 10.6084/m9.figshare.5537293
DOI: 10.6084/m9.figshare.5537275.

## References

[ref-1] Aizerman MA, Braverman EM, Rozonoer LI (1964). Theoretical foundations of the potential function method in pattern recognition learning. Automation and Remote Control.

[ref-2] Askamp J, Van Putten MJ (2014). Mobile EEG in epilepsy. International Journal of Psychophysiology.

[ref-3] Barbati G, Porcaro C, Zappasodi F, Rossini PM, Tecchio F (2004). Optimization of an independent component analysis approach for artefact identification and removal in magnetoencephalographic signals. Clinical Neurophysiology.

[ref-4] Bell AJ, Sejnowski TJ (1995). An information-maximization approach to blind separation and blind deconvolution. Neural Computation.

[ref-5] Bishop CB (2006). Pattern recognition and machine learning.

[ref-6] Burges CJC (1998). A tutorial on support vector machines for pattern recognition. Data Mining and Knowledge Discovery.

[ref-7] Castellanos NP, Makarov VA (2006). Recovering EEG brain signals: artefact suppression with wavelet enhanced independent component analysis. Journal of Neuroscience Methods.

[ref-8] Cawley GC, Talbot NLC (2010). On over-fitting in model selection and subsequent selection bias in performance evaluation. The Journal of Machine Learning Research.

[ref-9] Chang WD, Lim JH, Im CH (2016). An unsupervised eye blink artefact detection method for real-time electroencephalogram processing. Physiological Measurement.

[ref-10] Chaumon M, Bishop DVM, Busch NA (2015). A practical guide to the selection of independent components of the electroencephalogram for artefact correction. Journal of Neuroscience Methods.

[ref-11] Comani S, Liberati M, Mantini D, Gabriele E, Brisinda D, Di Luzio S, Fenici R, Romani GL (2004a). Characterization of fetal arrhythmias by means of fetal magnetocardiography in three cases of difficult ultrasonographic imaging. Pacing and Clinical Electrophysiology.

[ref-12] Comani S, Mantini D, Lagatta A, Esposito F, Di Luzio S, Romani GL (2004b). Time course reconstruction of fetal cardiac signals from fMCG: independent component analysis versus adaptive maternal beat subtraction. Physiological Measurement.

[ref-13] Comani S, Velluto L, Schinaia L, Cerroni G, Serio A, Buzzelli S, Sorbi S, Guarnieri B (2015). Monitoring neuro-motor recovery from stroke with high-resolution EEG, robotics and virtual reality: a proof of concept. IEEE Transactions on Neural System and Rehabilitation Engineering.

[ref-14] Comon P (1994). Independent component analysis, a new concept. Signal Processing.

[ref-15] Croft RJ, Barry RJ (2000). EOG correction of blinks with saccade coefficients: a test and revision of the aligned-artefact average solution. Clinical Neurophysiology.

[ref-16] Croft RJ, Barry RJ (2002). Issues relating to the subtraction phase in EOG artefact correction of the EEG. International Journal of Psychophysiology.

[ref-17] Daly I, Scherer R, Billinger M, Müller-Putz G (2015). FORCe: fully online and automated artefact removal for brain-computer interfacing. IEEE Transactions on Nueral Systems and Rehabilitation Engineering.

[ref-18] De Martino F, Gentile F, Esposito F, Balsi M, Di Salle F, Goebel R, Formisano E (2007). Classification of fMRI independent components using IC-fingerprints and support vector machine classifiers. NeuroImage.

[ref-19] De Vos M, Kroesen M, Emkes R, Debener S (2014). P300 speller BCI with a mobile EEG system: comparison to a traditional amplifier. Journal of Neural Engineering.

[ref-20] Del Percio C, Iacoboni M, Lizio R, Marzano N, Infarinato F, Vecchio F, Bertollo M, Robazza C, Comani S, Limatola C, Babiloni C (2011). Functional coupling of parietal alpha rhythms is enhanced in athletes before visuomotor performance: a coherence electroencephalographic study. Neuroscience.

[ref-21] Delorme A, Makeig S (2004). EEGLAB: an open source toolbox for analysis of single-trial EEG dynamics including independent component analysis. Journal of Neuroscience Methods.

[ref-22] Delorme A, Palmer J, Onton J, Oostenveld R, Makeig S (2012). Independent EEG sources are dipolar. PLOS ONE.

[ref-23] Delorme A, Sejnowski T, Makeig S (2007). Enhanced detection of artefacts in EEG data using higher-order statistics and independent component analysis. Neuroimage.

[ref-24] Delorme A, Westerfield M, Makeig S (2007). Medial prefrontal theta bursts precede rapid motor responses during visual selective attention. The Journal of Neuroscience.

[ref-25] Di Fronso S, Robazza C, Filho E, Bortoli L, Comani S, Bertollo M (2016). Neural markers of performance states in an Olympic Athlete: an EEG case study in air-pistol shooting. Journal of Sport Science and Medicine.

[ref-26] Fiedler P, Pedrosa P, Griebel S, Fonseca C, Vaz F, Supriyanto E, Zanow F, Haueisen J (2015). Novel multipin electrode cap system for dry electroencephalography. Brain Topography.

[ref-27] Filho E, Bertollo M, Tamburro G, Schinaia L, Chatel-Goldman J, Di Fronso S, Robazza C, Comani S (2016). Hyperbrain features of team mental models within a juggling paradigm: a proof of concept. PeerJ.

[ref-28] Freeman WJ, Holmes MD, Burke BC, Vanhatalo S (2003). Spatial spectra of scalp EEG and EMG from awake humans. Clinical Neurophysiology.

[ref-29] Friedman BH, Thayer JF (1991). Facial muscle activity and EEG recordings: redundancy analysis. Electroencephalography and Clinical Neurophysiology.

[ref-30] Frølich L, Andersen TS, Mørup M (2015). Classification of independent components of EEG into multiple artefact classes. Psychophysiology.

[ref-31] Goncharova II, McFarland DJ, Vaughan TM, Wolpaw JR (2003). EMG contamination of EEG: spectral and topographical characteristics. Clinical Neurophysiology.

[ref-32] Greco A, Mammone N, Morabito FC, Versaci M (2006). Kurtosis, Renyi’s entropy and independent component scalp maps for the automatic artefact rejection from EEG data. International Journal of Signal Processing.

[ref-33] Halder S, Bensch M, Mellinger J, Bogdan M, Kubler A, Birbaumer N, Rosenstiel W (2007). Online artefact removal for brain-computer interfaces using support vector machines and blind source separation. Computational Intelligence and Neuroscience.

[ref-34] Hou J, Morgan K, Tucker DM, Konyn A, Poulsen C, Tanaka Y, Anderson EW, Luu P (2016). An improved artefacts removal method for high dimensional EEG. Journal of Neuroscience Methods.

[ref-35] Jin C, Wang L (2012). Dimensionality dependent PAC-Bayes margin bound.

[ref-36] Jordan KG (2004). Emergency EEG and continuous EEG monitoring in acute ischemic stroke. Journal of Clinical Neurophysiology.

[ref-37] Jung T, Makeig S, Humphries C, Lee T, Mckeown M, Iragui V, Sejnowski TJ (2000). Removing electroencephalographic artefacts by blind source separation. Psychophysiology.

[ref-38] Karhunen J, Oja E, Wang L, Vigario R, Joutsensalo J (1997). A class of neural networks for independent component analysis. IEEE Transactions on Neural Networks.

[ref-39] Kilicarslan A, Grossman RG, Contreras-Vidal JL (2016). A robust adaptive denoising framework for real-time artifact removal in scalp EEG measurements. Journal of Neural Engineering.

[ref-40] Lance BJ, Kerick SE, Ries AJ, Oie KS, McDowell K (2012). Brain-computer interface technologies in the coming decades. Proceedings of the IEEE.

[ref-41] Lee TW, Girolami M, Bell AJ, Sejnowski TJ (2000). A unifying information-theoretic framework for independent component analysis. Computers and Mathematics with Applications.

[ref-42] Lee TW, Girolami M, Sejnowski TJ (1999). Independent component analysis using an extended infomax algorithm for mixed subgaussian and supergaussian sources. Neural Computation.

[ref-43] LeVan P, Urrestarazu E, Gotman J (2006). A system for automatic artefact removal in ictal scalp EEG based on independent component analysis and Bayesian classification. Clinical Neurophysiology.

[ref-44] Liao LD, Wu SL, Liou CH, Lu SW, Chen SA, Chen SF, Ko LW, Lin CT (2014). A novel 16-channel wireless system for electroencephalography measurements with dry spring-loaded sensors. IEEE Transactions on Instrumentation and Measurement.

[ref-45] Lopez-Gordo MA, Sanchez-Morillo D, Pelayo Valle F (2014). Dry EEG electrodes. Sensors.

[ref-46] Mammone N, La Foresta F, Morabito FC (2012). Automatic artefact rejection from multichannel scalp EEG by wavelet ICA. IEEE Sensors Journal.

[ref-47] Mantini D, Franciotti R, Romani GL, Pizzella V (2008). Improving MEG source localizations: an automated method for complete artefact removal based on independent component analysis. Neuroimage.

[ref-48] McMenamin BW, Shackman AJ, Maxwell JS, Bachhuber DR, Koppenhaver AM, Greischar LL, Davidson RJ (2010). Validation of ICA-based myogenic artefact correction for scalp and source-localized EEG. Neuroimage.

[ref-49] Michel V, Mazzola L, Lemesle M, Vercueil L (2015). Long-term EEG in adults: sleep-deprived EEG (SDE), ambulatory EEG (Amb-EEG) and long-term video-EEG recording (LTVER). Clinical Neurophysiology.

[ref-50] Mognon A, Jovicich J, Bruzzone L, Buiatti M (2011). ADJUST: an automatic EEG artefact detector based on the joint use of spatial and temporal features. Psychophysiology.

[ref-51] Mullen TR, Kothe CA, Chi YM, Ojeda A, Kerth T, Makeig S, Jung TP, Cauwenberghs G (2015). Real-time neuroimaging and cognitive monitoring using wearable dry EEG. IEEE Transactions on Biomedical Engineering.

[ref-52] Murta Jr LO, Guzo MG, Moraes ER, Baffa O, Wakai RT, Comani S (2015). Segmented independent component analysis for improved separation of fetal cardiac signals from non-stationary fetal magnetocardiograms. Biomedical Engineering/Biomedizinische Technik.

[ref-53] National Research Council (US) Committee on Vision (1985). Appendix b: detection sensitivity and response bias. Emergent techniques for assessment of visual performance.

[ref-54] Niedermeyer E, Da Silva FHL (2005). Electroencephalography: basic principles, clinical applications, and related fields.

[ref-55] Nolan H, Whelan R, Reilly RB (2010). FASTER: fully automated statistical thresholding for EEG artefact Rejection. Journal of Neuroscience Methods.

[ref-56] Onton J, Westerfield M, Townsend J, Makeig S (2006). Imaging human EEG dynamics using independent component analysis. Neuroscience and Biobehavioral Reviews.

[ref-57] Pincus SM (1991). Approximate entropy as a measure of system complexity. Proceedings of the National Academy of Sciences of the United States of America.

[ref-58] Radüntz T, Scouten J, Hochmuth O, Meffert B (2015). EEG artefact elimination by extraction of ICA-component features using image processing algorithms. Journal of Neuroscience Methods.

[ref-59] Radüntz T, Scouten J, Hochmuth O, Meffert B (2017). Automated EEG artifact elimination by applying machine learning algorithms to ICA-based features. Journal of Neural Engineering.

[ref-60] Thompson T, Steffert T, Ros T, Leach J, Gruzelier J (2008). EEG applications for sport and performance. Methods.

[ref-61] Urigüen JA, Garcia-Zapirain B (2015). EEG artifact removal—state-of-the-art and guidelines. Journal of Neural Engineering.

[ref-62] Vapnik V (1995). The nature of statistical learning theory.

[ref-63] Vapnik V (1998). Statistical learning theory.

[ref-64] Vigário R, Särelä J, Jousmäki V, Hämäläinen M, Oja E (2000). Independent component approach to the analysis of EEG and MEG recordings. IEEE Transactions on Biomedical Engineering.

[ref-65] Viola FC, Thorne J, Edmonds B, Schneider T, Eichele T, Debener S (2009). Semi-automatic identification of independent components representing EEG artefact. Clinical Neurophysiology.

[ref-66] Vorobyov S, Cichocki A (2002). Blind noise reduction for multisensory signals using ICA and subspace filtering, with application to EEG analysis. Biological Cybernetics.

[ref-67] Welch P (1967). The use of fast Fourier transform for the estimation of power spectra: a method based on time averaging over short, modified periodograms. IEEE Transactions on Audio and Electroacoustics.

[ref-68] Whitham EM, Pope KJ, Fitzgibbon SP, Lewis T, Clark CR, Loveless S, Broberg M, Wallace A, DeLosAngeles D, Lillie P, Hardy A, Fronsko R, Pulbrook A, Willoughby JO (2007). Scalp electrical recording during paralysis: quantitative evidence that EEG frequencies above 20 Hz are contaminated by EMG. Clinical Neurophysiology.

[ref-69] Widmann A, Schröger E, Maess B (2015). Digital filter design for electrophysiological data—a practical approach. Journal of Neuroscience Methods.

[ref-70] Winkler I, Haufe S, Tangermann M (2011). Automatic classification of artifactual ICA—components for artifact removal in EEG signals. Behavioral and Brain Functions.

[ref-71] Zou Y, Nathan V, Jafari R (2016). Automatic identification of artefact-related indipendent components for artefact removal in EEG recordings. IEEE Journal of Biomedical and Health Informatics.

