# Supplementary figures and images for: A new ICA-based fingerprint method for the automatic removal of physiological artifacts from EEG recordings
Source: PeerJ. 2018 Feb 23;6:e4380. doi: 10.7717/peerj.4380 (PMC5826009; doi:10.7717/peerj.4380)

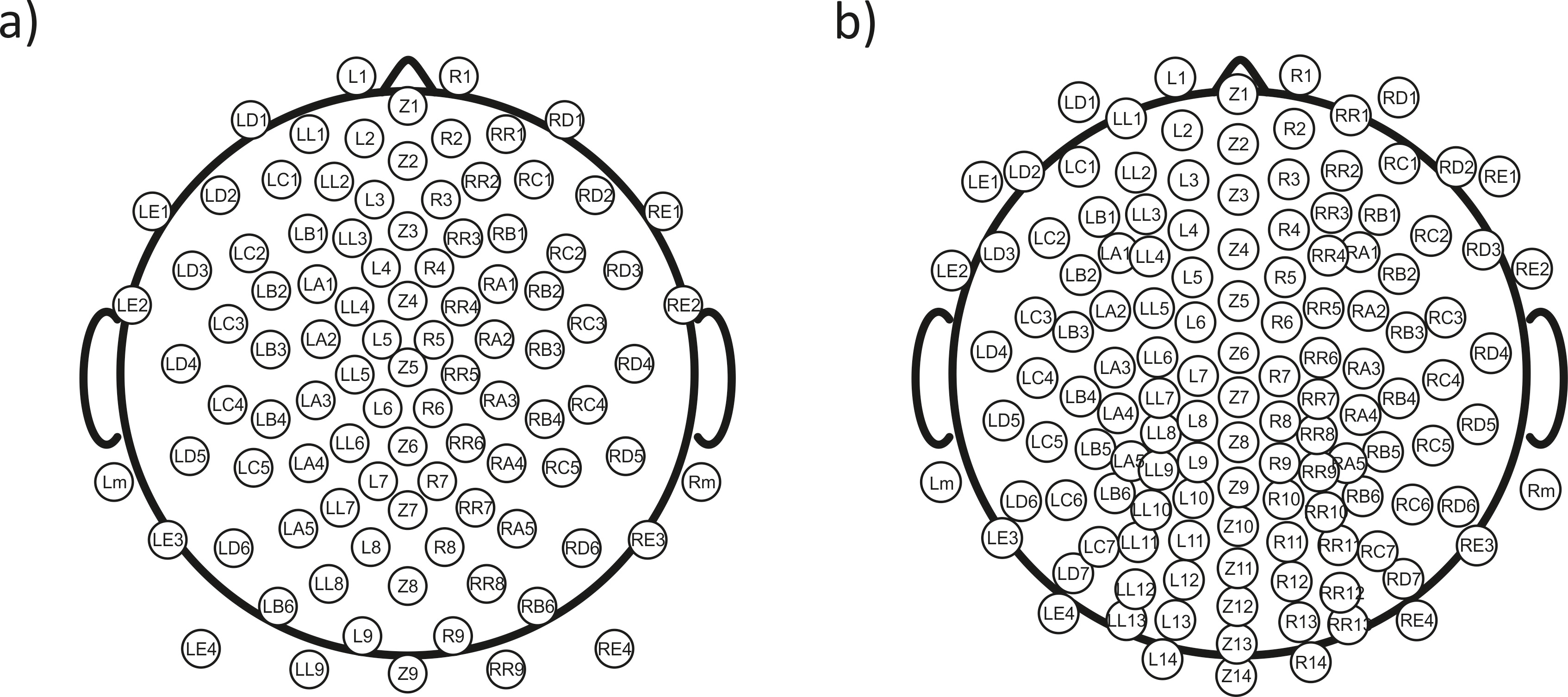

Supplement: Figure S1 — Layout of the caps used for EEG acquisitions: (A) the novel dry electrode cap with 97 dry Multipin Polyurethane electrodes with an Ag/AgCl coating, arranged in an equidistant layout; (B) the commercial wet cap (Waveguard, Advanced Neuro Technologies B.V., Enschede, Netherlands) with 128 Ag/AgCl electrodes in a quasi-equidistant layout. [file peerj-06-4380-s001.png]
