# Supplementary material for: A new ICA-based fingerprint method for the automatic removal of physiological artifacts from EEG recordings
Source: PeerJ. 2018 Feb 23;6:e4380. doi: 10.7717/peerj.4380 (PMC5826009; doi:10.7717/peerj.4380)
Supplement: Table S1 — Composition of the groups of EEG datasets used to train and test each SVM classifier for each artifact. Training and testing groups include wet and dry EEG datasets. Each dataset is identified only by the number. The type of artifact and electrode can be inferred from the position in the table. Datasets used for classifying ICs containing cardiac interference include the acronym of the cued artifact (i.e., EB for eyeblinks and EM for eye movements). [file peerj-06-4380-s002.docx]

| **Groups of training and testing datasets used for the 10 SVMs** | | | | | |
| --- | --- | --- | --- | --- | --- |
|  |  | **Training datasets** | | **Testing datasets** | |
|  |  | WET | DRY | WET | DRY |
| Eye Blink | SVM1 | 3 - 5 - 6 - 9 - 11 - 12 | 1 - 2 - 5 - 6 - 7 - 11 - 12 | 1 - 2 - 4 - 7 - 8 - 10 | 3 - 4 - 7 - 8 - 9 - 10 |
|  | SVM2 | 4 - 5 - 6 - 7 - 8 - 11 | 1 - 4 - 6 - 8 - 9 - 12 | 1 - 2 - 3 - 9 - 10 - 12 | 2 - 3 - 5 - 7 - 10 - 11 |
|  | SVM3 | 2 - 3 - 4 - 5 - 6 - 10 | 2 - 5 - 8 - 9 - 10 - 12 | 1 - 7 - 8 - 9 - 11 - 12 | 1 - 3 - 4 - 6 - 7 - 11 |
|  | SVM4 | 1 - 2 - 4 - 6 - 10 - 11 | 1 - 3 - 4 - 7 - 8 - 10 | 3 - 5 - 7 - 8 - 9 - 12 | 2 - 5 - 6 - 9 - 11 - 12 |
|  | SVM5 | 3 - 5 - 7 - 8 - 10 - 11 | 1 - 2 - 3 - 5 - 10 - 11 | 1 - 2 - 4 - 6 - 9 - 12 | 4 - 6 - 7 - 8 - 9 - 12 |
|  | SVM6 | 1 - 2 - 3 - 4 - 5 - 12 | 2 - 3 - 9 - 10 - 11 - 12 | 6 - 7 - 8 - 9 - 10 - 11 | 1 - 4 - 5 - 6 - 7 - 8 |
|  | SVM7 | 3 - 4 - 5 - 6 - 7 - 8 | 1 - 2 - 6 - 9 - 11 - 12 | 1 - 2 - 9 - 10 - 11 - 12 | 3 - 4 - 5 - 7 - 8 - 10 |
|  | SVM8 | 1 - 2 - 4 - 7 - 9 - 11 | 1 - 2 - 3 - 5 - 7 - 9 | 3 - 5 - 6 - 8 - 10 - 12 | 4 - 6 - 8 - 10 - 11 - 12 |
|  | SVM9 | 2 - 3 - 4 - 9 - 10 - 11 | 6 - 7 - 8 - 9 - 10 - 11 | 1 - 5 - 6 - 7 - 8 - 12 | 1 - 2 - 3 - 4 - 5 - 12 |
|  | SVM10 | 1 - 5 - 6 - 7 - 8 - 12 | 1 - 2 - 3 - 4 - 5 - 12 | 2 - 3 - 4 - 9 - 10 - 11 | 6 - 7 - 8 - 9 - 10 - 11 |
|  |  | WET | DRY | WET | DRY |
| Eye Movement | SVM1 | 1 - 3 - 4 - 6 - 7 - 10 | 3 - 4 - 6 - 7 - 9 | 2 - 5 - 8 - 9 | 1 - 2 - 5 - 8 |
|  | SVM2 | 1 - 2 - 5 - 6 - 7 - 9 | 1 - 4 - 5 - 7 - 8 | 3 - 4 - 8 - 10 | 2 - 3 - 6 - 9 |
|  | SVM3 | 3 - 4 - 5 - 8 - 9 - 10 | 1 - 2 - 4 - 6 - 9 | 1 - 2 - 6 - 7 | 3 - 5 - 7 - 8 |
|  | SVM4 | 1 - 2 - 4 - 6 - 8 - 9 | 2 - 3 - 5 - 6 - 8 | 3 - 5 - 7 - 10 | 1 - 4 - 7 - 9 |
|  | SVM5 | 2 - 3 - 6 - 7 - 9 - 10 | 1 - 4 - 5 - 6 - 9 | 1 - 4 - 5 - 8 | 2 - 3 - 7 - 8 |
|  | SVM6 | 3 - 4 - 5 - 7 - 8 - 10 | 2 - 3 - 6 - 7 - 8 | 1 - 2 - 6 - 9 | 1 - 4 - 5 - 9 |
|  | SVM7 | 1 - 2 - 5 - 7 - 9 - 10 | 1 - 3 - 5 - 7 - 8 | 3 - 4 - 6 - 8 | 2 - 4 - 6 - 9 |
|  | SVM8 | 2 - 3 - 4 - 6 - 8 - 9 | 2 - 3 - 4 - 7 - 9 | 1 - 5 - 7 - 10 | 1 - 5 - 6 - 8 |
|  | SVM9 | 1 - 2 - 3 - 4 - 6 - 9 | 1 - 2 - 4 - 7 - 8 | 5 - 7 - 8 - 10 | 3 - 5 - 6 - 9 |
|  | SVM10 | 1 - 2 - 7 - 8 - 9 - 10 | 5 - 6 - 7 - 8 - 9 | 3 - 4 - 5 - 6 | 1 - 2 - 3 - 4 |
|  |  | WET | DRY | WET | DRY |
| Myogenic artifact | SVM1 | 2 - 3 - 5 - 7 - 9 | 2 - 4 - 5 - 8 | 1 - 4 - 6 - 8 - 10 | 1 - 3 - 6 - 7 - 9 |
|  | SVM2 | 1 - 2 - 4 - 6 - 8 | 1 - 3 - 7 - 9 | 3 - 5 - 7 - 9 - 10 | 2 - 4 - 5 - 6 - 8 |
|  | SVM3 | 2 - 5 - 6 - 7 - 8 | 2 - 6 - 7 - 8 | 1 - 3 - 4 - 9 - 10 | 1 - 3 - 4 - 5 - 9 |
|  | SVM4 | 1 - 2 - 3 - 5 - 7 | 2 - 5 - 6 - 9 | 4 - 6 - 8 - 9 - 10 | 1 - 3 - 4 - 7 - 8 |
|  | SVM5 | 4 - 5 - 7 - 8 - 9 | 1 - 2 - 6 - 9 | 1 - 2 - 3 - 6 - 10 | 3 - 4 - 5 - 7 - 8 |
|  | SVM6 | 1 - 3 - 4 - 7 - 10 | 3 - 4 - 6 - 7 | 2 - 5 - 6 - 8 - 9 | 1 - 2 - 5 - 8 - 9 |
|  | SVM7 | 3 - 5 - 6 - 8 - 9 | 1 - 3 - 4 - 8 | 1 - 2 - 4 - 7 - 10 | 2 - 5 - 6 - 7 - 9 |
|  | SVM8 | 2 - 4 - 5 - 7 - 10 | 3 - 4 - 5 - 8 | 1 - 3 - 6 - 8 - 9 | 1 - 2 - 6 - 7 - 9 |
|  | SVM9 | 1 - 2 - 4 - 6 - 9 | 3 - 5 - 7 - 9 | 3 - 5 - 7 - 8 - 10 | 1 - 2 - 4 - 6 - 8 |
|  | SVM10 | 3 - 5 - 7 - 8 - 10 | 1 - 4 - 6 - 8 | 1 - 2 - 4 - 6 - 9 | 2 - 3 - 5 - 7 - 9 |
|  |  | WET | DRY | WET | DRY |
| Cardiac interference | SVM1 | EB-1 EB-3 EB-5 EB-8 EM-2 EM-3 EM-4 EM-10 | EB-4 EB-8 EB-12  EM-2 EM-6 EM-9 | EB-10 EB-12 EM-6 EM-7 EM-8 | EB-1 EB-2 EB-3  EM-5 EM-8 |
|  | SVM2 | EB-1 EB-10 EB-12 EM-2 EM-3 EM-4 EM-6 EM-7 | EB-1 EB-3 EM-2  EM-5 EM-8 EM-9 | EB-3 EB-5 EB-8  EM-8 EM-10 | EB-2 EB-4 EB-8  EB-12 EM-6 |
|  | SVM3 | EB-1 EB-5 EB-8 EM-2 EM-3 EM-6 EM-7 EM-8 | EB-3 EB-4 EB-12  EM-5 EM-8 EM-9 | EB-3 EB-10 EB-12 EM-4 EM-10 | EB-1 EB-2 EB-8  EM-2 EM-6 |
|  | SVM4 | EB-3 EB-5 EB-8 EB-12 EM-4 EM-6 EM-7 EM-8 | EB-1 EB-2 EB-4  EB-12 EM-5 EM-6 | EB-1 EB-10 EM-2  EM-3 EM-10 | EB-3 EB-8 EM-2  EM-8 EM-9 |
|  | SVM5 | EB-8 EB-10 EB-12 EM-2 EM-4 EM-6 EM-8 EM-10 | EB-1 EB-3 EB-12  EM-2 EM-5 EM-9 | EB-1 EB-3 EB-5  EM-3 EM-7 | EB-2 EB-4 EB-8  EM-6 EM-8 |
|  | SVM6 | EB-3 EB-5 EB-8 EM-2 EM-3 EM-4 EM-7 EM-8 | EB-2 EB-3 EB-8  EM-6 EM-8 EM-9 | EB-1 EB-10 EB-12 EM-6 EM-10 | EB-1 EB-4 EB-12  EM-2 EM-5 |
|  | SVM7 | EB-1 EB-3 EB-5 EB-8 EB-10 EB-12 EM-3 EM-4 | EB-1 EB-2 EB-3  EB-4 EB-8 EB-12 | EM-2 EM-6 EM-7 EM-8 EM-10 | EM-2 EM-5 EM-6 EM-8 EM-9 |
|  | SVM8 | EB-1 EB-12 EM-2 EM-4  EM-6 EM-7 EM-8 EM-10 | EB-2 EM-2 EM-5  EM-6 EM-8 EM-9 | EB-3 EB-5 EB-8  EB-10 EM-3 | EB-1 EB-3 EB-4  EB-8 EB-12 |
|  | SVM9 | EB-3 EB-5 EB-10 EB-12  EM-2 EM-3 EM-7 EM-10 | EB-2 EB-4 EB-8  EM-2 EM-5 EM-8 | EB-1 EB-8 EM-4  EM-6 EM-8 | EB-1 EB-3 EB-12  EM-6 EM-9 |
|  | SVM10 | EB-3 EB-5 EB-8 EB-10  EB-12 EM-2 EM-8 EM-10 | EB-1 EB-2 EB-8  EM-5 EM-6 EM-8 | EB-1 EM-3 EM-4 EM-6 EM-7 | EB-3 EB-4 EB-12  EM-2 EM-9 |
