# Supplementary material for: A new ICA-based fingerprint method for the automatic removal of physiological artifacts from EEG recordings
Source: PeerJ. 2018 Feb 23;6:e4380. doi: 10.7717/peerj.4380 (PMC5826009; doi:10.7717/peerj.4380)
Supplement: Table S2 — The statistical performance of the individual SVM classifiers trained to classify ICs containing eyeblinks is reported separately for the wet and dry testing EEG datasets and for the three decomposition levels (20, 50 and 80 ICs). [file peerj-06-4380-s003.docx]

| **Eyeblink SVM classifiers performance** | | | | | | | | | | | | | | |
| --- | --- | --- | --- | --- | --- | --- | --- | --- | --- | --- | --- | --- | --- | --- |
| **Eye Blink** | **N. of ICs per dataset** | **Electrode type** | **N. of datasets** | **Total N. of ICs** | **Total N. of artifactual ICs** | **True positive** | **True negative** | **False positive** | **False negative** | **Accuracy** | **FOR** | **HR** | **FAR (*g*)** | **Sensitivity *p*** |
| SVM-1 | 20 | WET | 6 | 120 | 7 | 5 | 113 | 0 | 2 | 0.983 | 0.017 | 0.714 | 0 | 0.714 |
|  |  | DRY | 6 | 120 | 7 | 6 | 113 | 0 | 1 | 0.992 | 0.009 | 0.857 | 0 | 0.857 |
|  | 50 | WET | 6 | 300 | 7 | 5 | 293 | 0 | 2 | 0.993 | 0.007 | 0.714 | 0 | 0.714 |
|  |  | DRY | 6 | 300 | 7 | 5 | 293 | 0 | 2 | 0.993 | 0.007 | 0.714 | 0 | 0.714 |
|  | 80 | WET | 6 | 480 | 7 | 5 | 473 | 0 | 2 | 0.996 | 0.004 | 0.714 | 0 | 0.714 |
|  |  | DRY | 6 | 480 | 7 | 5 | 473 | 0 | 2 | 0.996 | 0.004 | 0.714 | 0 | 0.714 |
| SVM-2 | 20 | WET | 6 | 120 | 7 | 5 | 113 | 0 | 2 | 0.983 | 0.017 | 0.714 | 0 | 0.714 |
|  |  | DRY | 6 | 120 | 7 | 6 | 113 | 0 | 1 | 0.992 | 0.009 | 0.857 | 0 | 0.857 |
|  | 50 | WET | 6 | 300 | 7 | 6 | 293 | 0 | 1 | 0.997 | 0.003 | 0.857 | 0 | 0.857 |
|  |  | DRY | 6 | 300 | 7 | 5 | 293 | 0 | 2 | 0.993 | 0.007 | 0.714 | 0 | 0.714 |
|  | 80 | WET | 6 | 480 | 7 | 6 | 473 | 0 | 1 | 0.998 | 0.002 | 0.857 | 0 | 0.857 |
|  |  | DRY | 6 | 480 | 7 | 5 | 473 | 0 | 2 | 0.996 | 0.004 | 0.714 | 0 | 0.714 |
| SVM-3 | 20 | WET | 6 | 120 | 6 | 6 | 114 | 0 | 0 | 1 | 0 | 1 | 0 | 1 |
|  |  | DRY | 6 | 120 | 7 | 7 | 113 | 0 | 0 | 1 | 0 | 1 | 0 | 1 |
|  | 50 | WET | 6 | 300 | 6 | 6 | 294 | 0 | 0 | 1 | 0 | 1 | 0 | 1 |
|  |  | DRY | 6 | 300 | 7 | 7 | 293 | 0 | 0 | 1 | 0 | 1 | 0 | 1 |
|  | 80 | WET | 6 | 480 | 6 | 6 | 474 | 0 | 0 | 1 | 0 | 1 | 0 | 1 |
|  |  | DRY | 6 | 480 | 7 | 7 | 473 | 0 | 0 | 1 | 0 | 1 | 0 | 1 |
| SVM-4 | 20 | WET | 6 | 120 | 6 | 6 | 114 | 0 | 0 | 1 | 0 | 1 | 0 | 1 |
|  |  | DRY | 6 | 120 | 7 | 7 | 113 | 0 | 0 | 1 | 0 | 1 | 0 | 1 |
|  | 50 | WET | 6 | 300 | 6 | 6 | 294 | 0 | 0 | 1 | 0 | 1 | 0 | 1 |
|  |  | DRY | 6 | 300 | 7 | 7 | 293 | 0 | 0 | 1 | 0 | 1 | 0 | 1 |
|  | 80 | WET | 6 | 480 | 6 | 6 | 474 | 0 | 0 | 1 | 0 | 1 | 0 | 1 |
|  |  | DRY | 6 | 480 | 7 | 7 | 473 | 0 | 0 | 1 | 0 | 1 | 0 | 1 |
| SVM-5 | 20 | WET | 6 | 120 | 6 | 6 | 114 | 0 | 0 | 1 | 0 | 1 | 0 | 1 |
|  |  | DRY | 6 | 120 | 8 | 8 | 112 | 0 | 0 | 1 | 0 | 1 | 0 | 1 |
|  | 50 | WET | 6 | 300 | 6 | 6 | 294 | 0 | 0 | 1 | 0 | 1 | 0 | 1 |
|  |  | DRY | 6 | 300 | 8 | 8 | 292 | 0 | 0 | 1 | 0 | 1 | 0 | 1 |
|  | 80 | WET | 6 | 480 | 6 | 6 | 474 | 0 | 0 | 1 | 0 | 1 | 0 | 1 |
|  |  | DRY | 6 | 480 | 8 | 8 | 472 | 0 | 0 | 1 | 0 | 1 | 0 | 1 |
| SVM-6 | 20 | WET | 6 | 120 | 7 | 7 | 113 | 0 | 0 | 1 | 0 | 1 | 0 | 1 |
|  |  | DRY | 6 | 120 | 7 | 7 | 113 | 0 | 0 | 1 | 0 | 1 | 0 | 1 |
|  | 50 | WET | 6 | 300 | 7 | 7 | 293 | 0 | 0 | 1 | 0 | 1 | 0 | 1 |
|  |  | DRY | 6 | 300 | 7 | 7 | 293 | 0 | 0 | 1 | 0 | 1 | 0 | 1 |
|  | 80 | WET | 6 | 480 | 7 | 7 | 473 | 0 | 0 | 1 | 0 | 1 | 0 | 1 |
|  |  | DRY | 6 | 480 | 7 | 7 | 473 | 0 | 0 | 1 | 0 | 1 | 0 | 1 |
| SVM-7 | 20 | WET | 6 | 120 | 7 | 5 | 113 | 0 | 2 | 0.983 | 0.017 | 0.714 | 0 | 0.714 |
|  |  | DRY | 6 | 120 | 7 | 6 | 113 | 0 | 1 | 0.992 | 0.009 | 0.857 | 0 | 0.857 |
|  | 50 | WET | 6 | 300 | 7 | 6 | 293 | 0 | 1 | 0.997 | 0.003 | 0.857 | 0 | 0.857 |
|  |  | DRY | 6 | 300 | 7 | 5 | 293 | 0 | 2 | 0.993 | 0.007 | 0.714 | 0 | 0.714 |
|  | 80 | WET | 6 | 480 | 7 | 6 | 473 | 0 | 1 | 0.998 | 0.002 | 0.857 | 0 | 0.857 |
|  |  | DRY | 6 | 480 | 7 | 5 | 473 | 0 | 2 | 0.996 | 0.004 | 0.714 | 0 | 0.714 |
| SVM-8 | 20 | WET | 6 | 120 | 7 | 6 | 113 | 0 | 1 | 0.992 | 0.009 | 0.857 | 0 | 0.857 |
|  |  | DRY | 6 | 120 | 7 | 6 | 113 | 0 | 1 | 0.992 | 0.009 | 0.857 | 0 | 0.857 |
|  | 50 | WET | 6 | 300 | 7 | 6 | 293 | 0 | 1 | 0.997 | 0.003 | 0.857 | 0 | 0.857 |
|  |  | DRY | 6 | 300 | 7 | 6 | 293 | 0 | 1 | 0.997 | 0.003 | 0.857 | 0 | 0.857 |
|  | 80 | WET | 6 | 480 | 7 | 6 | 473 | 0 | 1 | 0.998 | 0.002 | 0.857 | 0 | 0.857 |
|  |  | DRY | 6 | 480 | 7 | 6 | 473 | 0 | 1 | 0.998 | 0.002 | 0.857 | 0 | 0.857 |
| SVM-9 | 20 | WET | 6 | 120 | 6 | 6 | 114 | 0 | 0 | 1 | 0 | 1 | 0 | 1 |
|  |  | DRY | 6 | 120 | 7 | 7 | 113 | 0 | 0 | 1 | 0 | 1 | 0 | 1 |
|  | 50 | WET | 6 | 300 | 6 | 6 | 294 | 0 | 0 | 1 | 0 | 1 | 0 | 1 |
|  |  | DRY | 6 | 300 | 7 | 7 | 293 | 0 | 0 | 1 | 0 | 1 | 0 | 1 |
|  | 80 | WET | 6 | 480 | 6 | 6 | 474 | 0 | 0 | 1 | 0 | 1 | 0 | 1 |
|  |  | DRY | 6 | 480 | 7 | 7 | 473 | 0 | 0 | 1 | 0 | 1 | 0 | 1 |
| SVM-10 | 20 | WET | 6 | 120 | 7 | 5 | 113 | 0 | 2 | 0.983 | 0.017 | 0.714 | 0 | 0.714 |
|  |  | DRY | 6 | 120 | 7 | 6 | 113 | 0 | 1 | 0.992 | 0.009 | 0.857 | 0 | 0.857 |
|  | 50 | WET | 6 | 300 | 7 | 6 | 293 | 0 | 1 | 0.997 | 0.003 | 0.857 | 0 | 0.857 |
|  |  | DRY | 6 | 300 | 7 | 5 | 293 | 0 | 2 | 0.993 | 0.007 | 0.714 | 0 | 0.714 |
|  | 80 | WET | 6 | 480 | 7 | 6 | 473 | 0 | 1 | 0.998 | 0.002 | 0.857 | 0 | 0.857 |
|  |  | DRY | 6 | 480 | 7 | 5 | 473 | 0 | 2 | 0.996 | 0.004 | 0.714 | 0 | 0.714 |
| ***AVERAGE VALUES on all SVMs***  ***(Mean±SD)*** | *20* | *WET* | *6* | *120* | *6.6±*  *0.5* | *5.7±*  *0.7* | *113.4±*  *0.5* | *0* | *0.9±*  *1.0* | *0.993±*  *0.004* | *0.008±*  *0.009* | *0.871±*  *0.142* | *0* | *0.871±*  *0.142* |
|  |  | *DRY* | *6* | *120* | *7.1±*  *0.3* | *6.6±*  *0.7* | *112.9±*  *0.3* | *0* | *0.5±*  *0.5* | *0.996±*  *0.002* | *0.004±*  *0.005* | *0.929±*  *0.075* | *0* | *0.929±*  *0.075* |
|  | *50* | *WET* | *6* | *300* | *6.6±*  *0.5* | *6.0±*  *0.5* | *293.4±*  *0.5* | *0* | *0.6±*  *0.7* | *0.998±*  *0.003* | *0.002±*  *0.002* | *0.914±*  *0.100* | *0* | *0.914±*  *0.100* |
|  |  | *DRY* | *6* | *300* | *7.1±*  *0.3* | *6.2±*  *1.1* | *292.9±*  *0.3* | *0* | *0.9±*  *1.0* | *0.997±*  *0.001* | *0.003±*  *0.003* | *0.871±*  *0.142* | *0* | *0.871±*  *0.142* |
|  | *80* | *WET* | *6* | *480* | *6.6±*  *0.5* | *6.0±*  *0.5* | *473.4±*  *0.5* | *0* | *0.6±*  *0.7* | *0.999±*  *0.002* | *0.001±*  *0.001* | *0.914±*  *0.100* | *0* | *0.914±*  *0.100* |
|  |  | *DRY* | *6* | *480* | *7.1±*  *0.3* | *6.2±*  *1.1* | *472.9±*  *0.3* | *0* | *0.9±*  *1.0* | *0.998±*  *0.007* | *0.002±*  *0.002* | *0.871±*  *0.142* | *0* | *0.871±*  *0.142* |
