# Supplementary material for: A new ICA-based fingerprint method for the automatic removal of physiological artifacts from EEG recordings
Source: PeerJ. 2018 Feb 23;6:e4380. doi: 10.7717/peerj.4380 (PMC5826009; doi:10.7717/peerj.4380)
Supplement: Table S3 — The statistical performance of the individual SVM classifiers trained to classify ICs containing eye movements is reported separately for the wet and dry testing EEG datasets and for the three decomposition levels (20, 50 and 80 ICs). [file peerj-06-4380-s004.docx]

| **Eye movement SVM classifiers performance** | | | | | | | | | | | | | | |  |
| --- | --- | --- | --- | --- | --- | --- | --- | --- | --- | --- | --- | --- | --- | --- | --- |
| **Eye movement** | **N. of ICs per dataset** | **Electrode type** | **N. of datasets** | **Total N. of ICs** | **Total N. of artifactual ICs** | **True positive** | **True negative** | **False positive** | **False negative** | **Accuracy** | **FOR** | **HR** | **FAR (*g*)** | **Sensitivity *p*** | |
| SVM-1 | 20 | WET | 4 | 80 | 27 | 23 | 52 | 1 | 4 | 0.938 | 0.071 | 0.852 | 0.019 | 0.849 | |
|  |  | DRY | 4 | 80 | 10 | 9 | 68 | 2 | 1 | 0.963 | 0.014 | 0.900 | 0.029 | 0.897 | |
|  | 50 | WET | 4 | 200 | 66 | 64 | 128 | 6 | 2 | 0.960 | 0.015 | 0.970 | 0.045 | 0.968 | |
|  |  | DRY | 4 | 200 | 37 | 37 | 158 | 5 | 0 | 0.975 | 0 | 1 | 0.031 | 1 | |
|  | 80 | WET | 4 | 320 | 130 | 118 | 182 | 8 | 12 | 0.938 | 0.062 | 0.908 | 0.042 | 0.904 | |
|  |  | DRY | 4 | 320 | 63 | 59 | 237 | 20 | 4 | 0.925 | 0.017 | 0.937 | 0.078 | 0.931 | |
| SVM-2 | 20 | WET | 4 | 80 | 19 | 18 | 60 | 1 | 1 | 0.975 | 0.016 | 0.947 | 0.016 | 0.946 | |
|  |  | DRY | 4 | 80 | 14 | 14 | 65 | 1 | 0 | 0.988 | 0 | 1 | 0.015 | 1 | |
|  | 50 | WET | 4 | 200 | 53 | 53 | 140 | 7 | 0 | 0.965 | 0 | 1 | 0.048 | 1 | |
|  |  | DRY | 4 | 200 | 43 | 43 | 150 | 7 | 0 | 0.965 | 0 | 1 | 0.045 | 1 | |
|  | 80 | WET | 4 | 320 | 96 | 93 | 209 | 15 | 3 | 0.944 | 0.014 | 0.969 | 0.067 | 0.967 | |
|  |  | DRY | 4 | 320 | 73 | 71 | 239 | 8 | 2 | 0.969 | 0.008 | 0.973 | 0.032 | 0.972 | |
| SVM-3 | 20 | WET | 4 | 80 | 27 | 25 | 52 | 1 | 2 | 0.963 | 0.037 | 0.926 | 0.019 | 0.925 | |
|  |  | DRY | 4 | 80 | 19 | 17 | 59 | 2 | 2 | 0.950 | 0.033 | 0.895 | 0.033 | 0.891 | |
|  | 50 | WET | 4 | 200 | 66 | 66 | 127 | 6 | 1 | 0.965 | 0.008 | 0.985 | 0.045 | 0.984 | |
|  |  | DRY | 4 | 200 | 48 | 46 | 142 | 5 | 7 | 0.940 | 0.047 | 0.868 | 0.034 | 0.863 | |
|  | 80 | WET | 4 | 320 | 134 | 123 | 175 | 11 | 11 | 0.931 | 0.059 | 0.918 | 0.059 | 0.913 | |
|  |  | DRY | 4 | 320 | 100 | 87 | 216 | 4 | 13 | 0.947 | 0.057 | 0.870 | 0.018 | 0.868 | |
| SVM-4 | 20 | WET | 4 | 80 | 23 | 23 | 55 | 2 | 0 | 0.975 | 0 | 1 | 0.035 | 1 | |
|  |  | DRY | 4 | 80 | 15 | 13 | 61 | 4 | 2 | 0.925 | 0.032 | 0.867 | 0.062 | 0.858 | |
|  | 50 | WET | 4 | 200 | 62 | 62 | 131 | 7 | 0 | 0.965 | 0 | 1 | 0.051 | 1 | |
|  |  | DRY | 4 | 200 | 41 | 38 | 153 | 6 | 3 | 0.955 | 0.019 | 0.927 | 0.038 | 0.924 | |
|  | 80 | WET | 4 | 320 | 121 | 116 | 190 | 9 | 5 | 0.956 | 0.026 | 0.959 | 0.045 | 0.957 | |
|  |  | DRY | 4 | 320 | 57 | 52 | 249 | 15 | 5 | 0.938 | 0.020 | 0.912 | 0.057 | 0.907 | |
| SVM-5 | 20 | WET | 4 | 80 | 25 | 23 | 55 | 0 | 2 | 0.975 | 0.035 | 0.920 | 0 | 0.920 | |
|  |  | DRY | 4 | 80 | 14 | 12 | 64 | 2 | 2 | 0.950 | 0.030 | 0.857 | 0.030 | 0.853 | |
|  | 50 | WET | 4 | 200 | 61 | 60 | 132 | 7 | 1 | 0.960 | 0.008 | 0.984 | 0.050 | 0.983 | |
|  |  | DRY | 4 | 200 | 39 | 37 | 151 | 10 | 2 | 0.940 | 0.013 | 0.949 | 0.062 | 0.945 | |
|  | 80 | WET | 4 | 320 | 112 | 106 | 198 | 10 | 6 | 0.950 | 0.029 | 0.946 | 0.048 | 0.944 | |
|  |  | DRY | 4 | 320 | 74 | 65 | 236 | 10 | 9 | 0.941 | 0.037 | 0.878 | 0.041 | 0.873 | |
| SVM-6 | 20 | WET | 4 | 80 | 28 | 23 | 50 | 2 | 5 | 0.913 | 0.091 | 0.821 | 0.038 | 0.814 | |
|  |  | DRY | 4 | 80 | 11 | 10 | 66 | 3 | 1 | 0.950 | 0.015 | 0.909 | 0.043 | 0.905 | |
|  | 50 | WET | 4 | 200 | 63 | 62 | 128 | 9 | 1 | 0.950 | 0.008 | 0.984 | 0.066 | 0.983 | |
|  |  | DRY | 4 | 200 | 39 | 39 | 158 | 3 | 0 | 0.985 | 0 | 1 | 0.019 | 1 | |
|  | 80 | WET | 4 | 320 | 128 | 113 | 181 | 11 | 15 | 0.919 | 0.077 | 0.883 | 0.057 | 0.876 | |
|  |  | DRY | 4 | 320 | 64 | 60 | 236 | 20 | 4 | 0.925 | 0.017 | 0.938 | 0.078 | 0.932 | |
| SVM-7 | 20 | WET | 4 | 80 | 21 | 20 | 58 | 1 | 1 | 0.975 | 0.017 | 0.952 | 0.017 | 0.952 | |
|  |  | DRY | 4 | 80 | 12 | 12 | 66 | 2 | 0 | 0.975 | 0 | 1 | 0.029 | 1 | |
|  | 50 | WET | 4 | 200 | 51 | 50 | 137 | 12 | 1 | 0.935 | 0.007 | 0.980 | 0.081 | 0.979 | |
|  |  | DRY | 4 | 200 | 41 | 41 | 151 | 8 | 0 | 0.960 | 0 | 1 | 0.050 | 1 | |
|  | 80 | WET | 4 | 320 | 99 | 96 | 205 | 16 | 3 | 0.941 | 0.014 | 0.970 | 0.072 | 0.967 | |
|  |  | DRY | 4 | 320 | 62 | 60 | 245 | 13 | 2 | 0.953 | 0.008 | 0.968 | 0.050 | 0.966 | |
| SVM-8 | 20 | WET | 4 | 80 | 27 | 27 | 52 | 1 | 0 | 0.988 | 0 | 1 | 0.019 | 1 | |
|  |  | DRY | 4 | 80 | 14 | 14 | 64 | 2 | 0 | 0.975 | 0 | 1 | 0.030 | 1 | |
|  | 50 | WET | 4 | 200 | 68 | 66 | 128 | 4 | 2 | 0.970 | 0.015 | 0.971 | 0.030 | 0.970 | |
|  |  | DRY | 4 | 200 | 55 | 55 | 143 | 2 | 0 | 0.990 | 0 | 1 | 0.014 | 1 | |
|  | 80 | WET | 4 | 320 | 128 | 120 | 185 | 7 | 8 | 0.953 | 0.041 | 0.938 | 0.036 | 0.935 | |
|  |  | DRY | 4 | 320 | 92 | 86 | 216 | 12 | 6 | 0.944 | 0.027 | 0.935 | 0.053 | 0.931 | |
| SVM-9 | 20 | WET | 4 | 80 | 26 | 24 | 53 | 1 | 2 | 0.963 | 0.036 | 0.923 | 0.019 | 0.922 | |
|  |  | DRY | 4 | 80 | 19 | 18 | 61 | 0 | 1 | 0.988 | 0.016 | 0.947 | 0 | 0.947 | |
|  | 50 | WET | 4 | 200 | 66 | 65 | 129 | 5 | 1 | 0.970 | 0.008 | 0.985 | 0.037 | 0.984 | |
|  |  | DRY | 4 | 200 | 58 | 57 | 137 | 5 | 1 | 0.970 | 0.007 | 0.983 | 0.035 | 0.982 | |
|  | 80 | WET | 4 | 320 | 126 | 122 | 190 | 4 | 4 | 0.975 | 0.021 | 0.968 | 0.021 | 0.968 | |
|  |  | DRY | 4 | 320 | 102 | 93 | 212 | 6 | 9 | 0.953 | 0.041 | 0.912 | 0.028 | 0.909 | |
| SVM-10 | 20 | WET | 4 | 80 | 21 | 20 | 57 | 2 | 1 | 0.963 | 0.017 | 0.952 | 0.034 | 0.951 | |
|  |  | DRY | 4 | 80 | 9 | 8 | 68 | 3 | 1 | 0.950 | 0.014 | 0.889 | 0.042 | 0.884 | |
|  | 50 | WET | 4 | 200 | 52 | 52 | 134 | 14 | 0 | 0.930 | 0 | 1 | 0.095 | 1 | |
|  |  | DRY | 4 | 200 | 21 | 21 | 166 | 13 | 0 | 0.935 | 0 | 1 | 0.073 | 1 | |
|  | 80 | WET | 4 | 320 | 99 | 96 | 203 | 18 | 3 | 0.934 | 0.015 | 0.970 | 0.081 | 0.967 | |
|  |  | DRY | 4 | 320 | 28 | 28 | 268 | 24 | 0 | 0.925 | 0 | 1 | 0.082 | 1 | |
| ***AVERAGE VALUES on all SVMs***  ***(Mean±SD)*** | *20* | *WET* | 4 | 80 | *24.4±*  *3.2* | *22.6±*  *2.6* | *54.4±*  *3.2* | *1.2±*  *0.6* | *1.8±*  *1.6* | *0.963±*  *0.022* | *0.032±*  *0.029* | *0.929±*  *0.057* | *0.022±*  *0.011* | *0.928±*  *0.059* | |
|  |  | *DRY* | 4 | 80 | *13.7±*  *3.4* | *12.7±*  *3.2* | *64.2±*  *3.1* | *2.1±*  *1.1* | *1.0±*  *0.8* | *0.961±*  *0.020* | *0.015±*  *0.013* | *0.926±*  *0.056* | *0.031±*  *0.016* | *0.924±*  *0.059* | |
|  | *50* | *WET* | 4 | 200 | *60.8±*  *6.4* | *60.0±*  *6.1* | *131.4±*  *4.4* | *7.7±*  *3.1* | *0.9±*  *0.7* | *0.957±*  *0.014* | *0.007±*  *0.006* | *0.986±*  *0.011* | *0.055±*  *0.020* | *0.985±*  *0.012* | |
|  |  | *DRY* | 4 | 200 | *42.2±*  *10.3* | *41.4±*  *10.1* | *150.9±*  *8.6* | *6.4±*  *3.3* | *1.3±*  *2.3* | *0.962±*  *0.019* | *0.009±*  *0.015* | *0.973±*  *0.045* | *0.040±*  *0.018* | *0.971±*  *0.047* | |
|  | *80* | *WET* | 4 | 320 | *117.3±*  *14.6* | *110.3±*  *11.6* | *191.8±*  *11.5* | *10.9±*  *4.3* | *7.0±*  *4.3* | *0.944±*  *0.016* | *0.036±*  *0.023* | *0.943±*  *0.031* | *0.053±*  *0.018* | *0.940±*  *0.032* | |
|  |  | *DRY* | 4 | 320 | *71,5±*  *22.3* | *66.1±*  *19.3* | *235.4±*  *17.2* | *13.2±*  *6.6* | *5.4±*  *6.6* | *0.942±*  *0.014* | *0.023±*  *0.017* | *0.932±*  *0.041* | *0.052±*  *0.022* | *0.929±*  *0.042* | |
