# Supplementary material for: A new ICA-based fingerprint method for the automatic removal of physiological artifacts from EEG recordings
Source: PeerJ. 2018 Feb 23;6:e4380. doi: 10.7717/peerj.4380 (PMC5826009; doi:10.7717/peerj.4380)
Supplement: Table S4 — The statistical performance of the individual SVM classifiers trained to classify ICs containing myogenic artifacts is reported separately for the wet and dry testing EEG datasets and for the three decomposition levels (20, 50 and 80 ICs). [file peerj-06-4380-s005.docx]

| **Myogenic artifact SVM classifiers performance** | | | | | | | | | | | | | | |
| --- | --- | --- | --- | --- | --- | --- | --- | --- | --- | --- | --- | --- | --- | --- |
| **myogenic artifacts** | **N. of ICs per dataset** | **Electrode type** | **N. of datasets** | **Total N. of ICs** | **Total N. of artifactual ICs** | **True positive** | **True negative** | **False positive** | **False negative** | **Accuracy** | **FOR** | **HR** | **FAR (*g*)** | **Sensitivity *p*** |
| SVM-1 | 20 | WET | 5 | 100 | 61 | 61 | 30 | 9 | 0 | 0.910 | 0 | 1 | 0.231 | 1 |
|  |  | DRY | 5 | 100 | 24 | 24 | 72 | 4 | 0 | 0.960 | 0 | 1 | 0.053 | 1 |
|  | 50 | WET | 5 | 250 | 137 | 137 | 106 | 7 | 0 | 0.972 | 0 | 1 | 0.062 | 1 |
|  |  | DRY | 5 | 250 | 39 | 39 | 205 | 6 | 0 | 0.976 | 0 | 1 | 0.028 | 1 |
|  | 80 | WET | 5 | 400 | 154 | 151 | 237 | 9 | 3 | 0.970 | 0.013 | 0.981 | 0.037 | 0.980 |
|  |  | DRY | 5 | 400 | 46 | 45 | 352 | 2 | 1 | 0.993 | 0.003 | 0.978 | 0.006 | 0.978 |
| SVM-2 | 20 | WET | 5 | 100 | 73 | 70 | 25 | 2 | 3 | 0.950 | 0.107 | 0.959 | 0.074 | 0.956 |
|  |  | DRY | 5 | 100 | 45 | 43 | 49 | 6 | 2 | 0.920 | 0.039 | 0.956 | 0.109 | 0.950 |
|  | 50 | WET | 5 | 250 | 151 | 150 | 95 | 4 | 1 | 0.980 | 0.010 | 0.993 | 0.040 | 0.993 |
|  |  | DRY | 5 | 250 | 76 | 72 | 171 | 3 | 4 | 0.972 | 0.023 | 0.947 | 0.017 | 0.946 |
|  | 80 | WET | 5 | 400 | 158 | 158 | 237 | 5 | 0 | 0.988 | 0 | 1 | 0.021 | 1 |
|  |  | DRY | 5 | 400 | 85 | 77 | 314 | 1 | 8 | 0.978 | 0.025 | 0.906 | 0.003 | 0.906 |
| SVM-3 | 20 | WET | 5 | 100 | 80 | 79 | 14 | 6 | 1 | 0.930 | 0.067 | 0.988 | 0.300 | 0.982 |
|  |  | DRY | 5 | 100 | 28 | 28 | 67 | 5 | 0 | 0.950 | 0 | 1 | 0.069 | 1 |
|  | 50 | WET | 5 | 250 | 173 | 172 | 72 | 5 | 1 | 0.976 | 0.014 | 0.994 | 0.065 | 0.994 |
|  |  | DRY | 5 | 250 | 51 | 51 | 196 | 3 | 0 | 0.988 | 0 | 1 | 0.015 | 1 |
|  | 80 | WET | 5 | 400 | 186 | 186 | 204 | 10 | 0 | 0.975 | 0 | 1 | 0.047 | 1 |
|  |  | DRY | 5 | 400 | 62 | 61 | 338 | 0 | 1 | 0.998 | 0.003 | 0.984 | 0 | 0.984 |
| SVM-4 | 20 | WET | 5 | 100 | 64 | 64 | 28 | 8 | 0 | 0.920 | 0 | 1 | 0.222 | 1 |
|  |  | DRY | 5 | 100 | 17 | 16 | 78 | 5 | 1 | 0.940 | 0.013 | 0.941 | 0.060 | 0.937 |
|  | 50 | WET | 5 | 250 | 138 | 138 | 108 | 4 | 0 | 0.984 | 0 | 1 | 0.036 | 1 |
|  |  | DRY | 5 | 250 | 35 | 35 | 212 | 3 | 0 | 0.988 | 0 | 1 | 0.014 | 1 |
|  | 80 | WET | 5 | 400 | 153 | 152 | 241 | 6 | 1 | 0.983 | 0.004 | 0.993 | 0.024 | 0.993 |
|  |  | DRY | 5 | 400 | 44 | 41 | 356 | 0 | 3 | 0.993 | 0.008 | 0.932 | 0 | 0.932 |
| SVM-5 | 20 | WET | 5 | 100 | 76 | 74 | 19 | 5 | 2 | 0.930 | 0.095 | 0.974 | 0.208 | 0.967 |
|  |  | DRY | 5 | 100 | 32 | 32 | 63 | 5 | 0 | 0.950 | 0 | 1 | 0.074 | 1 |
|  | 50 | WET | 5 | 250 | 158 | 157 | 86 | 6 | 1 | 0.972 | 0.011 | 0.994 | 0.065 | 0.993 |
|  |  | DRY | 5 | 250 | 55 | 55 | 191 | 4 | 0 | 0.984 | 0 | 1 | 0.021 | 1 |
|  | 80 | WET | 5 | 400 | 167 | 167 | 221 | 12 | 0 | 0.970 | 0 | 1 | 0.052 | 1 |
|  |  | DRY | 5 | 400 | 65 | 63 | 335 | 0 | 2 | 0.995 | 0.006 | 0.969 | 0 | 0.969 |
| SVM-6 | 20 | WET | 5 | 100 | 65 | 63 | 32 | 3 | 2 | 0.950 | 0.059 | 0.969 | 0.086 | 0.966 |
|  |  | DRY | 5 | 100 | 30 | 29 | 70 | 0 | 1 | 0.990 | 0.014 | 0.967 | 0 | 0.967 |
|  | 50 | WET | 5 | 250 | 133 | 129 | 111 | 6 | 4 | 0.960 | 0.035 | 0.970 | 0.051 | 0.968 |
|  |  | DRY | 5 | 250 | 45 | 40 | 203 | 2 | 5 | 0.972 | 0.024 | 0.889 | 0.010 | 0.888 |
|  | 80 | WET | 5 | 400 | 142 | 140 | 251 | 7 | 2 | 0.978 | 0.008 | 0.986 | 0.027 | 0.986 |
|  |  | DRY | 5 | 400 | 54 | 49 | 345 | 1 | 5 | 0.985 | 0.014 | 0.907 | 0.003 | 0.907 |
| SVM-7 | 20 | WET | 5 | 100 | 68 | 66 | 26 | 6 | 2 | 0.920 | 0.071 | 0.971 | 0.188 | 0.964 |
|  |  | DRY | 5 | 100 | 42 | 39 | 55 | 3 | 3 | 0.940 | 0.052 | 0.929 | 0.052 | 0.925 |
|  | 50 | WET | 5 | 250 | 152 | 148 | 95 | 3 | 4 | 0.972 | 0.040 | 0.974 | 0.031 | 0.973 |
|  |  | DRY | 5 | 250 | 65 | 56 | 184 | 1 | 9 | 0.960 | 0.047 | 0.862 | 0.005 | 0.861 |
|  | 80 | WET | 5 | 400 | 162 | 160 | 233 | 5 | 2 | 0.983 | 0.009 | 0.988 | 0.021 | 0.987 |
|  |  | DRY | 5 | 400 | 71 | 64 | 328 | 1 | 7 | 0.980 | 0.021 | 0.901 | 0.003 | 0.901 |
| SVM-8 | 20 | WET | 5 | 100 | 71 | 71 | 25 | 4 | 0 | 0.960 | 0 | 1 | 0.138 | 1 |
|  |  | DRY | 5 | 100 | 27 | 27 | 70 | 3 | 0 | 0.970 | 0 | 1 | 0.041 | 1 |
|  | 50 | WET | 5 | 250 | 151 | 151 | 92 | 7 | 0 | 0.972 | 0 | 1 | 0.071 | 1 |
|  |  | DRY | 5 | 250 | 45 | 45 | 200 | 5 | 0 | 0.980 | 0 | 1 | 0.024 | 1 |
|  | 80 | WET | 5 | 400 | 168 | 165 | 218 | 14 | 3 | 0.958 | 0.014 | 0.982 | 0.060 | 0.981 |
|  |  | DRY | 5 | 400 | 50 | 50 | 347 | 3 | 0 | 0.993 | 0 | 1 | 0.009 | 1 |
| SVM-9 | 20 | WET | 5 | 100 | 54 | 54 | 19 | 7 | 0 | 0.913 | 0 | 1 | 0.269 | 1 |
|  |  | DRY | 5 | 100 | 30 | 30 | 64 | 6 | 0 | 0.940 | 0 | 1 | 0.086 | 1 |
|  | 50 | WET | 5 | 250 | 138 | 138 | 107 | 5 | 0 | 0.980 | 0 | 1 | 0.045 | 1 |
|  |  | DRY | 5 | 250 | 56 | 56 | 190 | 4 | 0 | 0.984 | 0 | 1 | 0.021 | 1 |
|  | 80 | WET | 5 | 400 | 148 | 147 | 243 | 9 | 1 | 0.975 | 0.004 | 0.993 | 0.036 | 0.993 |
|  |  | DRY | 5 | 400 | 64 | 61 | 334 | 2 | 3 | 0.988 | 0.009 | 0.953 | 0.006 | 0.953 |
| SVM-10 | 20 | WET | 5 | 100 | 70 | 69 | 28 | 2 | 1 | 0.970 | 0.034 | 0.986 | 0.067 | 0.985 |
|  |  | DRY | 5 | 100 | 35 | 32 | 63 | 2 | 3 | 0.950 | 0.045 | 0.914 | 0.031 | 0.912 |
|  | 50 | WET | 5 | 250 | 146 | 139 | 103 | 1 | 7 | 0.968 | 0.064 | 0.952 | 0.010 | 0.952 |
|  |  | DRY | 5 | 250 | 55 | 48 | 192 | 3 | 7 | 0.960 | 0.035 | 0.873 | 0.015 | 0.871 |
|  | 80 | WET | 5 | 400 | 157 | 153 | 236 | 7 | 4 | 0.973 | 0.017 | 0.975 | 0.029 | 0.974 |
|  |  | DRY | 5 | 400 | 63 | 57 | 336 | 1 | 6 | 0.983 | 0.018 | 0.905 | 0.003 | 0.904 |
| ***AVERAGE VALUES on all SVMs***  ***(Mean±SD)*** | *20* | *WET* | *5* | *100* | *68.2±*  *7.6* | *67.1±*  *7.1* | *24.6±*  *5.6* | *5.2±*  *2.4* | *1.1±*  *1.1* | *0.935±*  *0.021* | *0.043±*  *0.042* | *0.985±*  *0.016* | *0.178±*  *0.083* | *0.983±*  *0.018* |
|  |  | *DRY* | *5* | *100* | *31.0±*  *8.2* | *30.0±*  *7.5* | *65.1±*  *8.4* | *3.9±*  *1.9* | *1.0±*  *1.2* | *0.951±*  *0.019* | *0.016±*  *0.021* | *0.971±*  *0.034* | *0.057±*  *0.03* | *0.969±*  *0.036* |
|  | *50* | *WET* | *5* | *250* | *147.7±*  *12.1* | *145.9±*  *12.4* | *97.5±*  *12.1* | *4.8±*  *1.9* | *1.8±*  *2.4* | *0.974±*  *0.007* | *0.017±*  *0.022* | *0.988±*  *0.017* | *0.048±*  *0.019* | *0.987±*  *0.017* |
|  |  | *DRY* | *5* | *250* | *52.2±*  *12.2* | *49.7±*  *10.8* | *194.4±*  *11.7* | *3.4±*  *1.4* | *2.5±*  *3.5* | *0.976±*  *0.01* | *0.013±*  *0.018* | *0.957±*  *0.06* | *0.017±*  *0.007* | *0.957±*  *0.06* |
|  | *80* | *WET* | *5* | *400* | *159.5±*  *12.3* | *157.9±*  *12.8* | *232.1±*  *13.9* | *8.4±*  *3.0* | *1.6±*  *1.4* | *0.975±*  *0.008* | *0.007±*  *0.006* | *0.990±*  *0.009* | *0.035±*  *0.014* | *0.989±*  *0.009* |
|  |  | *DRY* | *5* | *400* | *60.4±*  *12.4* | *56.8±*  *10.7* | *338.5±*  *12.3* | *1.1±*  *1.0* | *3.6±*  *2.8* | *0.988±*  *0.007* | *0.011±*  *0.008* | *0.944±*  *0.038* | *0.003±*  *0.003* | *0.943±*  *0.038* |
