# Supplementary material for: A new ICA-based fingerprint method for the automatic removal of physiological artifacts from EEG recordings
Source: PeerJ. 2018 Feb 23;6:e4380. doi: 10.7717/peerj.4380 (PMC5826009; doi:10.7717/peerj.4380)
Supplement: Table S5 — The statistical performance of the individual SVM classifiers trained to classify ICs containing cardiac interference is reported separately for the wet and dry testing EEG datasets and for the three decomposition levels (20, 50 and 80 ICs). [file peerj-06-4380-s006.docx]

| **Cardiac interference SVM classifiers performance** | | | | | | | | | | | | | | |
| --- | --- | --- | --- | --- | --- | --- | --- | --- | --- | --- | --- | --- | --- | --- |
| **Cardiac interference** | **N. of ICs per dataset** | **Electrode type** | **N. of datasets** | **Total N. of ICs** | **Total N. of artifactual ICs** | **True positive** | **True negative** | **False positive** | **False negative** | **Accuracy** | **FOR** | **HR** | **FAR (*g*)** | **Sensitivity *p*** |
| SVM-1 | 20 | WET | 5 | 100 | 5 | 1 | 95 | 0 | 4 | 0.960 | 0.040 | 0.200 | 0 | 0.200 |
|  |  | DRY | 5 | 100 | 10 | 0 | 89 | 10 | 10 | 0.890 | 0.101 | 0 | 0.011 | -0.011 |
|  | 50 | WET | 5 | 250 | 5 | 0 | 245 | 0 | 5 | 0.980 | 0.020 | 0 | 0 | 0 |
|  |  | DRY | 5 | 250 | 6 | 0 | 244 | 0 | 6 | 0.976 | 0.024 | 0 | 0 | 0 |
|  | 80 | WET | 5 | 400 | 5 | 1 | 395 | 0 | 4 | 0.990 | 0.010 | 0.200 | 0 | 0.200 |
|  |  | DRY | 5 | 400 | 6 | 1 | 394 | 0 | 5 | 0.988 | 0.013 | 0.167 | 0 | 0.167 |
| SVM-2 | 20 | WET | 5 | 100 | 4 | 1 | 96 | 0 | 3 | 0.970 | 0.030 | 0.250 | 0 | 0.250 |
|  |  | DRY | 5 | 100 | 7 | 0 | 93 | 0 | 7 | 0.930 | 0.070 | 0 | 0 | 0 |
|  | 50 | WET | 5 | 250 | 5 | 1 | 245 | 0 | 4 | 0.984 | 0.016 | 0.200 | 0 | 0.200 |
|  |  | DRY | 5 | 250 | 5 | 0 | 245 | 0 | 5 | 0.980 | 0.020 | 0 | 0 | 0 |
|  | 80 | WET | 5 | 400 | 5 | 1 | 395 | 0 | 4 | 0.990 | 0.010 | 0.200 | 0 | 0.200 |
|  |  | DRY | 5 | 400 | 5 | 1 | 395 | 0 | 4 | 0.990 | 0.010 | 0.200 | 0 | 0.200 |
| SVM-3 | 20 | WET | 5 | 100 | 5 | 0 | 95 | 0 | 5 | 0.950 | 0.050 | 0 | 0 | 0 |
|  |  | DRY | 5 | 100 | 11 | 0 | 89 | 0 | 11 | 0.890 | 0.110 | 0 | 0 | 0 |
|  | 50 | WET | 5 | 250 | 5 | 0 | 245 | 0 | 5 | 0.980 | 0.020 | 0 | 0 | 0 |
|  |  | DRY | 5 | 250 | 5 | 1 | 245 | 0 | 4 | 0.984 | 0.016 | 0.200 | 0 | 0.200 |
|  | 80 | WET | 5 | 400 | 5 | 0 | 395 | 0 | 5 | 0.988 | 0.013 | 0 | 0 | 0 |
|  |  | DRY | 5 | 400 | 5 | 1 | 395 | 0 | 4 | 0.990 | 0.010 | 0.200 | 0 | 0.200 |
| SVM-4 | 20 | WET | 5 | 100 | 4 | 0 | 96 | 0 | 4 | 0.960 | 0.040 | 0 | 0 | 0 |
|  |  | DRY | 5 | 100 | 4 | 0 | 96 | 0 | 4 | 0.960 | 0.040 | 0 | 0 | 0 |
|  | 50 | WET | 5 | 250 | 5 | 0 | 245 | 0 | 5 | 0.980 | 0.020 | 0 | 0 | 0 |
|  |  | DRY | 5 | 250 | 6 | 1 | 244 | 0 | 5 | 0.980 | 0.020 | 0.167 | 0 | 0.167 |
|  | 80 | WET | 5 | 400 | 5 | 3 | 395 | 0 | 2 | 0.995 | 0.005 | 0.600 | 0 | 0.600 |
|  |  | DRY | 5 | 400 | 6 | 3 | 393 | 1 | 3 | 0.990 | 0.008 | 0.500 | 0.003 | 0.499 |
| SVM-5 | 20 | WET | 5 | 100 | 4 | 0 | 96 | 0 | 4 | 0.960 | 0.040 | 0 | 0 | 0 |
|  |  | DRY | 5 | 100 | 8 | 0 | 92 | 0 | 8 | 0.920 | 0.080 | 0 | 0 | 0 |
|  | 50 | WET | 5 | 250 | 5 | 1 | 245 | 0 | 4 | 0.984 | 0.016 | 0.200 | 0 | 0.200 |
|  |  | DRY | 5 | 250 | 5 | 2 | 245 | 0 | 3 | 0.988 | 0.012 | 0.400 | 0 | 0.400 |
|  | 80 | WET | 5 | 400 | 5 | 1 | 395 | 0 | 4 | 0.990 | 0.010 | 0.200 | 0 | 0.200 |
|  |  | DRY | 5 | 400 | 5 | 3 | 395 | 0 | 2 | 0.995 | 0.005 | 0.600 | 0 | 0.600 |
| SVM-6 | 20 | WET | 5 | 100 | 5 | 1 | 95 | 0 | 4 | 0.960 | 0.040 | 0.200 | 0 | 0.200 |
|  |  | DRY | 5 | 100 | 7 | 0 | 93 | 0 | 7 | 0.930 | 0.070 | 0 | 0 | 0 |
|  | 50 | WET | 5 | 250 | 5 | 1 | 245 | 0 | 4 | 0.984 | 0.016 | 0.200 | 0 | 0.200 |
|  |  | DRY | 5 | 250 | 5 | 0 | 245 | 0 | 5 | 0.980 | 0.020 | 0 | 0 | 0 |
|  | 80 | WET | 5 | 400 | 5 | 1 | 395 | 0 | 4 | 0.990 | 0.010 | 0.200 | 0 | 0.200 |
|  |  | DRY | 5 | 400 | 5 | 0 | 395 | 0 | 5 | 0.988 | 0.013 | 0 | 0 | 0 |
| SVM-7 | 20 | WET | 5 | 100 | 4 | 1 | 96 | 0 | 3 | 0.970 | 0.030 | 0.250 | 0 | 0.250 |
|  |  | DRY | 5 | 100 | 3 | 2 | 95 | 2 | 1 | 0.970 | 0.010 | 0.667 | 0.021 | 0.660 |
|  | 50 | WET | 5 | 250 | 5 | 3 | 243 | 2 | 2 | 0.984 | 0.008 | 0.600 | 0.008 | 0.597 |
|  |  | DRY | 5 | 250 | 5 | 1 | 243 | 2 | 4 | 0.976 | 0.016 | 0.200 | 0.008 | 0.193 |
|  | 80 | WET | 5 | 400 | 5 | 4 | 395 | 0 | 1 | 0.998 | 0.003 | 0.800 | 0 | 0.800 |
|  |  | DRY | 5 | 400 | 5 | 2 | 392 | 3 | 3 | 0.985 | 0.008 | 0.400 | 0.008 | 0.395 |
| SVM-8 | 20 | WET | 5 | 100 | 5 | 0 | 95 | 0 | 5 | 0.950 | 0.050 | 0 | 0 | 0 |
|  |  | DRY | 5 | 100 | 9 | 0 | 91 | 0 | 9 | 0.910 | 0.090 | 0 | 0 | 0 |
|  | 50 | WET | 5 | 250 | 5 | 0 | 245 | 0 | 5 | 0.980 | 0.020 | 0 | 0 | 0 |
|  |  | DRY | 5 | 250 | 6 | 0 | 244 | 0 | 6 | 0.976 | 0.024 | 0 | 0 | 0 |
|  | 80 | WET | 5 | 400 | 5 | 0 | 395 | 0 | 5 | 0.988 | 0.013 | 0 | 0 | 0 |
|  |  | DRY | 5 | 400 | 6 | 2 | 394 | 0 | 4 | 0.990 | 0.010 | 0.333 | 0 | 0.333 |
| SVM-9 | 20 | WET | 5 | 100 | 5 | 2 | 95 | 0 | 3 | 0.970 | 0.031 | 0.400 | 0 | 0.400 |
|  |  | DRY | 5 | 100 | 8 | 0 | 92 | 0 | 8 | 0.920 | 0.080 | 0 | 0 | 0 |
|  | 50 | WET | 5 | 250 | 5 | 2 | 245 | 0 | 3 | 0.988 | 0.012 | 0.400 | 0 | 0.400 |
|  |  | DRY | 5 | 250 | 6 | 1 | 244 | 0 | 5 | 0.980 | 0.020 | 0.167 | 0 | 0.167 |
|  | 80 | WET | 5 | 400 | 5 | 2 | 395 | 0 | 3 | 0.993 | 0.008 | 0.400 | 0 | 0.400 |
|  |  | DRY | 5 | 400 | 6 | 2 | 394 | 0 | 4 | 0.990 | 0.010 | 0.333 | 0 | 0.333 |
| SVM-10 | 20 | WET | 5 | 100 | 5 | 1 | 95 | 0 | 4 | 0.960 | 0.040 | 0.200 | 0 | 0.200 |
|  |  | DRY | 5 | 100 | 3 | 0 | 97 | 0 | 3 | 0.970 | 0.030 | 0 | 0 | 0 |
|  | 50 | WET | 5 | 250 | 5 | 1 | 245 | 0 | 4 | 0.984 | 0.016 | 0.200 | 0 | 0.200 |
|  |  | DRY | 5 | 250 | 6 | 0 | 244 | 0 | 6 | 0.976 | 0.024 | 0 | 0 | 0 |
|  | 80 | WET | 5 | 400 | 5 | 3 | 395 | 0 | 2 | 0.995 | 0.005 | 0.600 | 0 | 0.600 |
|  |  | DRY | 5 | 400 | 6 | 2 | 394 | 0 | 4 | 0.990 | 0.010 | 0.333 | 0 | 0.333 |
| ***AVERAGE VALUES on all SVMs***  ***(Mean±SD)*** | *20* | *WET* | *5* | *100* | *4.6±*  *0.5* | *0.7±*  *0.7* | *95.4±*  *0.5* | *0* | *3.9±*  *0.7* | *0.961±*  *0.007* | *0.039±*  *0.007* | *0.150±*  *0.141* | *0* | *0.150±*  *0.141* |
|  |  | *DRY* | *5* | *100* | *7.0±*  *2.8* | *0.2±*  *0.6* | *92.7±*  *2.7* | *0.3±*  *0.7* | *6.8±*  *3.2* | *0.929±*  *0.030* | *0.068±*  *0.032* | *0.067±*  *0.211* | *0.003±*  *0.007* | *0.065±*  *0.209* |
|  | *50* | *WET* | *5* | *250* | *5* | *0.9±*  *1.0* | *244.8±*  *0.6* | *0.2±*  *0.6* | *4.1±*  *1.0* | *0.983±*  *0.003* | *0.016±*  *0.004* | *0.180±*  *0.199* | *0.001±*  *0.003* | *0.180±*  *0.198* |
|  |  | *DRY* | *5* | *250* | *5.5±*  *0.5* | *0.6±*  *0.7* | *244.3±*  *0.7* | *0.2±*  *0.6* | *4.9±*  *1.0* | *0.980±*  *0.004* | *0.020±*  *0.004* | *0.113±*  *0.136* | *0.001±*  *0.003* | *0.113±*  *0.136* |
|  | *80* | *WET* | *5* | *400* | *5* | *1.6±*  *1.3* | *395* | *0* | *3.4±*  *1.3* | *0.992±*  *0.003* | *0.009±*  *0.003* | *0.320±*  *0.270* | *0* | *0.320±*  *0.270* |
|  |  | *DRY* | *5* | *400* | *5.5±*  *0.5* | *1.7±*  *0.9* | *394.1±*  *1.0* | *0.4±*  *1.0* | *3.8±*  *0.9* | *0.990±*  *0.003* | *0.010±*  *0.002* | *0.307±*  *0.173* | *0.001±*  *0.002* | *0.306±*  *0.173* |
