# Supplementary material for: A new ICA-based fingerprint method for the automatic removal of physiological artifacts from EEG recordings
Source: PeerJ. 2018 Feb 23;6:e4380. doi: 10.7717/peerj.4380 (PMC5826009; doi:10.7717/peerj.4380)
Supplement: Table S6 — Descriptive statistics of the individual features of the reference and non-artifactual fingerprints for eyeblinks are given separately for wet and dry EEG datasets. [file peerj-06-4380-s007.docx]

| **Eyeblinks: Descriptive Statistics of Fingerprint Features** | | | | | | | | | | | | | | | | | | | | |
| --- | --- | --- | --- | --- | --- | --- | --- | --- | --- | --- | --- | --- | --- | --- | --- | --- | --- | --- | --- | --- |
|  | **Reference fingerprint** | | | | | | | | | | **Non-artifactual fingerprint** | | | | | | | | | |
|  | **Wet** | | | | | **Dry** | | | | | **Wet** | | | | | **Dry** | | | | |
| **Features** | Mean | SD | Median | Interquartile range | 95% percentile | Mean | SD | Median | Interquartile range | 95% percentile | Mean | SD | Median | Interquartile range | 95% percentile | Mean | SD | Median | Interquartile range | 95% percentile |
| K | 1 | 0 | 1 | 0 | 1 | 0.982 | 0.049 | 1 | 0 | 1 | 0.029 | 0.074 | 0.005 | 0.020 | 0.146 | 0.018 | 0.048 | 0.006 | 0.012 | 0.061 |
| MEV | 0.056 | 0.024 | 0.050 | 0.038 | 0.085 | 0.057 | 0.028 | 0.043 | 0.025 | 0.101 | 0.154 | 0.151 | 0.119 | 0.097 | 0.368 | 0.186 | 0.227 | 0.075 | 0.180 | 0.703 |
| SAD | 0.963 | 0.080 | 1 | 0.016 | 1 | 0.694 | 0.476 | 0.975 | 0.558 | 1 | 0.116 | 0.239 | 0 | 0.024 | 0.708 | 0.059 | 0.181 | 0 | 0 | 0.520 |
| SED | 0 | 0 | 0 | 0 | 0 | 0.218 | 0.375 | 0 | 0.340 | 0.794 | 0.185 | 0.248 | 0 | 0.367 | 0.674 | 0.122 | 0.210 | 0 | 0.199 | 0.554 |
| PSD Delta | 0.940 | 0.040 | 0.946 | 0.064 | 0.982 | 0.921 | 0.025 | 0.913 | 0.035 | 0.954 | 0.541 | 0.187 | 0.580 | 0.211 | 0.782 | 0.530 | 0.231 | 0.446 | 0.342 | 0.919 |
| PSD Theta | 0.048 | 0.032 | 0.045 | 0.054 | 0.082 | 0.055 | 0.023 | 0.059 | 0.020 | 0.079 | 0.079 | 0.039 | 0.073 | 0.030 | 0.149 | 0.085 | 0.045 | 0.098 | 0.079 | 0.138 |
| PSD Alpha | 0.006 | 0.004 | 0.005 | 0.004 | 0.012 | 0.008 | 0.004 | 0.007 | 0.005 | 0.014 | 0.069 | 0.078 | 0.049 | 0.037 | 0.177 | 0.070 | 0.062 | 0.070 | 0.052 | 0.125 |
| PSD Beta | 0.003 | 0.002 | 0.002 | 0.003 | 0.007 | 0.010 | 0.005 | 0.009 | 0.004 | 0.017 | 0.145 | 0.058 | 0.136 | 0.059 | 0.261 | 0.161 | 0.085 | 0.197 | 0.144 | 0.264 |
| PSD Gamma | 0.003 | 0.002 | 0.002 | 0.002 | 0.006 | 0.006 | 0.002 | 0.007 | 0.002 | 0.009 | 0.165 | 0.133 | 0.134 | 0.098 | 0.522 | 0.154 | 0.134 | 0.123 | 0.121 | 0.496 |
| CIF | 0 | 0 | 0 | 0 | 0 | 0 | 0 | 0 | 0 | 0 | 0.045 | 0.146 | 0 | 0 | 0.480 | 0.114 | 0.231 | 0 | 0 | 0.632 |
| MIF | 0 | 0 | 0 | 0 | 0 | 0 | 0 | 0 | 0 | 0 | 0.056 | 0.199 | 0 | 0 | 0.710 | 0.060 | 0.204 | 0 | 0 | 0.711 |
| CORR EyeBlink | 0.806 | 0.026 | 0.804 | 0.031 | 0.837 | 0.792 | 0.020 | 0.793 | 0.017 | 0.814 | 0.634 | 0.198 | 0.696 | 0.020 | 0.716 | 0.652 | 0.180 | 0.701 | 0.030 | 0.731 |
| CORR EyeMov | 0.727 | 0.009 | 0.727 | 0.013 | 0.737 | 0.720 | 0.009 | 0.718 | 0.011 | 0.730 | 0.691 | 0.146 | 0.727 | 0.018 | 0.740 | 0.693 | 0.132 | 0.713 | 0.040 | 0.759 |
| EF | 0.965 | 0.025 | 0.962 | 0.015 | 0.995 | 0.959 | 0.042 | 0.963 | 0.048 | 1 | 0.024 | 0.102 | 0 | 0 | 0.247 | 0.011 | 0.068 | 0 | 0 | 0 |
