# Supplementary material for: A new ICA-based fingerprint method for the automatic removal of physiological artifacts from EEG recordings
Source: PeerJ. 2018 Feb 23;6:e4380. doi: 10.7717/peerj.4380 (PMC5826009; doi:10.7717/peerj.4380)
Supplement: Table S7 — Descriptive statistics of the individual features of the reference and non-artifactual fingerprints for eye movements are given separately for wet and dry EEG datasets. [file peerj-06-4380-s008.docx]

| **Eye movements: Descriptive Statistics of Fingerprint Features** | | | | | | | | | | | | | | | | | | | | |
| --- | --- | --- | --- | --- | --- | --- | --- | --- | --- | --- | --- | --- | --- | --- | --- | --- | --- | --- | --- | --- |
|  | **Reference fingerprint** | | | | | | | | | | **Non-artifactual fingerprint** | | | | | | | | | |
|  | **Wet** | | | | | **Dry** | | | | | **Wet** | | | | | **Dry** | | | | |
| **Features** | Mean | SD | Median | Interquartile range | 95% percentile | Mean | SD | Median | Interquartile range | 95% percentile | Mean | SD | Median | Interquartile range | 95% percentile | Mean | SD | Median | Interquartile range | 95% percentile |
| K | 0.050 | 0.131 | 0.014 | 0.029 | 0.169 | 0.076 | 0.094 | 0.044 | 0.061 | 0.250 | 0.104 | 0.186 | 0.033 | 0.090 | 0.495 | 0.101 | 0.168 | 0.048 | 0.069 | 0.375 |
| MEV | 0.239 | 0.102 | 0.230 | 0.136 | 0.395 | 0.177 | 0.099 | 0.157 | 0.081 | 0.353 | 0.215 | 0.183 | 0.173 | 0.175 | 0.573 | 0.189 | 0.171 | 0.141 | 0.091 | 0.556 |
| SAD | 0 | 0 | 0 | 0 | 0 | 0 | 0 | 0 | 0 | 0 | 0.118 | 0.255 | 0 | 0 | 0.831 | 0.080 | 0.225 | 0 | 0 | 0.685 |
| SED | 0.839 | 0.119 | 0.865 | 0.189 | 0.989 | 0.821 | 0.125 | 0.833 | 0.199 | 0.995 | 0.249 | 0.247 | 0.218 | 0.450 | 0.691 | 0.187 | 0.247 | 0 | 0.344 | 0.670 |
| PSD Delta | 0.540 | 0.100 | 0.537 | 0.141 | 0.689 | 0.452 | 0.072 | 0.444 | 0.069 | 0.574 | 0.449 | 0.212 | 0.493 | 0.289 | 0.783 | 0.459 | 0.136 | 0.446 | 0.100 | 0.700 |
| PSD Theta | 0.079 | 0.017 | 0.076 | 0.019 | 0.107 | 0.113 | 0.019 | 0.116 | 0.018 | 0.139 | 0.078 | 0.039 | 0.078 | 0.039 | 0.153 | 0.111 | 0.029 | 0.117 | 0.029 | 0.147 |
| PSD Alpha | 0.059 | 0.024 | 0.054 | 0.025 | 0.091 | 0.072 | 0.014 | 0.072 | 0.014 | 0.091 | 0.078 | 0.081 | 0.057 | 0.047 | 0.236 | 0.077 | 0.027 | 0.077 | 0.024 | 0.111 |
| PSD Beta | 0.146 | 0.036 | 0.146 | 0.052 | 0.206 | 0.201 | 0.029 | 0.205 | 0.026 | 0.238 | 0.169 | 0.070 | 0.160 | 0.077 | 0.308 | 0.199 | 0.056 | 0.208 | 0.054 | 0.258 |
| PSD Gamma | 0.176 | 0.067 | 0.159 | 0.094 | 0.294 | 0.163 | 0.048 | 0.161 | 0.036 | 0.239 | 0.226 | 0.173 | 0.166 | 0.154 | 0.633 | 0.154 | 0.090 | 0.134 | 0.070 | 0.381 |
| CIF | 0.076 | 0.195 | 0 | 0 | 0.609 | 0.189 | 0.278 | 0 | 0.555 | 0.644 | 0.086 | 0.200 | 0 | 0 | 0.590 | 0.138 | 0.247 | 0 | 0 | 0.624 |
| MIF | 0.003 | 0.044 | 0 | 0 | 0 | 0.006 | 0.058 | 0 | 0 | 0 | 0.132 | 0.288 | 0 | 0 | 0.823 | 0.037 | 0.151 | 0 | 0 | 0.574 |
| CORR Eyeblink | 0.691 | 0.052 | 0.697 | 0.020 | 0.719 | 0.669 | 0.164 | 0.700 | 0.041 | 0.759 | 0.529 | 0.298 | 0.688 | 0.047 | 0.721 | 0.628 | 0.223 | 0.698 | 0.041 | 0.762 |
| CORR EyeMov | 0.729 | 0.016 | 0.734 | 0.015 | 0.747 | 0.716 | 0.025 | 0.715 | 0.032 | 0.754 | 0.592 | 0.278 | 0.724 | 0.059 | 0.750 | 0.679 | 0.156 | 0.714 | 0.039 | 0.758 |
| EF | 0.002 | 0.029 | 0 | 0 | 0 | 0.008 | 0.045 | 0 | 0 | 0 | 0.066 | 0.186 | 0 | 0 | 0.584 | 0.037 | 0.124 | 0 | 0 | 0.333 |
