# Supplementary material for: A new ICA-based fingerprint method for the automatic removal of physiological artifacts from EEG recordings
Source: PeerJ. 2018 Feb 23;6:e4380. doi: 10.7717/peerj.4380 (PMC5826009; doi:10.7717/peerj.4380)
Supplement: Table S8 — Descriptive statistics of the individual features of the reference and non-artifactual fingerprints for myogenic artifacts are given separately for wet and dry EEG datasets. [file peerj-06-4380-s009.docx]

| **Myogenic artifacts: Descriptive Statistics of Fingerprint Features** | | | | | | | | | | | | | | | | | | | | |
| --- | --- | --- | --- | --- | --- | --- | --- | --- | --- | --- | --- | --- | --- | --- | --- | --- | --- | --- | --- | --- |
|  | **Reference fingerprint** | | | | | | | | | | **Non-artifactual fingerprint** | | | | | | | | | |
|  | **Wet** | | | | | **Dry** | | | | | **Wet** | | | | | **Dry** | | | | |
| **Features** | Mean | SD | Median | Interquartile range | 95% percentile | Mean | SD | Median | Interquartile range | 95% percentile | Mean | SD | Median | Interquartile range | 95% percentile | Mean | SD | Median | Interquartile range | 95% percentile |
| K | 0.354 | 0.198 | 0.340 | 0.194 | 0.767 | 0.365 | 0.202 | 0.321 | 0.165 | 0.995 | 0.046 | 0.086 | 0.028 | 0.033 | 0.117 | 0.061 | 0.112 | 0.030 | 0.040 | 0.218 |
| MEV | 0.233 | 0.126 | 0.207 | 0.103 | 0.429 | 0.164 | 0.085 | 0.141 | 0.053 | 0.300 | 0.454 | 0.200 | 0.456 | 0.290 | 0.791 | 0.378 | 0.223 | 0.321 | 0.260 | 0.841 |
| SAD | 0.125 | 0.228 | 0 | 0.205 | 0.576 | 0.140 | 0.254 | 0 | 0.143 | 0.744 | 0.181 | 0.283 | 0 | 0.327 | 0.763 | 0.130 | 0.245 | 0 | 0.150 | 0.656 |
| SED | 0.197 | 0.265 | 0.060 | 0.355 | 0.761 | 0.300 | 0.322 | 0.238 | 0.531 | 0.926 | 0.218 | 0.299 | 0 | 0.441 | 0.866 | 0.228 | 0.264 | 0 | 0.510 | 0.651 |
| PSD Delta | 0.070 | 0.070 | 0.045 | 0.056 | 0.207 | 0.124 | 0.112 | 0.086 | 0.160 | 0.360 | 0.541 | 0.157 | 0.565 | 0.171 | 0.753 | 0.610 | 0.152 | 0.596 | 0.191 | 0.879 |
| PSD Theta | 0.009 | 0.008 | 0.006 | 0.008 | 0.027 | 0.022 | 0.021 | 0.014 | 0.029 | 0.069 | 0.062 | 0.024 | 0.059 | 0.027 | 0.111 | 0.083 | 0.029 | 0.084 | 0.040 | 0.129 |
| PSD Alpha | 0.009 | 0.008 | 0.006 | 0.006 | 0.024 | 0.015 | 0.014 | 0.010 | 0.018 | 0.039 | 0.058 | 0.055 | 0.045 | 0.034 | 0.146 | 0.056 | 0.035 | 0.056 | 0.033 | 0.092 |
| PSD Beta | 0.158 | 0.032 | 0.151 | 0.025 | 0.217 | 0.138 | 0.025 | 0.138 | 0.034 | 0.182 | 0.125 | 0.044 | 0.120 | 0.048 | 0.200 | 0.111 | 0.050 | 0.113 | 0.074 | 0.186 |
| PSD Gamma | 0.754 | 0.084 | 0.781 | 0.089 | 0.829 | 0.701 | 0.135 | 0.751 | 0.217 | 0.839 | 0.213 | 0.104 | 0.185 | 0.112 | 0.424 | 0.140 | 0.089 | 0.120 | 0.074 | 0.332 |
| CIF | 0.046 | 0.130 | 0 | 0 | 0.413 | 0.032 | 0.107 | 0 | 0 | 0.336 | 0.077 | 0.197 | 0 | 0 | 0.623 | 0.130 | 0.246 | 0 | 0 | 0.650 |
| MIF | 0.884 | 0.084 | 0.911 | 0.080 | 0.959 | 0.817 | 0.148 | 0.863 | 0.247 | 0.970 | 0.046 | 0.162 | 0 | 0 | 0.552 | 0.009 | 0.074 | 0 | 0 | 0 |
| CORR Eyeblink | 0.099 | 0.241 | 0 | 0 | 0.681 | 0.292 | 0.349 | 0 | 0.688 | 0.730 | 0.630 | 0.224 | 0.700 | 0.042 | 0.757 | 0.703 | 0.123 | 0.718 | 0.049 | 0.793 |
| CORR EyeMov | 0.106 | 0.249 | 0 | 0 | 0.701 | 0.390 | 0.345 | 0.661 | 0.686 | 0.720 | 0.653 | 0.241 | 0.744 | 0.055 | 0.780 | 0.661 | 0.203 | 0.719 | 0.050 | 0.770 |
| EF | 0.154 | 0.270 | 0 | 0.222 | 0.797 | 0.361 | 0.336 | 0.313 | 0.635 | 0.905 | 0.004 | 0.042 | 0 | 0 | 0 | 0.016 | 0.104 | 0 | 0 | 0 |
