# Supplementary material for: A new ICA-based fingerprint method for the automatic removal of physiological artifacts from EEG recordings
Source: PeerJ. 2018 Feb 23;6:e4380. doi: 10.7717/peerj.4380 (PMC5826009; doi:10.7717/peerj.4380)
Supplement: Table S9 — Descriptive statistics of the individual features of the reference and non-artifactual fingerprints for cardiac interference are given separately for wet and dry EEG datasets. [file peerj-06-4380-s010.docx]

| **Cardiac Interference: Descriptive Statistics of Fingerprint Features** | | | | | | | | | | | | | | | | | | | | |
| --- | --- | --- | --- | --- | --- | --- | --- | --- | --- | --- | --- | --- | --- | --- | --- | --- | --- | --- | --- | --- |
|  | **Reference fingerprint** | | | | | | | | | | **Non-artifactual fingerprint** | | | | | | | | | |
|  | **Wet** | | | | | **Dry** | | | | | **Wet** | | | | | **Dry** | | | | |
| **Features** | Mean | SD | Median | Interquartile range | 95% percentile | Mean | SD | Median | Interquartile range | 95% percentile | Mean | SD | Median | Interquartile range | 95% percentile | Mean | SD | Median | Interquartile range | 95% percentile |
| K | 0.135 | 0.070 | 0.122 | 0.065 | 0.248 | 0.077 | 0.040 | 0.067 | 0.049 | 0.137 | 0.040 | 0.130 | 0.005 | 0.025 | 0.168 | 0.038 | 0.132 | 0.007 | 0.016 | 0.107 |
| MEV | 0.040 | 0.012 | 0.037 | 0.017 | 0.055 | 0.027 | 0.002 | 0.027 | 0.001 | 0.031 | 0.170 | 0.161 | 0.121 | 0.144 | 0.445 | 0.113 | 0.170 | 0.055 | 0.061 | 0.474 |
| SAD | 0 | 0 | 0 | 0 | 0 | 0.062 | 0.163 | 0 | 0 | 0.302 | 0.119 | 0.244 | 0 | 0.065 | 0.704 | 0.069 | 0.193 | 0 | 0 | 0.546 |
| SED | 0 | 0 | 0 | 0 | 0 | 0 | 0 | 0 | 0 | 0 | 0.234 | 0.294 | 0 | 0.460 | 0.828 | 0.111 | 0.195 | 0 | 0.191 | 0.475 |
| PSD Delta | 0.284 | 0.062 | 0.285 | 0.094 | 0.354 | 0.632 | 0.163 | 0.676 | 0.113 | 0.765 | 0.513 | 0.208 | 0.565 | 0.245 | 0.769 | 0.444 | 0.178 | 0.428 | 0.137 | 0.776 |
| PSD Theta | 0.153 | 0.023 | 0.146 | 0.029 | 0.186 | 0.128 | 0.033 | 0.132 | 0.047 | 0.166 | 0.068 | 0.034 | 0.066 | 0.030 | 0.121 | 0.095 | 0.035 | 0.103 | 0.033 | 0.133 |
| PSD Alpha | 0.118 | 0.025 | 0.118 | 0.034 | 0.154 | 0.039 | 0.021 | 0.030 | 0.013 | 0.071 | 0.060 | 0.070 | 0.044 | 0.026 | 0.147 | 0.067 | 0.049 | 0.068 | 0.028 | 0.097 |
| PSD Beta | 0.266 | 0.027 | 0.258 | 0.026 | 0.307 | 0.101 | 0.069 | 0.084 | 0.021 | 0.205 | 0.152 | 0.065 | 0.139 | 0.062 | 0.273 | 0.194 | 0.065 | 0.204 | 0.063 | 0.287 |
| PSD Gamma | 0.179 | 0.050 | 0.175 | 0.084 | 0.239 | 0.100 | 0.070 | 0.076 | 0.062 | 0.208 | 0.208 | 0.176 | 0.154 | 0.139 | 0.623 | 0.199 | 0.138 | 0.167 | 0.087 | 0.548 |
| CIF | 0.644 | 0.046 | 0.634 | 0.059 | 0.712 | 0.682 | 0.078 | 0.684 | 0.079 | 0.789 | 0.057 | 0.160 | 0 | 0 | 0.529 | 0.155 | 0.258 | 0 | 0.427 | 0.647 |
| MIF | 0 | 0 | 0 | 0 | 0 | 0 | 0 | 0 | 0 | 0 | 0.113 | 0.272 | 0 | 0 | 0.835 | 0,084 | 0,238 | 0 | 0 | 0,777 |
| CORR Eyeblink | 0.250 | 0.345 | 0 | 0.652 | 0.678 | 0.406 | 0.380 | 0.691 | 0.715 | 0.720 | 0.623 | 0.219 | 0.697 | 0.022 | 0.725 | 0.635 | 0.205 | 0.695 | 0.030 | 0.744 |
| CORR EyeMov | 0.592 | 0.240 | 0.667 | 0.022 | 0.701 | 0.480 | 0.328 | 0.663 | 0.348 | 0.681 | 0.689 | 0.150 | 0.725 | 0.023 | 0.743 | 0.675 | 0.147 | 0.705 | 0.023 | 0.735 |
| EF | 0.149 | 0.165 | 0.106 | 0.302 | 0.350 | 0 | 0 | 0 | 0 | 0 | 0.036 | 0.145 | 0 | 0 | 0.292 | 0.032 | 0.146 | 0 | 0 | 0.259 |
