# Supplementary material for: A new ICA-based fingerprint method for the automatic removal of physiological artifacts from EEG recordings
Source: PeerJ. 2018 Feb 23;6:e4380. doi: 10.7717/peerj.4380 (PMC5826009; doi:10.7717/peerj.4380)
Supplement: Table S10 — Panel 1: For each artifact type, we report the average SNR values of the filtered EEG signals and of the artifact-free EEG signals for the individual datasets used to test the SVM classifier with the best performance. For each dataset, the SNR is calculated on the representative channel, that can vary across datasets. Results are given separately for wet and dry EEG datasets and for each decomposition level (20, 50 and 80 ICs). SNR values are given in dB (Mean ± SD). Panel 2: For each artifact type, we report the average SNR values of the filtered EEG signals and of the artifact-free EEG signals separately for wet and dry EEG datasets and for each decomposition level (20, 50 and 80 ICs). SNR values are given in dB (Mean ± SD). The average relative SNR reduction after artifact removal is given in % (Mean ± SD). [file peerj-06-4380-s011.docx]

| **Panel 1 - Average SNR values for individual EEG datasets** | | | | | | | | | | | | | | | | | | | | | | | | | | |
| --- | --- | --- | --- | --- | --- | --- | --- | --- | --- | --- | --- | --- | --- | --- | --- | --- | --- | --- | --- | --- | --- | --- | --- | --- | --- | --- |
| **Artifact** | | **Electrode type** | **N. of ICs per dataset** | | **Datasets used for the testing of the best SVM** | | | | | | | | | | | | | | | | | | | | | |
| **EyeBlinkS - SVM-6** | | **WET** |  | | dataset-1 | | | dataset-2 | | | | dataset-8 | | | | dataset-9 | | | dataset-10 | | | dataset-11 | | | | |
|  |  |  |  | | channel: R1 | | | channel: L1 | | | | channel: Z1 | | | | channel: L1 | | | channel: R1 | | | channel: R1 | | | | |
|  |  |  |  | | pre | | post | pre | | post | | pre | | post | | pre | post | | pre | | post | pre | | | post | |
|  |  |  | 20 | | 23.7±2.9 | | 4.9±3.0 | 25.3±1.6 | | 6.4±4.8 | | 21.8±2.3 | | 4.8±2.9 | | 23.9±2.8 | 3.4±3.0 | | 24.4±5.7 | | 6.5±2.1 | 20.2±3.0 | | | 4.5±2.6 | |
|  |  |  | 50 | |  |  | 5.2±2.9 |  |  | 6.3±4.7 | |  |  | 4.9±2.7 | |  | 3.8±2.8 | |  |  | 6.6±2.1 |  |  |  | 5.1±2.8 | |
|  |  |  | 80 | |  |  | 4.9±2.5 |  |  | 6.6±4.6 | |  |  | 4.7±2.6 | |  | 3.8±2.6 | |  |  | 6.2±2.3 |  |  |  | 5.3±2.9 | |
|  |  | **DRY** |  | | dataset-1 | | | dataset-4 | | | | dataset-5 | | | | dataset-6 | | | dataset-7 | | | dataset-8 | | | | |
|  |  |  |  | | channel: L1 | | | channel: L1 | | | | channel: LD1 | | | | channel: L1 | | | channel: LD1 | | | channel: L1 | | | | |
|  |  |  |  | | pre | | post | pre | | post | | pre | | post | | pre | post | | pre | | post | pre | | | post | |
|  |  |  | 20 | | 24.0±1.7 | | 7.4±2.4 | 22.2±2.5 | | 3.5±2.2 | | 18.1±2.1 | | 3.3±1.9 | | 11.0±1.2 | 1.4±1.0 | | 18.8±5.1 | | 2.9±2.7 | 22.9±1.2 | | | 3.0±1.4 | |
|  |  |  | 50 | |  |  | 8.8±3.0 |  |  | 3.9±2.0 | |  |  | 3.5±2.1 | |  | 1.8±1.1 | |  |  | 3.7±2.9 |  |  |  | 3.3±2.0 | |
|  |  |  | 80 | |  |  | 8.7±2.9 |  |  | 4.4±2.0 | |  |  | 3.9±2.2 | |  | 1.7±1.1 | |  |  | 5.2±2.8 |  |  |  | 3.4±2.0 | |
| **Eye MovementS - SVM-2** | | **WET** |  | | dataset-3 | | | dataset-4 | | | | dataset-8 | | | | dataset-10 | | |  | | |  | | | | |
|  |  |  |  | | channel: LE1 | | | channel: LE1 | | | | channel: LD2 | | | | channel: LE1 | | |  | | |  | | | | |
|  |  |  |  | | pre | | post | pre | | post | | pre | | post | | pre | post | |  | |  |  | | |  | |
|  |  |  | 20 | | 15.0±3.3 | | 3.4±2.9 | 12.9±1.9 | | 3.1±2.3 | | 15.1±2.8 | | 3.9±2.8 | | 13.8±2.5 | 1.6±1.8 | |  | |  |  | | |  | |
|  |  |  | 50 | |  |  | 4.7±3.2 |  |  | 3.2±1.9 | |  |  | 5.3±2.6 | |  | 3.5±1.7 | |  | |  |  | | |  | |
|  |  |  | 80 | |  |  | 4.3±2.8 |  |  | 4.8±2.4 | |  |  | 4.2±2.2 | |  | 3.1±2.0 | |  | |  |  | | |  | |
|  |  | **DRY** |  | | dataset-2 | | | dataset-3 | | | | dataset-6 | | | | dataset-9 | | |  | | |  | | | | |
|  |  |  |  | | channel: RD2 | | | channel: LD2 | | | | channel: LD2 | | | | channel: LE1 | | |  | | |  | | | | |
|  |  |  |  | | pre | | post | pre | | post | | pre | | post | | pre | post | |  | |  |  | | |  | |
|  |  |  | 20 | | 8.2±2.1 | | 3.5±2.4 | 10.9±2.2 | | 3.8±2.0 | | 12.3±3.2 | | 2.0±2.4 | | 13.3±3.5 | 2.9±3.6 | |  | |  |  | | |  | |
|  |  |  | 50 | |  |  | 4.2±2.2 |  |  | 4.3±3.4 | |  |  | 2.7±1.8 | |  | 3.8±2.5 | |  | |  |  | | |  | |
|  |  |  | 80 | |  |  | 4.6±1.5 |  |  | 3.7±2.5 | |  |  | 3.1±2.4 | |  | 3.6±2.1 | |  | |  | | |  |  | |
| **myogenic artifacts - SVM-8** | | **WET** |  | | dataset-1 | | | dataset-3 | | | | dataset-6 | | | | dataset-8 | | | dataset-9 | | | | |  | | |
|  |  |  |  | | channel: RD2 | | | channel: RC2 | | | | channel: RC4 | | | | channel: RA1 | | | channel: RE1 | | | | |  | | |
|  |  |  |  | | pre | | post | pre | | post | | pre | | post | | pre | post | | pre | | post | | |  | |  |
|  |  |  | 20 | | 29.1±4.1 | | 12.1±3.5 | 33.1±1.0 | | 19.3±2.8 | | 13.4±4.6 | | 4.9±2.4 | | 27.9±2.3 | 13.5±2.3 | | 33.7±2.8 | | 23.3±2.8 | | |  |  | |
|  |  |  | 50 | |  |  | 6.2±2.8 |  |  | 16.6±3.7 | |  |  | 5.8±2.6 | |  | 11.5±2.8 | |  |  | 13.2±3.3 | | |  |  | |
|  |  |  | 80 | |  |  | 10.2±1.4 |  |  | 14.9±2.2 | |  |  | 4.6±2.1 | |  | 11.9±1.9 | |  |  | 10.4±2.9 | | |  |  | |
|  |  | **DRY** |  | | dataset-1 | | | dataset-2 | | | | dataset-6 | | | | dataset-7 | | | dataset-9 | | | | |  | | |
|  |  |  |  | | channel: RB1 | | | channel: LD1 | | | | channel: RC4 | | | | channel: LB1 | | | channel: RC2 | | | | |  | | |
|  |  |  |  | | pre | | post | pre | | post | | pre | | post | | pre | post | | pre | | post | | |  | |  |
|  |  |  | 20 | | 21.7±3.3 | | 17.5±3.2 | 26.2±2.2 | | 13.4±2.5 | | 22.5±5.2 | | 12.8±3.5 | | 15.3±4.0 | 6.6±2.5 | | 13.5±4.2 | | 6.1±3.5 | | |  | |  |
|  |  |  | 50 | |  |  | 18.6±3.4 |  |  | 8.5±2.3 | |  |  | 7.2±1.7 | |  | 5.1±2.5 | |  |  | 5.0±2.2 | | |  | |  |
|  |  |  | 80 | |  |  | 17.8±2.6 |  |  | 8.7±2.2 | |  |  | 5.9±1.6 | |  | 6.1±2.3 | |  |  | 5.5±3.0 | | |  | |  |
| **cardiac interference -SVM-7** | | **WET** |  | | dataset-2 | | | dataset-6 | | | | dataset-7 | | | | dataset-8 | | | dataset-10 | | |  | | | | |
|  |  |  |  | | channel: L13 | | | channel: Z14 | | | | channel: LE4 | | | | channel: LE4 | | | channel: LE4 | | |  | | | | |
|  |  |  |  | | pre | | post | pre | | post | | pre | | post | | pre | post | | pre | | post |  | | |  | |
|  |  |  | 20 | |  | |  | 4.4±3.2 | | 2.7±3.2 | |  | |  | |  |  | |  | |  |  | | |  | |
|  |  |  | 50 | | 3.1±3.0 | | 2.6±3.4 |  |  | 2.3±2.8 | |  | |  | |  |  | | 5.9±1.9 | | 2.6±2.5 |  | | |  | |
|  |  |  | 80 | |  |  | 2.5±3.2 |  | |  | | 7.6±5.0 | | 3.0±3.3 | | 6.2±2.4 | 3.0±2.9 | |  |  | 1.8±2.5 |  | | |  | |
|  |  | **DRY** |  | | dataset-2 | | | dataset-5 | | | | dataset-6 | | | | dataset-8 | | | dataset-9 | | |  | | | | |
|  |  |  |  | | channel: | | | channel: | | | | channel: LL9 | | | | channel: R4 | | | channel: LB3 | | |  | | | | |
|  |  |  |  | | pre | | post | pre | | post | | pre | | post | | pre | post | | pre | | post |  | | |  | |
|  |  |  | 20 | |  | |  |  | |  | | 2.6±2.4 | | 1.1±2.7 | |  |  | | 2.6±2.0 | | 2.3±1.9 |  | | |  | |
|  |  |  | 50 | |  | |  |  | |  | |  |  | 1.3±2.9 | |  |  | |  | |  |  | | |  | |
|  |  |  | 80 | |  | |  |  | |  | |  |  | 1.4±2.5 | | 6.6±4.5 | 5.4±4.3 | |  | |  |  | | |  | |
| **Panel 2 - Average SNR values of EEG signals before and after artifact removal** | | | | | | | | | | | | | | | | | | | | | |  |  |  |  |  |
| **Artifact type** | | | **EEG type** | | **SNR in filtered EEG (dB)** | | | **SNR in artifact-free EEG (dB)** | | | | | | **Relative SNR reduction (%)** | | | | | | | |  |  |  |  |  |
|  |  |  |  |  |  |  |  | **20 ICs** | | **50 ICs** | | **80 ICs** | | **20 ICs** | | | **50 ICs** | | **80 ICs** | | |  |  |  |  |  |
| Eyeblink | | | WET | | 23.2 ± 1.9 | | | 5.1 ± 1.2 | | 5.3 ± 1.0 | | 5.3 ± 1.0 | | 78.2 ± 4.4 | | | 77.1 ± 3.9 | | 77.3 ± 4.0 | | |  |  |  |  |  |
|  |  |  | DRY | | 19.5 ± 4.8 | | | 3.6 ± 2.0 | | 4.1 ± 2.4 | | 4.5 ± 2.3 | | 82.4 ± 6.7 | | | 79.5 ± 8.2 | | 77.5 ± 8.1 | | |  |  |  |  |  |
| Eye Movem. | | | WET | | 14.2 ± 2.4 | | | 3.0 ± 1.1 | | 4.2 ± 1.0 | | 4.1 ± 1.1 | | 78.7 ± 6.3 | | | 70.3 ± 5.9 | | 70.9 ± 5.8 | | |  |  |  |  |  |
|  |  |  | DRY | | 11.1 ± 2.4 | | | 3.1 ± 1.0 | | 3.7 ± 0.8 | | 3.7 ± 0.7 | | 70.8 ± 13.4 | | | 64.6 ± 12.4 | | 64.3 ± 13.8 | | |  |  |  |  |  |
| Myogenic artifact | | | WET | | 27.4 ± 8.2 | | | 14.6 ± 7.0 | | 10.7 ± 4.7 | | 10.4 ± 3.7 | | 49.1 ± 13.0 | | | 61.0 ± 10.7 | | 62.3± 6.0 | | |  |  |  |  |  |
|  |  |  | DRY | | 19.8 ± 5.3 | | | 11.3 ± 4.9 | | 8.9 ± 5.6 | | 8.8 ± 5.2 | | 44.5 ± 15.3 | | | 55.7 ± 23.3 | | 55.5 ± 21.8 | | |  |  |  |  |  |
| Cardiac interfer. | | | WET | | 5.5 ± 1.8 | | | 4.4 | | 2.5 ± 0.2 | | 2.6 ± 0.6 | | 37.7 | | | 40.6 ± 20.7 | | 50.3± 21.6 | | |  |  |  |  |  |
|  |  |  | DRY | | 3.9 ± 2.3 | | | 1.7 ± 0.8 | | 1.3 | | 3.4 ± 2.8 | | 35.4 ± 29.8 | | | 49.1 | | 33.2 ± 20.8 | | |  |  |  |  |  |
